# An Integrated Bioinformatics Analysis towards the Identification of Diagnostic, Prognostic, and Predictive Key Biomarkers for Urinary Bladder Cancer

**DOI:** 10.3390/cancers14143358

**Published:** 2022-07-10

**Authors:** Michail Sarafidis, George I. Lambrou, Vassilis Zoumpourlis, Dimitrios Koutsouris

**Affiliations:** 1 Biomedical Engineering Laboratory, School of Electrical and Computer Engineering, National Technical University of Athens, 9 Iroon Polytechniou Str., 15780 Athens, Greece; dkoutsou@biomed.ntua.gr; 2 Choremeio Research Laboratory, First Department of Pediatrics, National and Kapodistrian University of Athens, 8 Thivon & Levadeias Str., 11527 Athens, Greece; glamprou@med.uoa.gr; 3 University Research Institute of Maternal and Child Health and Precision Medicine, National and Kapodistrian University of Athens, 8 Thivon & Levadeias Str., 11527 Athens, Greece; 4 Biomedical Applications Unit, Institute of Chemical Biology, National Hellenic Research Foundation, 48 Vas. Konstantinou Ave., 11635 Athens, Greece; vzub@eie.gr

**Keywords:** bladder cancer, urologic cancer, diagnosis, prognosis, prediction, biomarkers, hub genes, bioinformatics, microarrays, meta-analysis

## Abstract

**Simple Summary:**

Bladder cancer is evidently a challenge as far as its prognosis and treatment are concerned. The investigation of potential biomarkers and therapeutic targets is indispensable and still in progress. Most studies attempt to identify differential signatures between distinct molecular tumor subtypes. Therefore, keeping in mind the heterogeneity of urinary bladder tumors, we attempted to identify a consensus gene-related signature between the common expression profile of bladder cancer and control samples. In the quest for substantive features, we were able to identify key hub genes, whose signatures could hold diagnostic, prognostic, or therapeutic significance, but, primarily, could contribute to a better understanding of urinary bladder cancer biology.

**Abstract:**

Bladder cancer (BCa) is one of the most prevalent cancers worldwide and accounts for high morbidity and mortality. This study intended to elucidate potential key biomarkers related to the occurrence, development, and prognosis of BCa through an integrated bioinformatics analysis. In this context, a systematic meta-analysis, integrating 18 microarray gene expression datasets from the GEO repository into a merged meta-dataset, identified 815 robust differentially expressed genes (DEGs). The key hub genes resulted from DEG-based protein–protein interaction and weighted gene co-expression network analyses were screened for their differential expression in urine and blood plasma samples of BCa patients. Subsequently, they were tested for their prognostic value, and a three-gene signature model, including *COL3A1*, *FOXM1*, and *PLK4*, was built. In addition, they were tested for their predictive value regarding muscle-invasive BCa patients’ response to neoadjuvant chemotherapy. A six-gene signature model, including *ANXA5*, *CD44*, *NCAM1*, *SPP1*, *CDCA8*, and *KIF14*, was developed. In conclusion, this study identified nine key biomarker genes, namely *ANXA5*, *CDT1*, *COL3A1*, *SPP1*, *VEGFA*, *CDCA8*, *HJURP*, *TOP2A*, and *COL6A1*, which were differentially expressed in urine or blood of BCa patients, held a prognostic or predictive value, and were immunohistochemically validated. These biomarkers may be of significance as prognostic and therapeutic targets for BCa.

## 1. Introduction 

### 1.1. Bladder Cancer towards Biomarker-Directed Management

Bladder cancer (BCa) is any of the various types of cancer that arise from the urinary bladder lining. BCa is a complex and heterogeneous disease that requires intensive surveillance owing to its high global prevalence, recurrence rate, as well as poor prognosis of invasive disease [1,2]. BCa constitutes the most common neoplasm of the urinary tract and is estimated to be the fourth most frequent malignancy in males, with a male-to-female preponderance of at least three to one [3,4]. In the American Cancer Society’s latest annual report, it is stated that an estimated number of 81,180 new cases of BCa will be diagnosed in the USA in 2022 and 17,100 people will die due to the disease this year [5]. BCa is primarily categorized into non-muscle-invasive BCa (NMIBC), which pertains to approximately 70–75% of diagnoses, and muscle-invasive BCa (MIBC), which refers to the other 25–30%. The two subtypes differ genetically and are related to distinct prognoses [6].

Advanced age, male sex, cigarette smoking, and chemical exposure contribute to the development of BCa [2]. There is currently no routine screening test recommended for the general public or for people at average risk [7]. The main diagnostic tools for symptomatic or people at increased risk are cystoscopy—which constitutes the gold standard for the evaluation of the lower urinary tract—and urine tests, such as urine cytology and urinalysis. In view of the fact that cystoscopy is an invasive and high-cost method, and cytology is restricted by poor sensitivity, particularly for early-stage and low-grade tumors [8], new urine tests for tumor biomarkers, which were found to partially overcome these limitations, have emerged. These tests investigate certain substances in urine, called extracellular vesicles (EVs), which comprise a new promising source of diagnostic and prognostic biomarkers in liquid biopsies [9]. However, they often lack sensitivity and specificity, in particular for low-grade and early-stage BCa tumors and recurrent diagnoses, and return many false positive results [10,11]. For this reason, these tests have not substituted the current diagnostic standards of cystoscopy and cytology [12].

Over the past years, many efforts have been stepped up to identify high-value molecular markers for BCa. There is no doubt that, although the exact molecular mechanisms underlying the progression of BCa remain unclear [13], we have greatly broadened our comprehension of the BCa molecular pathology, which has allowed us to establish new prognostic and predictive biomarkers using high-throughput technologies. However, there is still no biomarker approved for clinical practice, and advances in the treatment of BCa are lacking as opposed to those in other cancers [14]. On that account, the need to develop reliable and non-invasive methods to detect and predict BCa biological behavior is indispensable and still ongoing [15].

### 1.2. Reuse of Public Genome-Wide Gene Expression Data

The growing use of high-throughput technologies for gene expression analysis for the past two decades and the deposition of the vast majority of research data in public repositories have created a wealth of publicly available archives [16]. All these data offer an invaluable resource for reuse so that scientific findings and new knowledge can be introduced. In particular, the data integration approach from multiple experimental studies allows for increasing the sample size, the statistical power, and the robustness of the results [16,17], as well as improving reproducibility and the relevance of the biological information extracted [18].

The motivation of this study was to identify key hub genes serving as potential diagnostic, prognostic, and predictive biomarkers for BCa. On this basis, the aim of the first part of our analysis was to reuse all the available microarray-based gene expression data and carry out an integrative meta-analysis in order to assess the alterations of gene expression in urinary bladder tissue and to identify key hub genes in BCa. In this context, we also incorporated gene expression data derived from urine and blood samples in order to further investigate the potential altered expression of the identified key hub genes in these biological fluids. Subsequently, we conducted a survival analysis in order to assess the prognostic value of the key hub genes and constructed a prognostic model for BCa. The performance of the developed model was validated using two independent datasets as well as an online bioinformatics tool. In addition, we included data from MIBC patients receiving preoperative cisplatin-based chemotherapy to explore the predictive value of the hub genes in terms of therapy response and to construct a predictive model for BCa which was validated onto two external datasets. Finally, we resulted in a nine-gene panel of potential key biomarkers for BCa and equipped machine learning techniques [19] in order to deepen our research results and validate its diagnostic performance. We believe that these biomarkers could be promising diagnostic and prognostic targets for the management and treatment of BCa.

## 2. Materials and Methods

### 2.1. Overall Study Design and Workflow 

**Figure 1:**
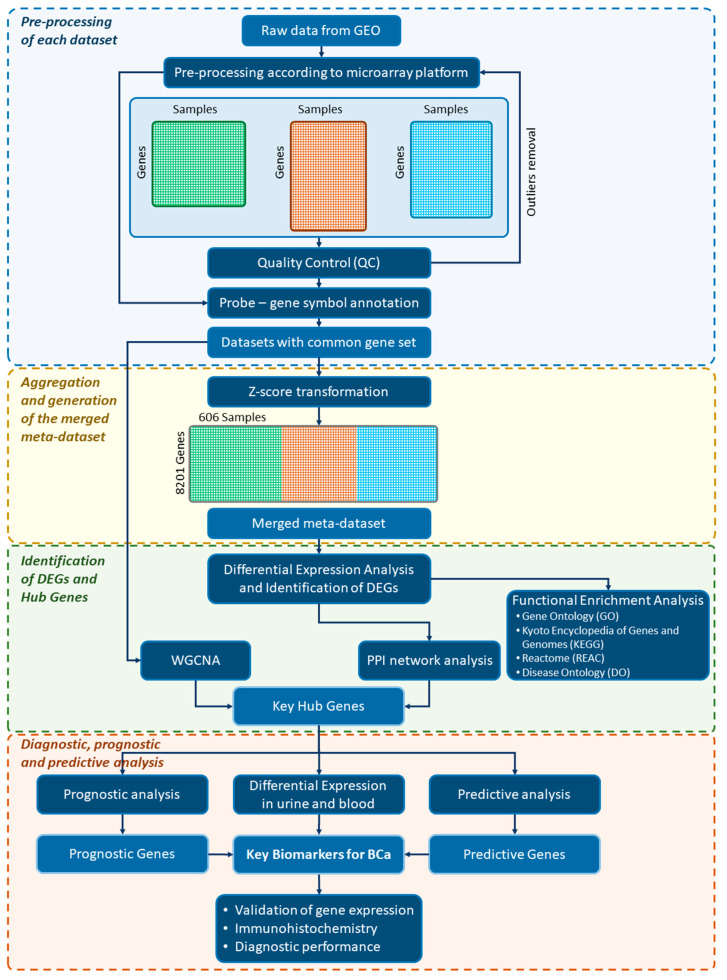
The overall study design and workflow of the integrative bioinformatics analysis of this study.

The overall design and pipeline of our integrative bioinformatics meta-analysis is presented in Figure 1. In the first phase, this study aggregated multiple microarray datasets and after the creation of a merged meta-dataset, it identified the differentially expressed genes as well as the key hub genes for BCa, through protein–protein interaction and weighted gene co-expression network analyses. In addition, functional analysis of differentially expressed genes was performed using the Gene Ontology (GO), the Kyoto Encyclopedia of Genes and Genomes pathways (KEGG), the Reactome (REAC) knowledge base, and the Disease Ontology (DO). Subsequently, the identified key hub genes were assessed for their diagnostic, prognostic, and predictive value. Towards this goal, urine- and blood-based gene expression data were incorporated, as well as survival data from BCa patients with various stages, and from MIBC patients receiving neoadjuvant chemotherapy. The key hub genes that were significantly expressed in urine or blood plasma and concurrently held a prognostic or predictive value were considered as potential key biomarkers. Finally, these biomarkers were validated for their expression in BCa and also evaluated for their diagnostic performance in multiple datasets.

### 2.2. Data Source, Systematic Search, and Selection of Eligible Microarray Datasets

All the microarray datasets used are publicly available and were derived from the Gene Expression Omnibus (GEO) at the National Center for Biotechnology Information (NCBI), which is the largest public repository designed for archiving and distributing microarray, next-generation sequencing, and other functional high-throughput genomic data [20].

**Figure 2:**
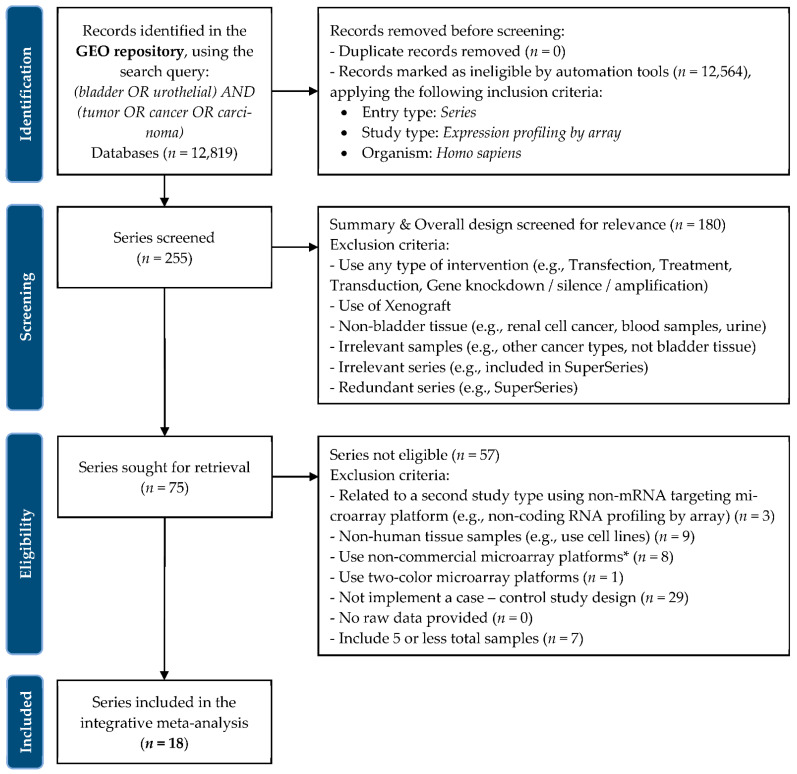
Preferred reporting items for systematic reviews and meta-analyses (PRISMA 2020) flow diagram. * An array identical to a commercial platform in which a custom, remapped CDF environment is used to extract data, was considered as a commercial platform.

We conducted a systematic search in the GEO repository entering the following query: “(bladder OR urothelial) AND (tumor OR cancer OR carcinoma)”. Additionally, we applied the following filter criteria: “Series”, “Expression profiling by array”, and “Homo sapiens” as entry type, study type, and organism, respectively. We obtained a total of 255 datasets from the inception up to 10 January 2022. A dataset was encompassed in the meta-analysis if the following main inclusion criteria were fulfilled: (1) implemented a case-control study design; (2) conducted using a single-color commercial microarray platform; (3) performed on human samples and derived from a lower urinary tract tissue (i.e., bladder or urethra); (4) performed only on untreated samples. We performed this meta-analysis conforming to the guidelines provided by the Preferred Reporting Items for Systematic Reviews and Meta-Analysis (PRISMA) statement published in 2020 [21]. The details of the selection process, including the complete criteria and workflow implemented, are depicted in Figure 2. Each dataset was independently checked by two authors (M.S. and G.I.L.).

The studies included in this meta-analysis examined human cancerous and normal urinary bladder tissues and were conducted using commercial platforms (Affymetrix, Illumina, and Agilent) for reproducibility and consistency reasons [22]. In addition, the selection of single-color arrays allowed us to conduct an integrative “early stage” approach [23], setting aside the increased complexity of incorporating data from two-color arrays.

For some datasets the initial number of samples was higher than the samples ultimately included in our study and the reasons for ruling some of them out are specified as follows: the 24 samples from the series GSE37815 were all included in series GSE13507; therefore, they were removed from the latter series. Moreover, the series GSE38264 included 13 samples from the organism *mus musculus*, and, thus, they were excluded from the series. Finally, the series GSE40355 included 24 samples that were obtained using a non-coding RNA microarray platform (Agilent Human miRNA Microarray V2) and they were consequently removed from this series.

### 2.3. Platform-Specific Pre-Processing

After identifying the eligible datasets for our study, we retrieved the raw microarray expression data for each dataset from GEO. Then, normalization was conducted in order to adjust the technical and environmental effects on the data. This procedure allows samples from a common study to be on a similar scale. The normalization process was performed in accordance with the dataset’s microarray platform.

The normalization of the Affymetrix datasets was performed by using the Robust Multiarray Analysis (RMA) algorithm, within the R/Bioconductor packages *affy* (version 1.72) [24], for the *HG-U133A* and *HG-U133 Plus 2* platform types, and *oligo* (version 1.58) [25], for the *HuGene-1.0 ST*, *HuEx-1.0 ST* and *HTA-2.0* platform types. This algorithm performs background correction, log2 data transformation, quantile normalization, and summarization of all probe sets into a single expression value for each gene. It has been shown to perform very well in terms of sensitivity to biological variation and to improve cross-platform comparability [26,27].

The normalization of the Illumina datasets was conducted by utilizing methods implemented in the R/Bioconductor package *limma* (version 3.50) [28], using the *read.ilmn* and *neqc* functions which read the Illumina expression data and perform background correction, log2 transformation, quantile normalization, using negative and positive control probes for normalization, and only negative controls for background correction. In datasets GSE13507 and GSE37815, the control probes have been removed from the non-normalized data; hence, we utilized the *read.table* and *normalizeBetweenArrays* functions of the same R package to properly read the raw data and perform the above-described steps.

Finally, the normalization of the Agilent datasets was also conducted by utilizing R/Bioconductor package *limma* methods. Raw data were read, background corrected (using the method *normexp*), log2 transformed, and quantile normalized, using the functions *read.maimages*, *backgroundCorrect*, and *normalizeBetweenArrays* within the package *limma*. The recommendations for the commonly found two-color Agilent arrays were followed since the same procedure applies and corresponds to a similar error model [29].

### 2.4. Quality Control

For all datasets, a common quality control (QC) non-platform-dependent analytical framework was applied for consistency reasons. After the corresponding pre-processing, each normalized dataset underwent QC implementing the outlier removal strategy. QCs were conducted using the R/Bioconductor package *arrayQualityMetrics* (version 3.50) [30], inspecting three visualizations included in the *arrayQualityMetrics* reports: the heatmap of the distance between arrays, the boxplot of the logarithm ratios, and the MA plot, which includes the logarithm of the intensity ratios (M) vs. average log intensities (A). This strategy has been shown to improve the efficacy of the meta-analysis and to increase the power of differentially expressed gene detection [31]. Samples classified as outliers in at least two of the three metrics during the quality control process were removed from their dataset. Subsequently, the raw data without outliers were normalized de novo, following the process described in the previous section, and were used for the downstream analysis.

### 2.5. Gene Annotation

All probes were mapped at the gene level using gene symbols as the common identifier across platforms. The official gene symbols are approved by the HUGO Gene Nomenclature Committee (HGNC) [32]. The use of HGNC-approved nomenclature is recommended since it is well curated and has been previously shown to enhance accuracy in scientific and public communication [33]. If more than one probe was mapped to the same gene symbol, the final expression level of this gene was calculated as the average expression values of the different probes. Probes with annotations for more than one gene or with no annotations were excluded from our study.

The mapping between probe sets and corresponding gene symbols was performed through particular annotation packages for each array model provided in the Bioconductor repository. The conversion between probes and gene symbols was achieved by the R/Bioconductor packages *AnnotationDbi* (version 1.56.2) and *org.Hs.eg.db* (version 3.14). In particular, the datasets were annotated using the R/Bioconductor packages *hgu133a.db*, *hgu133plus2.db* (version 3.13), *hugene10sttranscriptcluster.db*, *huex10sttranscriptcluster.db*, *hta20transcriptcluster.db* (version 8.8), *illuminaHumanv2.db*, *illuminaHumanv3.db*, and *illuminaHumanWGDASLv4.db* (version 1.26), depending on the platform. For the three series, GSE21142, GSE24152, and GSE42089, the corresponding custom brainarray chip description file (CDF) was utilized for annotation. For the Agilent platforms, the probe-gene mapping was conducted utilizing the R/Bioconductor package *biomaRt* (version 2.50.3) in order to access the *Ensembl* annotation [34]. The selection of these gene annotation resources was based on their constant updates, consistency, and reliability [34,35,36]. It is essential that the probe set annotations are updated and reliable so that biological inferences can be made accurately throughout the downstream analysis.

### 2.6. Batch Effects and Cross-Platform Normalization

Gene expression levels may vary due to biological factors in conjunction with non-biological ones, i.e., technical sources of variation, which are time- and place-dependent. These sources of variation, which are irrelevant to inter- and intra-sample class differences, are almost inevitable and summarily termed “batch effects” [37]. On account of them, the data integration from diverse microarray gene expression experiments, which are conducted in this study, becomes a complicated procedure [38].

The information on the batch numbers or the date of experiments is not available for many of the 18 datasets of our integrative meta-analysis, so applying a method that adjusts data for known batches is unfeasible. In order to perform a batch effect detection, a visual inspection of dimension-reduced data representations, using principal component analysis (PCA), was conducted. It needs to be mentioned that, due to the detection of a very strong batch effect, samples from GSE13507 were further separated into two subgroups, GSE13507A and GSE13507B, respectively. These two subgroups were considered distinct datasets during our integrative meta-analysis.

The Z-score transformation or standardization was applied to gene expression data, using the *scale* function in R package *stats*. The application of this classical normalization method constitutes an approach to standardizing data over a broad range of experiments and allows the microarray data juxtaposition regardless of the initial hybridization intensities [39]. In addition, the Z-score transformation is simple, it has low time and memory complexity, it does not require any assumption on data distribution, and it has been implemented successfully in previous studies indicating high performance [40,41,42]. Z-score transformation was applied to all samples by subtracting each sample’s mean and dividing by its standard deviation (SD), according to the formula:

(1) $$Z_{score}=\frac{I_{G}-{\overset{-}{I}}_{G_{1}\ldots G_{n}}}{\sigma }$$

where $I_{G}$ represents the intensity of gene *G*, and ${\overset{-}{I}}_{G_{1}\ldots G_{n}}$ and σ represent the mean intensity and standard deviation of the aggregate measure of all genes within a sample.

After the simple data homogenization, a further removal of, or adjustment for, batch effects was not attempted as it could systematically induce incorrect group differences, especially for our analysis where the batch–group design is unbalanced. Instead, it is recommended that, when possible, the batch variables should be incorporated into the downstream analysis [43]. Therefore, during the differential analysis, we incorporated each sample’s dataset as a covariate.

### 2.7. Differential Expression Analysis

In our integrative meta-analysis, we followed an “early stage” data integration method [23]. We created a merged microarray meta-dataset by binding the Z-score transformed samples and by matching the 8201 common gene symbols. This meta-dataset contained a total of 606 samples, incorporating 410 BCa samples and 196 control samples, across 19 different datasets.

The differentially expressed genes (DEGs) between BCa and normal tissue samples were screened using the R/Bioconductor package *limma* (version 3.50) [28], with the dataset/series of microarrays included as a covariate in the model. For the significance analysis, the main statistic used was the moderated t-statistic, which was computed for each gene symbol between cancer and control samples. In order to control the false discovery rate (FDR) for multiple comparisons, the *p*-value was adjusted based on the Benjamini–Hochberg (BH) method.

The statistical methods used to identify DEGs depend on the determination of arbitrary thresholds for *p*-value and fold change (FC) of expression levels, which can significantly alter microarray interpretations [44]. In our analysis, we used a stringent threshold for an adjusted *p*-value of 0.01. The cut-off threshold for |log_2_FC| is usually chosen between one and two. For the 11 different values of |log_2_FC| from one to two in steps of 0.1, 11 different sets of DEGs were obtained. It needs to be noted that the log_2_FC value for each gene that resulted from *limma* was corrected by dividing by the SD of the mean group differences for all genes, according to the formula:

(2) $${log}_{2}FC_{corrected}=\frac{{log}_{2}FC}{\sigma_{Z-score\;differences_{G_{1}\ldots G_{n}}}}$$

where $\sigma_{Z-score\;differences_{G_{1}\ldots G_{n}}}$ represents the SD of the mean Z-score differences between the two experimental conditions (cancer versus control group) for all genes [39].

In order to find the optimal set of DEGs that led to a more robust classification of samples, a support vector machine (SVM) model was established using DEGs as features. The SVM is one of the most popular supervised learning algorithms and has demonstrated a high ability to handle high-dimensional data and superior performance in the microarray classification of cancers [45]. In particular, an SVM model was built for each of the 11 sets of DEGs, implemented using the R package *caret* (version 6.0). For every set of features, the merged meta-dataset was split into a training set and a test set in the ratio of 90:10 and a random manner, and this procedure was iterated 10 times, implementing a 10-cross fold validation. The value of |log_2_FC|, and by extension the set of DEGs, which resulted in the higher area under the receiver operating characteristic (ROC) curve (AUC) of the classifier was selected. The ROC curve is a probability plot that features the true positive rate (sensitivity) against a false positive rate (1—specificity) at various threshold settings and constitutes an evaluation plot for binary classification problems. The AUC is a metric for the classifier’s ability to discriminate between classes and for the classification performance evaluation.

After the definition of the cut-off criteria, the set of DEGs was obtained and the volcano plot, as well as the heatmap for the first 100 DEGs, were plotted by implementing the R packages *ggplot2* (version 3.3.5) and *ComplexHeatmap* (version 2.10), respectively.

### 2.8. DEG Functional Enrichment Analysis

In order to analyze and visualize functional profiles of the identified DEGs, the R/Bioconductor package *clusterProfiler* (version 4.2.2) [46] was utilized. Gene Ontology (GO), Kyoto Encyclopedia of Genes and Genomes (KEGG) pathway, Reactome (REAC) pathway, and Disease Ontology (DO) enrichment analyses were conducted. Before performing enrichment analyses, the gene symbols were mapped to the Entrez Gene database in NCBI to retrieve Entrez Gene IDs, using the Bioconductor annotation *org.Hs.eg.db*. For all the following analyses, the cut-off thresholds were *p*-valueCutoff = 0.01 and q-valueCutoff = 0.05, corrected using the BH method.

The GO knowledgebase is the most extensive information resource regarding gene functions [47]. GO enrichment analysis covers three areas including cell component (CC), molecular function (MF), and biological process (BP), which were all included in our analysis. The GO terms for the down- and upregulated genes were enriched using the *enrichGO* function in the *clusterProfiler*. The GO terms were enriched by assigning OrgDb = “org.Hs.eg.db”, when running the *enrichGO*. Redundant enriched GO terms were removed using the *simplify* function, applying a threshold cut-off = 0.7 and the Wang method to measure the similarity [48]. Subsequently, the most significantly enriched terms were plotted using a bar plot.

KEGG is an integrated database for comprehending and associating higher-order functional information of the biological systems with genomic information [49]. KEGG pathway enrichment analysis was performed using the *enrichKEGG* function in the *clusterProfiler*. The corresponding Entrez Gene IDs of DEGs were imported and the aforementioned threshold criteria were implemented. The enrichment analysis was plotted using a dot plot.

REAC is a public, open-source, curated, and peer-reviewed pathway database that systematically relates human proteins to their molecular functions [50]. The REAC pathway analysis was performed against REAC (version 79), and the R/Bioconductor package *ReactomePA* (version 1.38) [51] was used. The pathway analysis was plotted using a dot plot, and an enrichment map of the results, based on the pairwise similarities of the enriched terms, was also visualized [52].

The DO represents a comprehensive knowledge base of over 10,000 inherited, developmental, and acquired human diseases [53]. For the DO enrichment analysis, the R/Bioconductor package *DOSE* (version 3.20.1) [54] was used. DO terms with more than minGSSize = 5 and less than maxGSSize = 500 genes annotated were tested, and from them, only those satisfying the cut-off thresholds were considered to be significantly enriched. The results were presented in the form of a dot plot.

### 2.9. Protein–Protein Interaction Network Analysis

A protein–protein interaction (PPI) network analysis was conducted to further explore the potential interaction between DEGs obtained from the integrative meta-analysis of the different datasets and to discover the key hub genes among them. The search tool for retrieval of interacting genes (STRING) database (version 11.5) [55], which incorporates both known and predicted PPIs, was employed to predict functional interactions between proteins. The PPI network of the 815 DEGs was created and visualized via the STRING web interface, applying a minimum required interaction score of 0.4.

In addition, the PPI network was imported into the Cytoscape software (version 3.9.1) [56]. The PPI network nodes were ranked performing 10 topological analysis methods from the cytoHubba plugin [57] in Cytoscape. These included three local-based methods, which are Maximal Clique Centrality (MCC), Maximum Neighborhood Component (MNC), and degree, as well as seven global-based methods, which are Edge Percolated Component (EPC), BottleNeck, EcCentricity, Closeness, Radiality, Betweenness, and Stress. A final ranking of the PPI network’s hub genes, based on the cytoHubba analysis, was obtained by utilizing the robust rank aggregation (RRA) method from the R package *RobustRankAggreg* (version 1.1). In the final ranking, only the hub genes with a *p*-value < 0.01 were kept.

Additionally, the MCODE (Molecular Complex Detection) plugin [58] of Cytoscape was used to determine gene clusters in the constructed network. The selection parameters were set as follows: MCODE scores ≥  7, degree cut-off  =  2, node score cut-off  =  0.2, max depth  =  100, k-score  =  2, and haircut = true. A gene list with all the genes belonging to the clusters that fulfill the above criteria was acquired.

Finally, the intersection of the two generated gene lists was calculated, in order to obtain a final list of hub genes based on the two Cytoscape plugins. The PPI network of the final hub genes list was also constructed.

### 2.10. Weighted Correlation Network Analysis

Weighted gene co-expression network analysis (WGCNA) can be used to construct a weighted gene co-expression network, define clusters (modules) of highly correlated genes, correlate modules with clinical traits, and identify intramodular hub genes [59]. In this study, we performed consensus WGCNA, using the R/Bioconductor package *WGCNA* [60], in order to find key gene modules that are highly associated with BCa. We utilized the individual datasets that were employed in the current integrative meta-analysis. Only the series that contained more than 20 samples were deployed, as datasets with fewer samples would simply be too noisy for the network to be biologically meaningful. Due to the fact that data came from different batches, which are unknown, we checked and adjusted for batch effects using the R/Bioconductor package *sva* (version 3.42) [61].

Gene expression values were hierarchically clustered for each of the datasets, in order to identify outliers and remove them from further analysis. For each dataset, the network topology analysis was performed. The intended scale-free topology fitting index threshold (R^2^) was set above 0.77 and the median connectivity was set below 30. After the selection of the proper soft thresholding power, the consensus modules across the datasets were calculated using the *blockwiseConsensusModules* function. The parameters were set as soft threshold power = 7, minModuleSize = 30, deepSplit = 2, and mergeCutHeight = 0.25, for merging the highly similar modules, and all the genes were processed into one block. Subsequently, for each dataset, the correlation matrix was converted into an adjacency matrix, which was analyzed further to compute the topological overlap matrix (TOM), using the TOM similarity algorithm. Based on the dissimilarity topological overlap calculation formula, the 8201 genes were assigned to distinct gene modules indicated by various colors.

Consequently, the correlation degree between each module’s eigengene (ME) and sample phenotype for each dataset was calculated by the Pearson correlation coefficient, using the *cor* function, and *corPvalueFisher* function for the calculation of the corresponding *p*-value. In order to find a consensus module–trait correlation, we formed a measure of module–trait relationship that summarized all the datasets into one measure: for each module–trait pair we obtained the correlation based on the shared correlation sign across datasets. Particularly, the lower absolute value was attributed to each consensus module–trait coefficient, if the correlations had the same sign, and a zero correlation for those with opposite signs. Hence, only modules with consistent correlation coefficients, either positive or negative, across datasets were considered key modules. The key gene modules were determined based on the correlation coefficient and the significance between the module’s ME values and sample traits (phenotypic group).

To further identify which genes in the key modules were highly associated with clinical traits, the correlation between sample phenotype, gene significance (GS), and module membership (MM) was evaluated. MM stands for the correlation between MEs and the profile of gene expression, and GS represents the correlation between genes and phenotypic traits. Thus, for every ME we calculated the GS and MM in each dataset, then, we combined the Z-scores of correlations from each dataset to form a consensus meta-Z-score and the corresponding meta-*p*-value for each module. Genes with high Z-scores for both MM and GS in the key module were highly interrelated with the cancer trait. Particularly, genes for which the MM and GS values were in the upper or lower quartile of all genes in the module were determined as hub genes for BCa. Finally, we compared these hub genes with the hub genes derived from the PPI network analysis in order to obtain the key hub genes of this study which were highly connected with BCa.

### 2.11. Differential Expression in Urine and Blood Plasma Samples

In order to explore the potential altered expression of the key hub genes, which derived from the gene expression analysis of urinary bladder tissues, in other biological fluids, urine and blood samples were included in our integrative meta-analysis. These samples underwent gene expression analysis by array, following a similar case-control study design to the meta-analyzed datasets in this study, and they were also downloaded from the GEO repository.

For the gene expression profiling in urine, we retrieved two datasets from the GEO repository, namely GSE51843 and GSE68020. The first dataset (GSE51843) includes a total of 11 mRNA-containing extracellular vesicle samples, 5 urine samples from BCa patients, and 6 samples from non-cancer patients, which were characterized by Illumina Human HT12 v4 BeadChip (GPL10558) [62]. The latter dataset (GSE68020) contains a total of 50 urine samples, including 30 high-grade urothelial carcinomas and 20 non-tumor healthy controls, which were characterized by the same microarray platform (GPL10558).

The raw data were downloaded for both datasets and platform-specific pre-processing was conducted, as was described in Section 2.3 for the other datasets. Implementing the *num.sv* function from the R/Bioconductor package *sva*, no surrogate variables were identified and, hence, no batch correction was needed. Each one of the key hub genes was tested for its statistically significant difference between the BCa and the control group in each dataset, using the Wilcoxon rank sum test which consists of a convenient and robust way to identify differentially expressed genes [63].

The blood dataset (GSE138118) includes a total of 75 samples, 11 newly diagnosed patients with BCa, 18 recurrence-negative formerly diagnosed BCa patients, 17 recurrence-positive formerly diagnosed BCa patients, and 29 healthy volunteers with no previous history of BCa or any other cancer. Total plasma RNA was isolated from clinical whole blood samples and was characterized by Affymetrix Human Gene 2.1 ST Array (GPL17692).

The raw expression data were downloaded and a platform-specific pre-processing was conducted, as was previously described in Section 2.3 for the other datasets. A batch effects removal was performed to minimize the unwanted variation on the data, using the *sva* function as implemented in the R/Bioconductor package *sva*, since it can be used without known batch variables. For this dataset, only the 28 BCa blood samples, from newly- or recurrence-positive formerly diagnosed patients, were kept along with the 29 control blood samples from healthy individuals. Each one of the key hub genes was tested for its statistically significant difference between the BCa and the control group, using the Wilcoxon rank sum test.

### 2.12. Finding Prognostic Genes for BCa

For the purpose of identifying which of the key hub genes hold a prognostic value, a survival analysis was conducted. Towards this purpose, the dataset GSE13507, which contains gene expression profile data from 165 patients with BCa of various stages (102 NMIBC and 63 MIBC) [64], was utilized. The clinical data of the patients are also available and contain information about the cancer-related events and the overall survival (OS) time. A univariate Cox regression analysis on the key hub genes was conducted to evaluate the association between cancer-specific OS of each patient and gene expression values, considering only genes with a *p*-value < 0.05. The R package *survival* (version 3.2) was used to conduct the univariate Cox regression analysis [65]. In order to select a panel of genes, and then build a prognostic multi-gene signature model, the least absolute shrinkage and selection operator (LASSO) Cox regression was performed, applying a 10-fold cross validation for 100 iterations, using the R package *glmnet* (version 4.1) [66]. Aiming to eliminate the selected gene correlation and prevent model overfitting, the gene coefficients were shrunk towards zero, by applying the minimum deviance lambda.min in each iteration and using Harrell’s C-index (concordance index) as the fit measure. The genes with nonzero coefficients for 75% of iterations were selected. In order to narrow the gene list down further and optimize the model, a multivariate Cox analysis was performed to identify the independent predictors for the prognosis of BCa patients and construct a prognostic index (*PI*) model. The PI was calculated based on the formula:

(3) $$PI=\overset{n}{\underset{i=1}{\sum }}c_{i}X_{i}$$

where $c_{i}$ is the coefficient of the *i*th gene, $X_{i}$ is the expression of the *i*th gene, and n is the number of the selected genes in the optimal model. The prognostic score was calculated for each patient, and the median score was defined as the cut-off value that stratified BCa patients into low- and high-risk groups to contrast their survival. The one-, three-, five-, and ten-year ROC curves were drawn along with AUC values for the evaluation of the model’s performance, using the R packages *survivalROC* (version 1.3.0) [67] and *plotROC* (version 2.2.1) [68]. To explore the relationship among the prognostic genes of this panel, we determined the Pearson correlation coefficient between all pairs. Finally, the R package *survminer* (version 0.4.9) was used to perform the Cox proportional hazards model analysis.

To investigate whether our prognostic model was applicable to other datasets and to validate its prognostic value, we obtained two independent microarray datasets from the GEO repository (GSE32894 and GSE32548), which incorporated gene expression data along with survival information of BCa patients. The GSE32894 contains a total of 224 primary BCa samples of various stages, which were characterized by Illumina HumanHT-12 V3.0 (GPL6947) [69]. The original dataset contains more samples but information about the survival events is available only for a subset of the original samples. The GSE32548 dataset includes a total of 131 primary BCa tumor samples, which were characterized by the same platform (GPL6947) [70]. Subsequently, the prognostic index was calculated for each patient of the two datasets. Based on this index, patients were divided into low- and high-risk groups, and Kaplan–Meier survival curves were generated to compare survival between the two groups by log-rank test, considering a *p*-value < 0.05 as statistically significant. The hazard ratios (HR) and 95% confidence intervals (CI) were also calculated. Time-dependent ROC analyses were conducted to evaluate the prognostic effectiveness of the prognostic risk score model.

Additionally, we utilized publicly available online bioinformatics tools to also access the prognostic value of the identified key hub genes. Gene Expression Profiling Interactive Analysis (GEPIA2) [71] is an open-access online tool for the interactive exploration of RNA sequencing data from The Cancer Genome Atlas (TCGA) [72] and the Genotype-Tissue Expression (GTEx) [73] programs. GEPIA2 was utilized for accessing the prognostic value of the key hub genes in terms of OS or disease-free survival (DFS) of the TCGA-BCa patients. The discovery TCGA-BCa cohort consists of 404 BCa patients and 19 controls. The difference between the survival rates of high- and low-expression groups for each key hub gene was contrasted using the log-rank test, considering statistical significance when the *p*-value < 0.05 and using the median or the quartile as cut-off criteria. The survival curves with the calculated HR and the log-rank *p*-value were plotted. Lastly, the GEPIA2 platform was utilized to confirm the prognostic validity of the gene signature generated by the multivariate Cox regression analysis and to plot the survival curves.

### 2.13. Finding Predictive Genes for BCa

Aiming to further investigate the predictive value of the key hub genes, samples from MIBC patients receiving preoperative cisplatin-based chemotherapy were included in our analysis. These samples underwent gene expression analysis by array and they were derived from the GEO repository. One of the aims of this study was to explore to what extent gene expression signatures can predict chemotherapy response. It is noteworthy that the current standard for MIBC is platinum-containing (e.g., cisplatin) neoadjuvant chemotherapy followed by radical cystectomy. Nonetheless, for many patients, there is a low chemotherapy success rate and several candidate biomarkers of therapy responsiveness are investigated [74].

The selected dataset (GSE169455) includes a total of 149 samples, which are all derived from MIBC patients receiving neoadjuvant cisplatin-based chemotherapy undergoing radical cystectomy [75]. RNA was extracted from bladder transurethral resection specimens and hybridized on an Affymetrix Human Gene 1.0 ST Array (GPL6244). The raw expression data were downloaded and a platform-specific pre-processing was conducted, as was previously described (Section 2.3). A batch effects removal was performed to minimize the unwanted variation on the data, using the *Combat* function from the R/Bioconductor package *sva* [76].

The main outcome measure was a pathological response in the cystectomy specimen, stratified as “complete response”, “partial response”, and “no response”. Each one of the key hub genes was tested for its statistically significant difference between the three response groups, using the Wilcoxon rank sum test. Furthermore, the univariate Cox regression analysis was conducted in order to calculate the association between each hub gene and the recurrence-free survival (RFS), cancer-specific survival (CSS), and overall survival (OS) of each patient, using the R package *survival* and considering a *p*-value < 0.05 as statistically significant. As univariate analysis resulted in a limited number of genes, the LASSO Cox regression for penalty parameter tuning (as described in Section 2.12) with 10-fold cross validation was performed to screen the key hub genes. The prevailing nonzero-coefficient genes were incorporated into the multivariate analysis, applying the Cox proportional hazards regression model, which resulted in a predictive gene signature. Finally, we successfully constructed a predictive risk score formula by using the corresponding coefficients of the gene signature (as in Section 2.12). The risk score divided the patients into low- and high-risk groups by the median value. The Kaplan–Meier survival curves were plotted for the two groups and time-dependent ROC curve analysis was performed based on the prediction risk score, and the AUC values were calculated to assess the prediction performance.

To validate the predictive value of our model, we acquired two independent microarray datasets (GSE87304 and GSE69795) from the GEO database, which included survival information of BCa patients recruited into a neoadjuvant trial. The GSE87304 contains 305 specimens from patients with MIBC, obtained by transurethral resection prior to pre-neoadjuvant chemotherapy, which was characterized by Affymetrix Human Exon 1.0 ST Array [77]. The GSE69795 contains 38 formalin-fixed paraffin-embedded bladder tumors, obtained by transurethral resection from BCa patients receiving neoadjuvant chemotherapy with dose-dense methotrexate, vinblastine, doxorubicin, and cisplatin along with bevacizumab, which was characterized by Illumina HumanHT-12 WG-DASL V4.0 R2 [78]. For both datasets, patients were divided into low- and high-risk groups, according to the predictive index, and Kaplan–Meier survival curves were plotted, including HR and 95% CI. Time-dependent ROC analysis was performed to evaluate the predictive effectiveness of the risk score model.

### 2.14. Expression Validation of Key Biomarkers and Immunohistochemistry

Based on the identified key hub genes and taking the above analysis into consideration, we opted for nine potential key biomarker genes that seem to play a significant role in the development and progression of BCa. All these biomarkers are significantly expressed in the urine or blood plasma of BCa patients and hold a prognostic or predictive value.

The GEPIA2 platform was utilized to confirm the expression alterations of the key biomarker genes. The external validation was done by comparing transcriptomic data from TCGA-BCa, TCGA normal, and GTEx datasets. The cut-off criteria |log_2_FC| > 1 and *p*-value < 0.05 were considered for a statistically significant difference. In addition, the association of the key biomarker genes with the pathological stages of BCa was performed through the GEPIA2 platform.

Further, the protein expression encoded by these biomarker genes was validated in BCa specimens using the Human Protein Atlas platform, which incorporates spatial proteomics and quantitative transcriptomics (RNA-Seq) data obtained from immunohistochemistry (IHC) analysis of tissue microarrays [79].

### 2.15. Diagnostic Performance Analysis

Using the identified key biomarker genes as features, we built and tested various classification models to access their diagnostic performance. Initially, all the datasets used in the current analysis and contained more than 10 samples were repurposed as training/test sets in order to validate the diagnostic ability of the nine features. Their diagnostic value was also evaluated in the final merged meta-dataset.

Finally, an external dataset was also utilized to further evaluate these features as diagnostic biomarkers for BCa. This dataset was obtained from the ArrayExpress repository at the European Bioinformatics Institute (EMBL-EBI) [80] and contained 19 BCa (14 NMIBC and 5 MIBC) and 11 control samples from urinary bladder tissue biopsies [81], which were hybridized to Affymetrix Human Gene 1.0 ST (GPL6244).

For every dataset, a fivefold cross-validation technique was implemented and repeated 10 times in order to get a more accurate evaluation of our classification model’s performance. For the final merged meta-dataset, a 10-fold cross validation was implemented. For all the developed models, the AUC of the classifier was used to evaluate the diagnostic performance of the model. The resulting ROC curves for all the built models along with their corresponding AUC values and 95% CIs were plotted. Each point on the ROC curves denotes a sensitivity/specificity pair obtained from a particular decision threshold, and the AUC indicates the efficacy of the corresponding model. The closer the AUC is to 1.0, the better the performance of the classification model.

## 3. Results

### 3.1. Systematic Search and Selection of Eligible Microarray Datasets

A total of 18 studies from GEO (accession numbers: GSE3167, GSE7476, GSE13507, GSE21142, GSE23732, GSE24152, GSE31189, GSE37815, GSE38264, GSE40355, GSE41614, GSE42089, GSE45184, GSE52519, GSE65635, GSE76211, GSE100926, and GSE121711) met the inclusion criteria (Figure 2) and were selected for the integrative meta-analysis. The final dataset included 619 samples (417 BCa samples and 202 controls). The datasets included in this meta-analysis followed a similar experimental design and compared human BCa tissues with normal ones. Notably, the datasets were characterized by 13 different microarray platforms. Table 1 provides detailed information on each dataset included in the integrative meta-analysis and highlights the sample type, their phenotypic characteristics, year, and reference of the study and microarray platform used.

### 3.2. Quality Control

In total, 13 samples were identified as outliers according to the implemented QC framework and they were consequently excluded from further analysis. More specifically, we removed two samples from the dataset GSE3167 (one BCa sample and one control), one sample from the dataset GSE13507 (BCa sample), one sample from the dataset GSE21142 (BCa sample), five samples from the dataset GSE31189 (two BCa samples and three controls), one sample from the dataset GSE38264 (control), two samples from the dataset GSE40355 (two BCa samples), and one sample from the dataset GSE42089 (control). After QC, the meta-analysis included 606 samples, consisting of 410 BCa samples and 196 controls.

### 3.3. Gene Annotation

Subsequent to the proper probe-gene mapping for each individual platform, we juxtaposed the coverage of the 13 different human arrays (*Affy HG U133A*, *Affy HG U133 Plus 2*, *Illu Human-6 V2*, *Affy HuGene 1 ST*, *Agi WHG 4x44K V2*, *Affy HuEx 1 ST*, *Agi G3 GE 8x60K*, *Illu Human-6 V3*, *Illu Human-12 WG-DASL V4*, *Affy HTA 2*, and three custom Brainarray CDFs for *Affy HG U133 Plus 2*). Overall, the probes on the 13 array platforms targeted a total of 27,579 unique gene symbols, out of which 8201 gene symbols were common among all 13 microarray platforms. Hence, the integrative meta-analysis was conducted only on these 8201 common gene symbols across all datasets.

### 3.4. Batch Effects and Cross-Platform Normalization

The batch effects presented in each dataset were inspected utilizing the PCA. Due to a very strong detected batch effect, samples from GSE13507 were further separated into two subgroups, GSE13507A and GSE13507B, with 41 (24 BCa samples and 17 controls) and 190 (145 BCa samples and 45 controls) samples, respectively (Figure S1). The new PCA plots for each of these two datasets are presented in Figure S2. These two subgroups were considered as individual datasets during the downstream analysis.

The Z-score transformation was used to correct intra-sample data and to adjust the systematic bias across datasets generated by different platforms. Therefore, the hybridization values for each gene within a sample are expressed in SD units from the zero mean. Comparisons across samples were then performed on uniformly transformed data.

### 3.5. Differential Expression Analysis

Following the pre-processing and standardization of each dataset, we combined the Z-score transformed expression data for every sample into a universal dataset by using the common gene symbols. This merged meta-dataset included 606 samples (410 BCa and 196 control samples) and 8201 shared gene symbols.

The DEGs between BCa and control tissue samples of the 19 merged datasets from the GEO were obtained using the R/Bioconductor package *limma*. The *p*-value was adjusted using the BH method in order to control the FDR, and a cut-off threshold of adjusted *p*-value < 0.01 was selected. In order to determine the |log_2_FC| cut-off value, an SVM classification model was established for each of the different sets of DEGs corresponding to the |log_2_FC| values from one to two in steps of 0.1. Thus, for every |log_2_FC| value the corresponding set of DEGs was used as features. For every model, the number of the features along with the estimated area under the ROC curve (AUC), the sensitivity, and the specificity of the classifier are presented in Table 2. The AUC actually expresses the probability value that one sample is classified correctly. As we notice, the classifier achieves very high classification precision and the differences are almost negligible.

**Figure 3:**
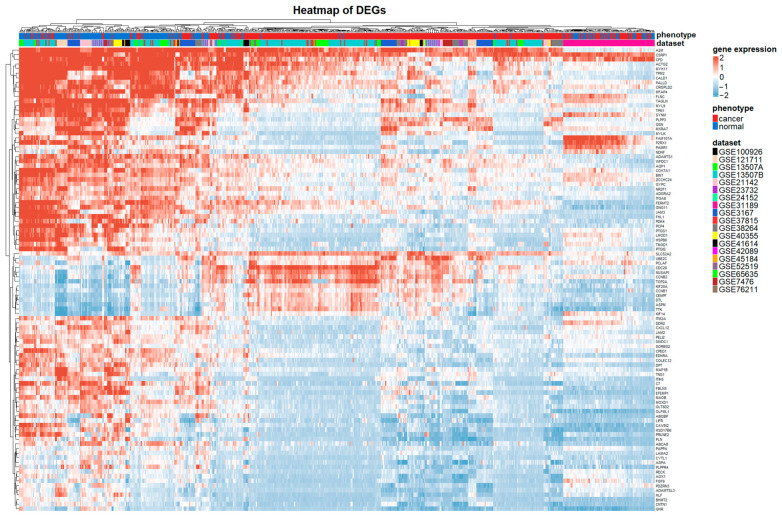
Heatmap plot of the top 100 DEGs between BCa and control samples in the merged meta-dataset. Blue and red represent relative downregulation and upregulation, and white represents no significant change in gene expression. Horizontal and vertical axes are clustered by genes and samples, respectively.

However, the highest value for AUC, which indicates the classifier with the highest performance, was achieved for the cut-off value of |log_2_FC| = 1.3. Therefore, a total of 815 DEGs between BCa and control samples were obtained through the expression profiles of the *limma* package, implementing |log_2_FC| ≥ 1.3 and adjusted *p*-value < 0.01 as cut-off criteria. Overall, the DEGs contained 540 downregulated genes and 275 upregulated genes. The volcano plot and the top 100 DEGs heatmap are illustrated in Figure S3 and Figure 3, respectively. In the generated heatmap, hierarchical clustering was performed on gene and on sample level as well. It can be observed that batch effects were present in the gene expression space, as samples were clustered based on their phenotype and microarray study.

### 3.6. Functional and Pathway Enrichment Analysis

To gain insight into the functional roles of DEGs and pathways involved in BCa, we performed a comprehensive functional enrichment analysis in various databases. In particular, GO, KEGG pathway, REAC pathway, and DO enrichment analysis of the 815 robust DEGs were performed, using the R/Bioconductor package *clusterProfiler*. For all the following analyses, the cut-off threshold parameters were *p*-valueCutoff = 0.01 and q-valueCutoff = 0.05, corrected using the BH method.

**Figure 4:**
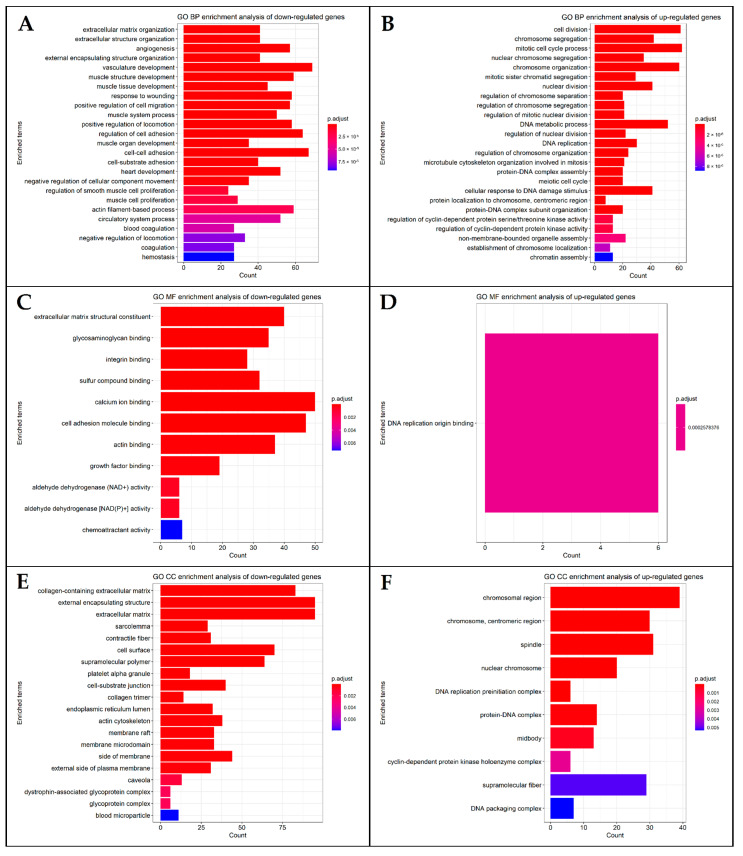
Top 25 significant Gene Ontology (GO) enrichment analysis terms of (**A**,**B**) biological processes (BP) of down- and upregulated DEGs; (**C**,**D**) molecular functions (MF) of down- and upregulated DEGs; (**E**,**F**) cellular components (CC) of down- and upregulated DEGs.

Gene Ontology (GO) enrichment analysis was performed based on the list of identified DEGs. The bar plots of the top 25, if present, enriched GO terms of biological processes (BP), molecular functions (MF), and cellular components (CC) were generated in the form of bar plots and are presented in Figure 4. GO terms of downregulated genes related to BP included extracellular matrix organization, extracellular structure organization, angiogenesis, vasculature, muscle structure, and muscle tissue development; GO terms of upregulated genes related to BP included cell division, mitotic cell cycle process, chromosome segregation, and organization. In the MF category, downregulated DEGs were enriched with extracellular matrix structural constituent, as well as glycosaminoglycan, integrin, sulfur compound, and calcium ion binding functions. The upregulated genes in the MF area were enriched only with DNA replication origin binding. CC GO terms of downregulated genes were primarily associated with the collagen-containing extracellular matrix, extracellular encapsulating structure, and extracellular matrix. Finally, CC GO terms of upregulated genes were enriched in the spindle, chromosome centromeric region, and chromosomal region.

**Figure 5:**
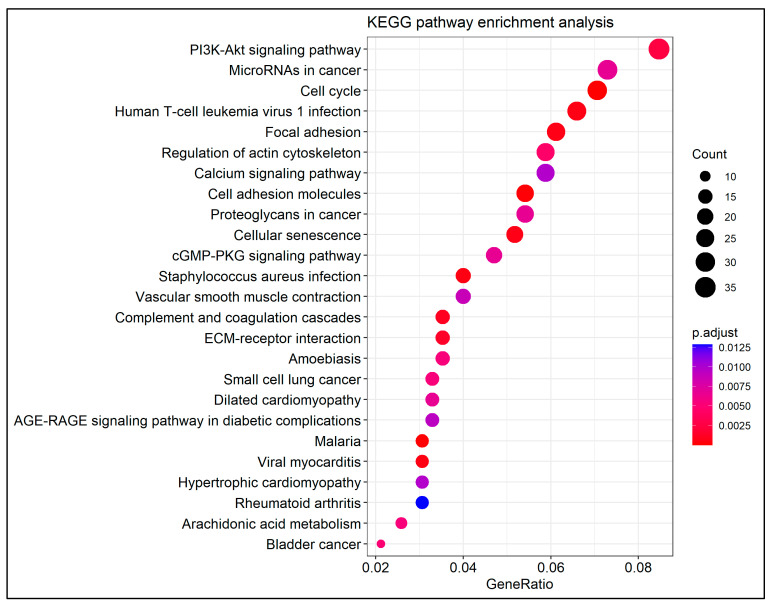
Top 25 significant terms of the KEGG pathway analysis.

KEGG pathway enrichment analysis demonstrated that there were 35 pathways enriched in the set of DEGs. The top 25 enriched terms are presented in Figure 5. The analysis showed that PI3K-Akt signaling pathway, micro-RNAs in cancer, cell cycle, focal adhesion, cell adhesion molecules, cellular senescence, complement, and coagulation cascades, ECM–receptor interaction, and bladder cancer were highly connected with the detected DEGs.

**Figure 6:**
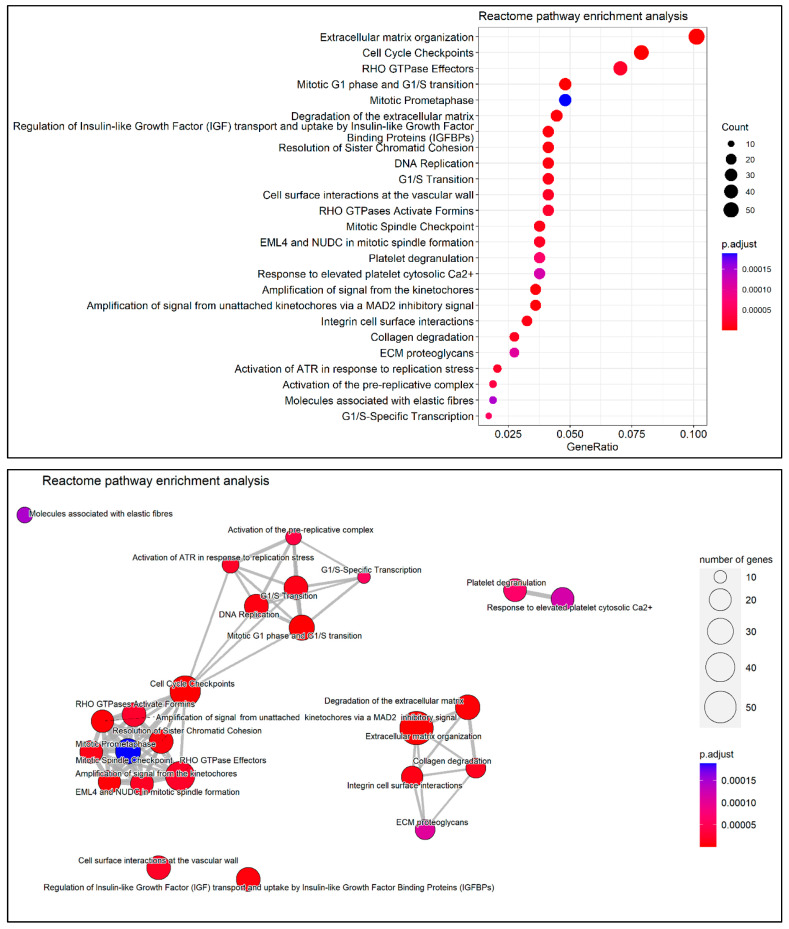
(**Upper**) Top 25 significant terms of the Reactome pathway enrichment analysis. (**Lower**) Enrichment map of the Reactome enriched terms presented into a network.

Through the REAC enrichment analysis, a number of 81 pathways were enriched. All of the top 25 pathways showed high significance for their respective entities and are presented in Figure 6. The most enriched REAC terms were extracellular matrix organization, cell cycle checkpoints, and Rho GTPase effectors, which are key regulators of cytoskeletal dynamics. The pairwise similarities of the enriched terms were also calculated and visualized in an enrichment map (Figure 6). In this map, two main clusters were defined, which were involved in the processes of cell cycle and replication and extracellular matrix, respectively.

**Figure 7:**
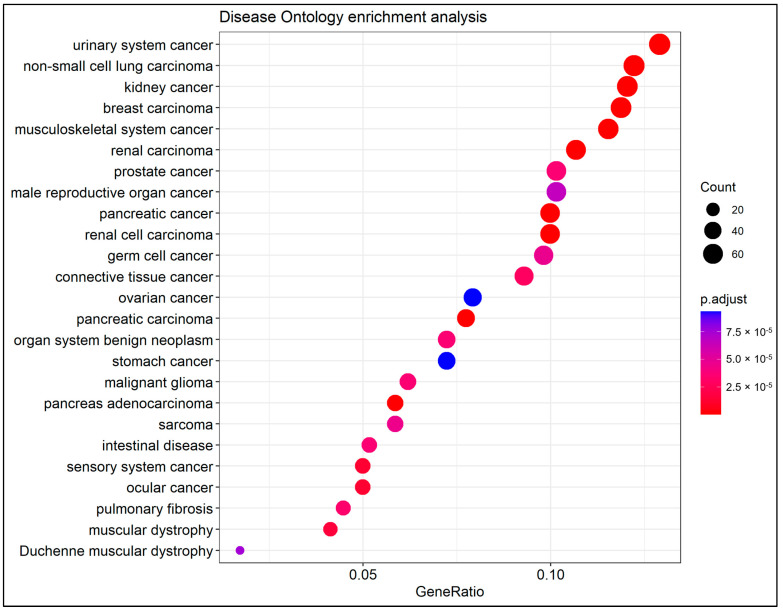
Top 25 significant terms of the Disease Ontology (DO) enrichment analysis.

DO enrichment analysis demonstrated that there were 185 enriched terms that were strongly connected with the detected DEGs. The top 25 enriched DO terms are presented in Figure 7. Noteworthy, the highest enriched DO term was the urinary system cancer, along with non-small cell lung, kidney, breast, and musculoskeletal system carcinomas in the following ranks.

### 3.7. Protein–Protein Interaction Network Analysis

**Figure 8:**
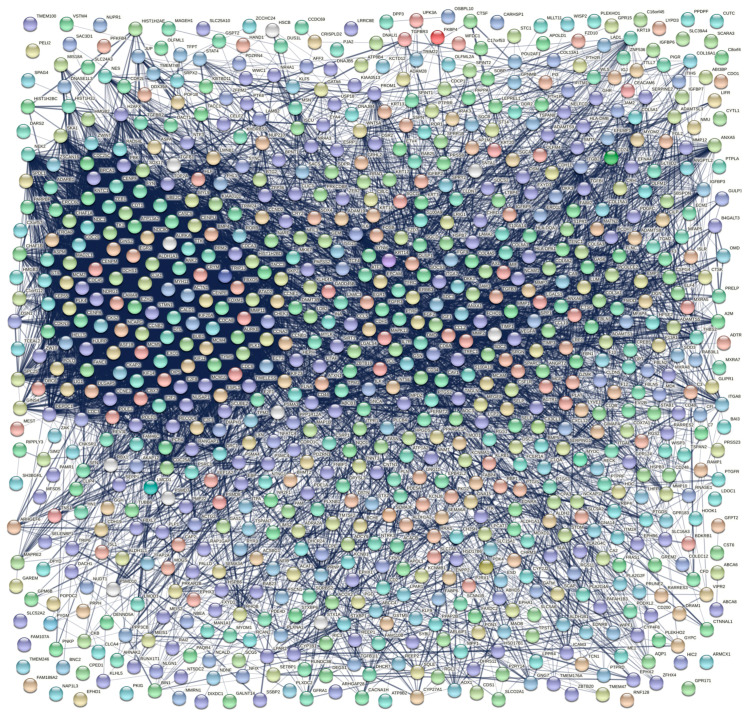
The constructed PPI network visualized by the STRING database. Nodes represent proteins and edges represent protein–protein associations. Line thickness represents the confidence score of a functional association.

The PPI network of the 815 DEGs was constructed and visualized via the STRING database (Figure 8). The network included 813 nodes and 8260 edges, with an average node degree of 20.3 and an average local clustering coefficient of 0.382. The PPI network’s enrichment *p*-value was < 10^−16^, indicating that the proteins were biologically related as a group.

The nodes of the PPI network were ranked applying 10 topological analysis methods and, hence, 10 ranked gene lists were obtained. These methods included local as well as global algorithms, as implemented in the cytoHubba plugin in Cytoscape software. In order to result in a final ranked gene list, we utilized the robust rank aggregation (RRA) method. The final ranked gene list, based on the cytoHubba plugin, included 129 genes, which showed a *p*-value < 0.01 (A in Table 3).

Furthermore, we performed cluster analysis utilizing the MCODE plugin in Cytoscape software and kept only clusters with a score of more than seven. According to this, the selected modules included 133 nodes and 3346 edges grouped in three clusters. Cluster one contained 83 nodes and 3045 edges, with a cluster score of 74.268. Cluster two contained 23 nodes and 198 edges, with a cluster score of 18. Finally, cluster three contained 27 nodes and 103 edges, with a score of 7.923. The whole node list for each of the three clusters, containing a total of 133 genes, is presented in B in Table 3.

**Figure 9:**
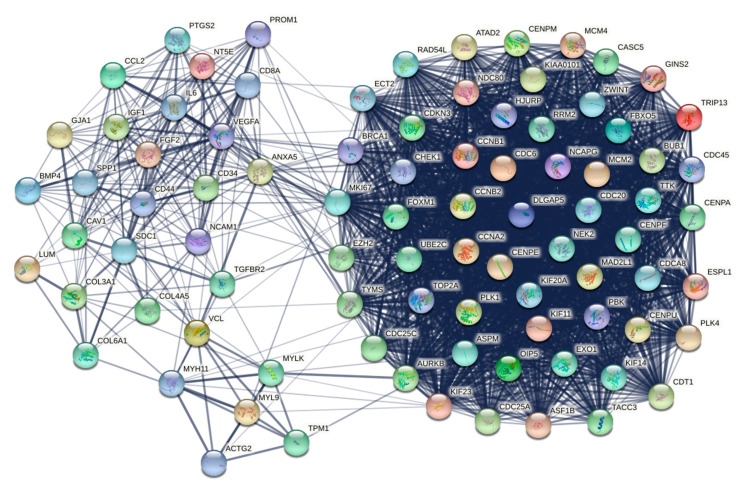
The PPI network of the final 87 hub genes. Nodes represent proteins and edges represent protein–protein associations. Line thickness represents the confidence score of a functional association.

The final list of hub genes, based on cytoHubba and MCODE plugins of Cytoscape, contained 87 common genes which were considered the most significant nodes of the PPI network (C in Table 3). The PPI network of these genes is presented in Figure 9.

### 3.8. Weighted Protein–Protein Interaction Network Analysis

In order to identify strongly BCa-correlated genes among the common cross-platform genes, a weighted gene co-expression network analysis (WGCNA) was conducted, including only the datasets containing more than 20 samples. Based on the hierarchical clustering trees, no outliers were detected, since they were removed in the previous steps (Section 3.2). After the batch effects correction through the *sva* function, a total of 482 samples, distributed across eight datasets, were used in this consensus network topology analysis.

We chose seven as the consensus suitable soft thresholding power, as this is the lowest power at which two conditions are fulfilled: the scale-free topology fit index reaches 0.77 and the median connectivity measurements decrease below 30 (Figure S4). Along with the threshold power, we set 30 as the minimal module gene size and 0.25 as the height for the dynamic tree cutting algorithm. Accordingly, we obtained eight consensus gene co-expression modules. The number of the included genes in each module ranged from 34 to 1901 (Figure S5); the gray module contained 4898 genes that could not be assigned to any module.

**Figure 10:**
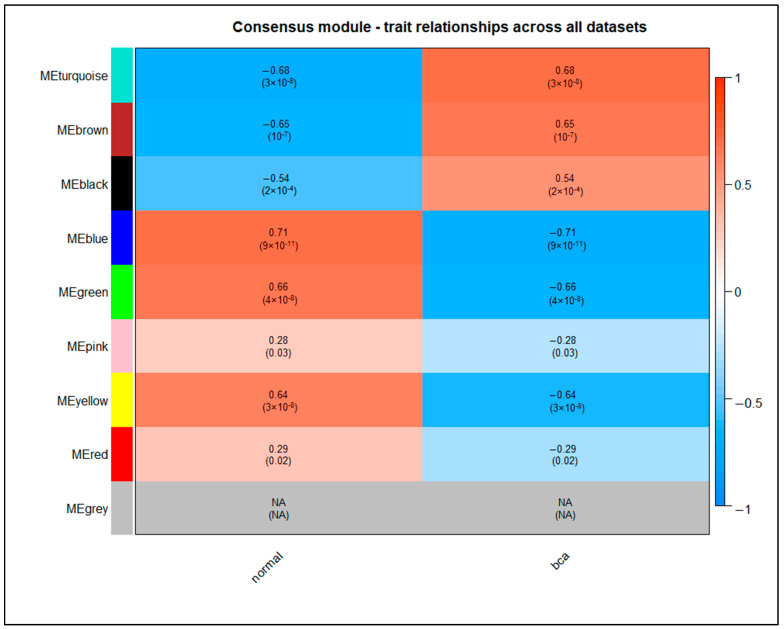
Heatmap of the consensus relationships of consensus module eigengenes and phenotypic traits. Each row corresponds to a consensus module eigengene and each column corresponds to the phenotypic characteristic. Each cell contains the corresponding correlation (ranging from blue to red) and *p*-value.

After obtaining gene modules, the correlation coefficients of each module for all eight datasets were calculated (Figure S6). In order to obtain a consensus module–trait correlation heatmap, only the modules with a consistent coefficient sign across all datasets were kept. For them, the lower absolute correlation value in all datasets and the higher *p*-value were assigned as the module’s consensus correlation coefficient and significance, respectively. For the remaining modules, the zero value was assigned as the consensus correlation (Figure 10). The advantage of the consensus relationship heatmap is that it isolates the module–trait relationships that are present in all datasets, and hence may be in a sense considered validated. It has to be noted that all the module–trait correlations and significance were very low for the GSE31189 dataset, presumably due to the strong batch effects that remained. Hence, this dataset was not considered for the consensus module–trait correlation. Based on the final heatmap of module–trait correlations, we determined that the turquoise (cor  =  −0.68, *p*-value  =  3 × 10^−8^), brown (cor  =  −0.65, *p*-value  =  1 × 10^−7^), black (cor  =  −0.54, *p*-value  =  2 × 10^−4^), blue (cor  =  0.71, *p*-value  =  9 × 10^−11^), green (cor  =  0.66, *p*-value  =  4 × 10^−8^), and yellow (cor = 0.64, *p*-value  =  3 × 10^−8^) modules were most highly correlated to BCa (*p*-value < 0.01) and were characterized as key modules (Figure 10). The turquoise module contained 1901 genes, the brown module included 764 genes, the black included 231 genes, the blue module encompassed 139 genes, the green module included 115 genes and, finally, the yellow module integrated 58 genes.

Next, we calculated the gene significances and module memberships in each key module. We set the criteria to specify the hub genes highly associated with BCa: module membership (MM) and gene significance (GS) meta-Z-scores in the upper or lower quartile of each module. We identified that 815 hub genes from the turquoise module, 345 hub genes from the brown, 91 genes from the black, 49 genes from the blue, 50 genes from the green, and 21 genes from the yellow module met the inclusion criteria (Figure S7). Finally, we combined the genes of each key module with the hub genes obtained from the PPI network analysis, and we determined the key hub genes of our analysis. These key hub genes are listed in Table 4.

### 3.9. Differential Expression in Urine and Blood Plasma Samples

**Figure 11:**
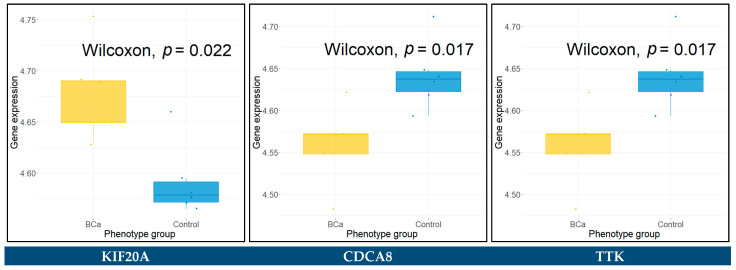
Significantly differentially expressed key hub genes in urine samples using the Wilcoxon rank sum test in GSE51843 (*n* = 11).

**Figure 12:**
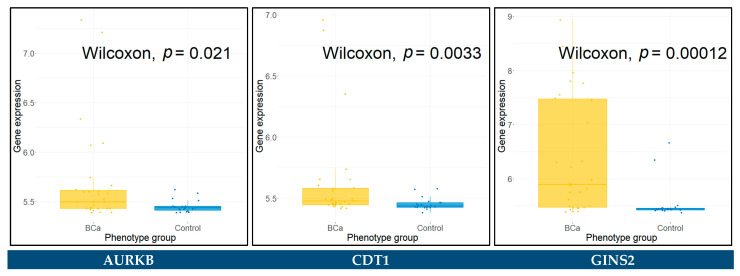

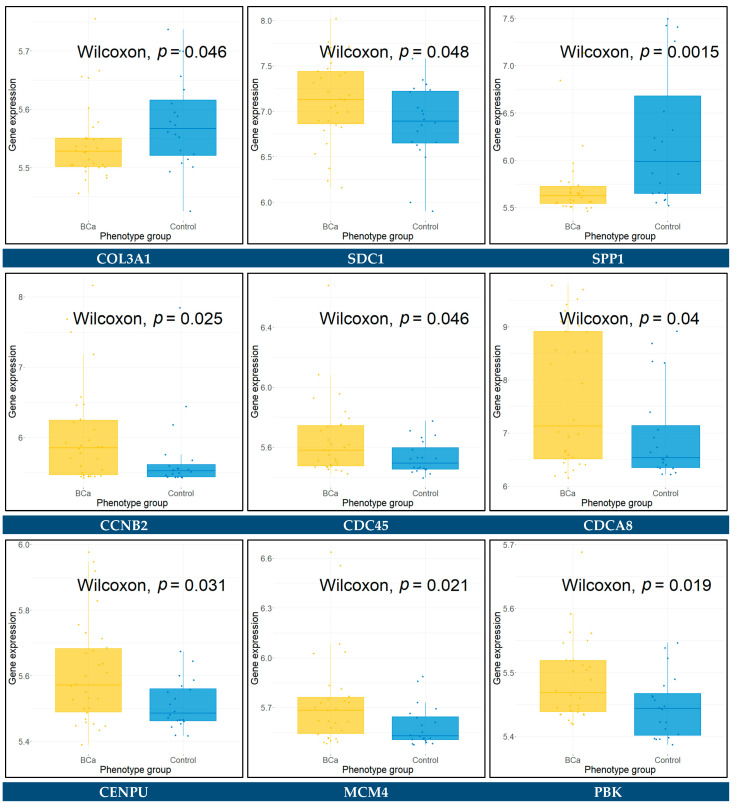

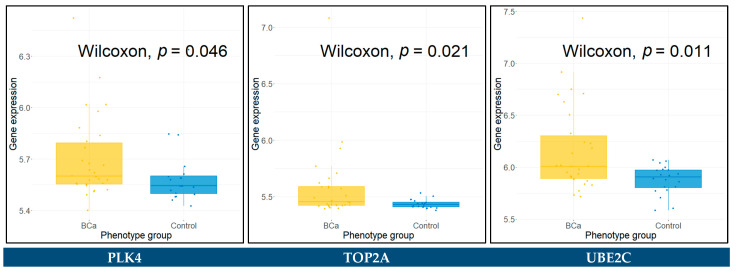
Significantly differentially expressed key hub genes in urine samples using the Wilcoxon rank sum test in GSE68020 (*n* = 50).

The raw gene expression data of urine samples, from series GSE51843 and GSE68020, characterized by Illumina Human HT12 v4 BeadChip (GPL10558), were downloaded and appropriately pre-processed. The datasets consist of five BCa and six control urine samples, and 30 BCa and 20 control urine samples, respectively. The Wilcoxon rank sum test was applied in each dataset to assess the statistical significance between the BCa and control groups for all the key hub genes. The hub genes with a *p*-value < 0.05 were identified as significantly differentially expressed between BCa and control urine samples. These genes included *KIF20A*, *CDCA8*, and *TTK* for the GSE51843, and *AURKB*, *CDT1*, *GINS2*, *COL3A1*, *SDC1*, *SPP1*, *CCNB2*, *CDC45*, *CDCA8*, *CENPU*, *MCM4*, *PBK*, *PLK4*, *TOP2A*, and *UBE2C* for the GSE68020 dataset. The Wilcoxon rank sum test results for these genes are presented in boxplots in Figure 11 and Figure 12.

**Figure 13:**
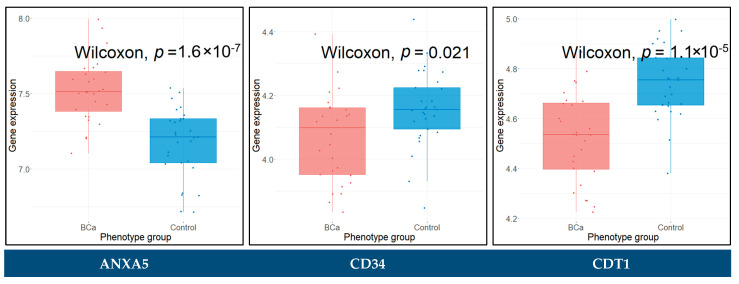

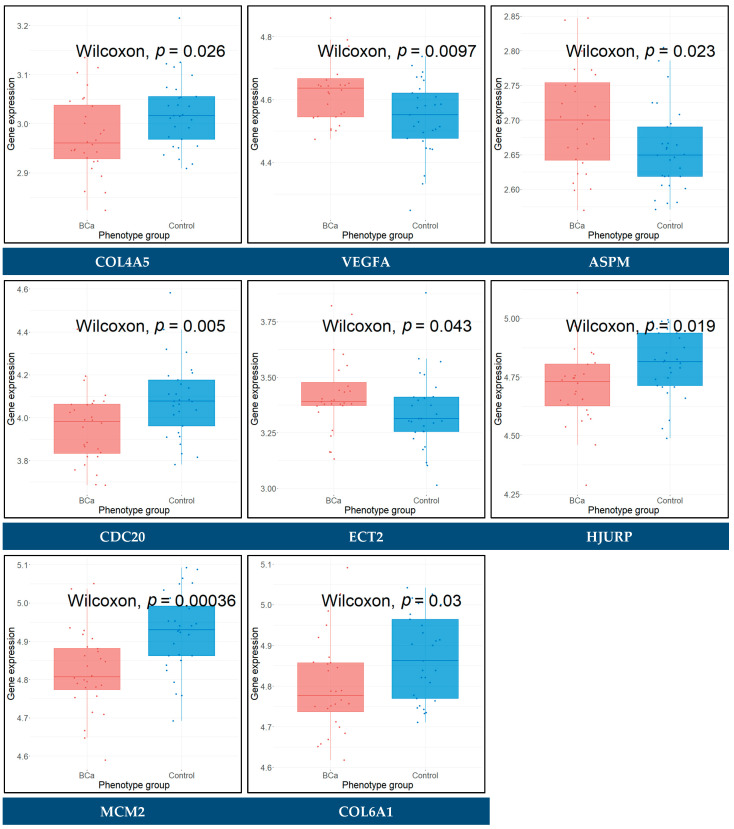
Significantly differentially expressed key hub genes in blood plasma samples using the Wilcoxon rank sum test in GSE138118 (*n* = 57).

The raw gene expression data of blood plasma samples, from series GSE138118, characterized by Affymetrix Human Gene 2.1 ST Array (GPL17692), were downloaded and appropriately pre-processed. The dataset consisting of 28 BCa and 29 control blood plasma samples was adjusted for batch effects. The Wilcoxon rank sum test was conducted to assess the statistical significance between the BCa and control groups for all the key hub genes. The hub genes with a *p*-value < 0.05 were considered as significantly differentially expressed between BCa and control blood samples. These genes included *ANXA5*, *CD34*, *CDT1*, *COL4A5*, *VEGFA*, *ASPM*, *CDC20*, *ECT2*, *HJURP*, *MCM2*, and *COL6A1*. The Wilcoxon rank sum test results for these genes are presented in boxplots in Figure 13.

### 3.10. Prognostic Genes for BCa

**Figure 14:**
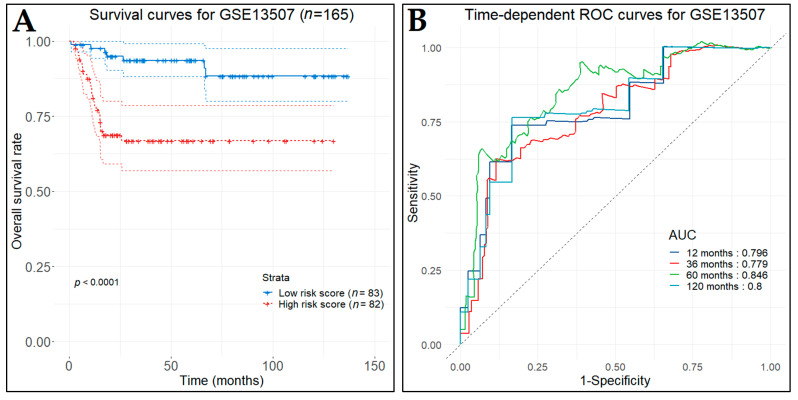

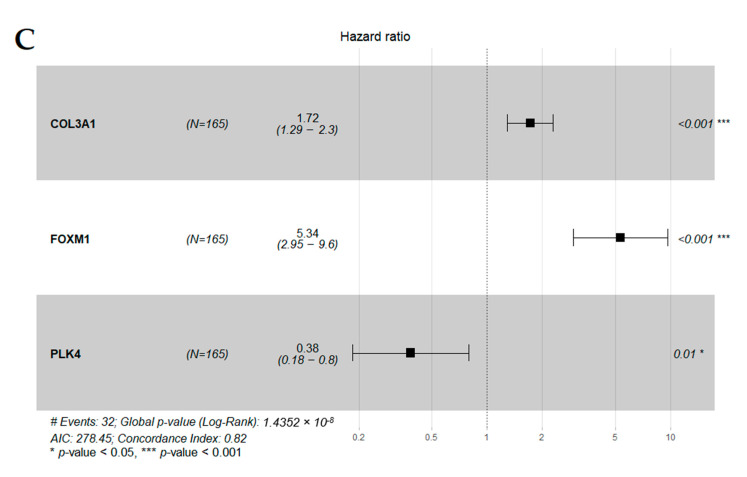
Survival analysis for the GSE13507 (*n* = 165) dataset (training set). (**A**) Kaplan–Meier curves for the overall survival of BCa patients as stratified by the three-gene prognostic index. Patients were divided into low- and high-risk groups according to the median prognostic index. (**B**) Time-dependent ROC curves (one-, three-, five-, and ten-year predictions) to assess the prognostic accuracy of the three-gene prognostic model. (**C**) Forest plot for the multivariate Cox regression analysis on the GSE13507. The figure incorporates the hazard ratio value (e^coef^) along with the 95% CI and *p*-value for each gene.

To better comprehend which of the 61 key hub genes were more closely associated with clinical outcomes of BCa patients, we further evaluated these genes by applying univariate Cox regression analysis on survival data of 165 patients with BCa of various stages (GSE13507). Univariate Cox regression analysis indicated that 46 genes were of statistically significant correlation with OS (Table 5). We performed LASSO Cox regression analysis with 10-fold cross validation to further decrease the number of significant genes and properly detect those that were highly associated with BCa-related survival. Nine genes, namely *ACTG2*, *ASPM*, *CDCA8*, *COL3A1*, *COL4A5*, *FOXM1*, *MKI67*, *PLK4*, and *SPP1*, were identified. Multivariate Cox regression analysis indicated that the expressions of *COL3A1*, *FOXM1*, and *PLK4* were highly and independently connected with the BCa patients’ prognosis (Table 5), and were used to calculate each gene’s coefficient. Finally, a three-gene signature prognostic model was constructed. We calculated a prognostic risk score for every patient of the training set (GSE13507) based on their distinct expression levels of the three genes, using the prognostic index (PI):

(4) $$PI=0.5405\cdot exp_{COL3A1}+1.6748\cdot exp_{FOXM1}-0.9583\cdot exp_{PLK4}$$

where exp is the expression value of the respective gene. The forest plot of our prognostic model is depicted in Figure 14C. The co-expression correlation analysis was performed through the GEPIA2 platform and indicated that no couple of the three genes held a Pearson correlation coefficient greater than 0.6 in BCa (Figure S8).

Calculating the prognostic index for each of the patients in the training set and grouping them based on the median value, the survival time for the high-risk (poor prognosis) patients (*n*  =  82) was significantly worse (*p*-value < 0.0001) than that of the low-risk (good prognosis) patients (n  =  83), as indicated by the Kaplan–Meier curves (Figure 14A). Additionally, the three-gene prognostic signature was assessed for its prognostic accuracy, conducting time-dependent ROC analysis at specific follow-up times; namely one, three, five, and ten years after diagnosis. The AUC at the different cut-off times were 0.796, 0.779, 0.846, and 0.8, respectively (Figure 14B).

**Figure 15:**
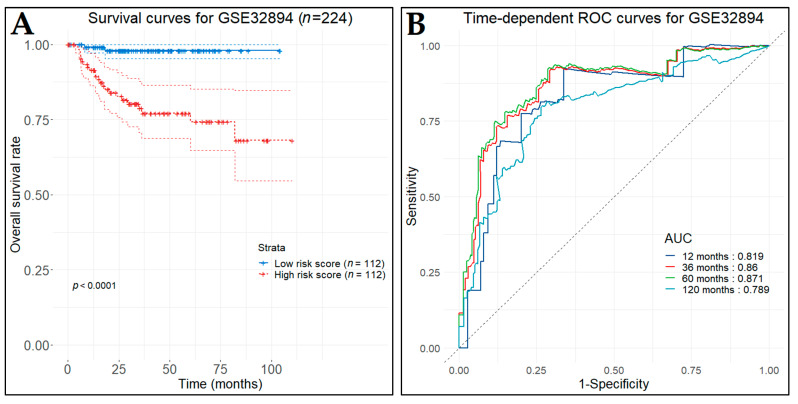
Survival analysis for the GSE32894 (*n* = 224) dataset (first test set). (**A**) Kaplan–Meier curves for overall survival of BCa patients as stratified by the three-gene prognostic index. Patients were divided into low- and high-risk groups according to the median prognostic index. (**B**) Time-dependent ROC curves (one-, three-, five-, and ten-year predictions) to assess the prognostic accuracy of the three-gene prognostic model.

**Figure 16:**
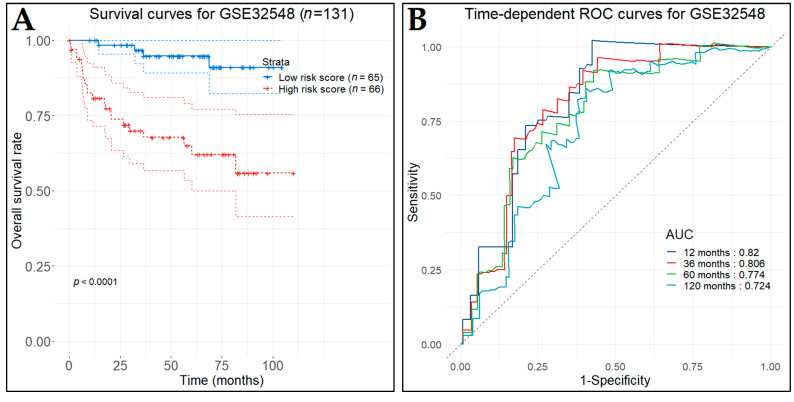
Survival analysis for the GSE32548 (*n* = 131) dataset (second test set). (**A**) Kaplan–Meier curves for overall survival of BCa patients as stratified by the three-gene prognostic index. Patients were divided into low- and high-risk groups according to the median prognostic index. (**B**) Time-dependent ROC curves (one-, three-, five-, and ten-year predictions) to assess the prognostic accuracy of the three-gene prognostic model.

In the first test set (GSE32894), the low-risk patient group (*n* = 112), as predicted by the prognostic model, demonstrated significantly longer OS (*p*-value < 0.0001) in contrast to the high-risk patient group (*n* = 112) (Figure 15A). The time-dependent ROC curves were plotted and the one-, three-, five-, and ten-year AUC values were 0.819, 0.86, 0.871, and 0.789, respectively (Figure 15B). In the second test set (GSE32548), the low-risk (*n* = 65) and the high-risk (*n* = 66) patient groups also produced significantly different OS times (*p*-value < 0.0001) (Figure 16A). Likewise, the time-dependent ROC curves were plotted and the corresponding AUC values were 0.82, 0.806, 0.774, and 0.724 (Figure 16B).

**Figure 17:**
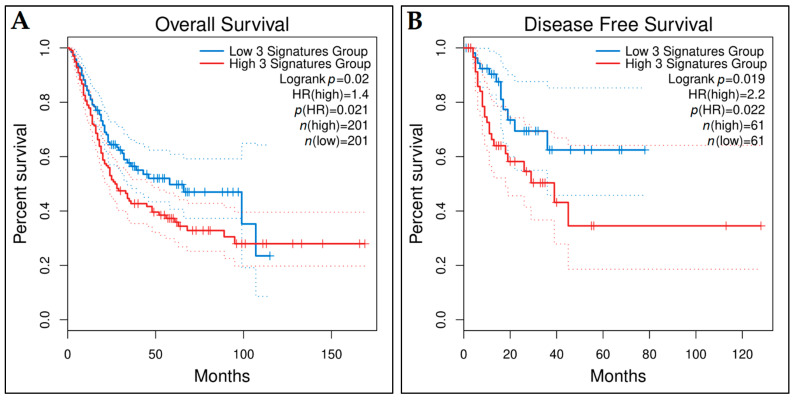
Kaplan–Meier survival plots of the three-gene prognostic signature, generated using the GEPIA2 platform. Red and blue lines indicate the high- and low-risk patient groups, respectively. Patients were grouped according to (**A**) median cut-off value for overall survival and (**B**) a custom cut-off high and low value of 85% and 15%, respectively, for disease-free survival.

Finally, we investigated whether the signature, constituted of *COL3A1*, *FOXM1*, and *PLK4* could be prognostic for the OS or DFS of TCGA-BCa patients. For this purpose, we tested the three-gene signature with Kaplan–Meier survival analysis using the GEPIA2 platform. The patients were separated into low- and high-risk groups (*n* = 201 in each group) based on the median expression value and the two groups showed statistically significant different OS time (*p*-value = 0.02) (Figure 17A). Regarding the DFS time, the lower 15% and the upper 85% of the sorted expression values were used for distinguishing the low- and high-expression patient groups, respectively (*n* = 61 in each group). The difference between the DFS curves of these expression groups was found to be significant (*p*-value = 0.019) (Figure 17B).

To conclude, the Kaplan–Meier survival analysis and the AUC values at the different cut-off times indicated that the three-gene signature model holds a very good prognostic accuracy regarding grouping BCa patients in terms of survival. These results endorsed the validation of this prognostic gene signature.

**Figure 18:**
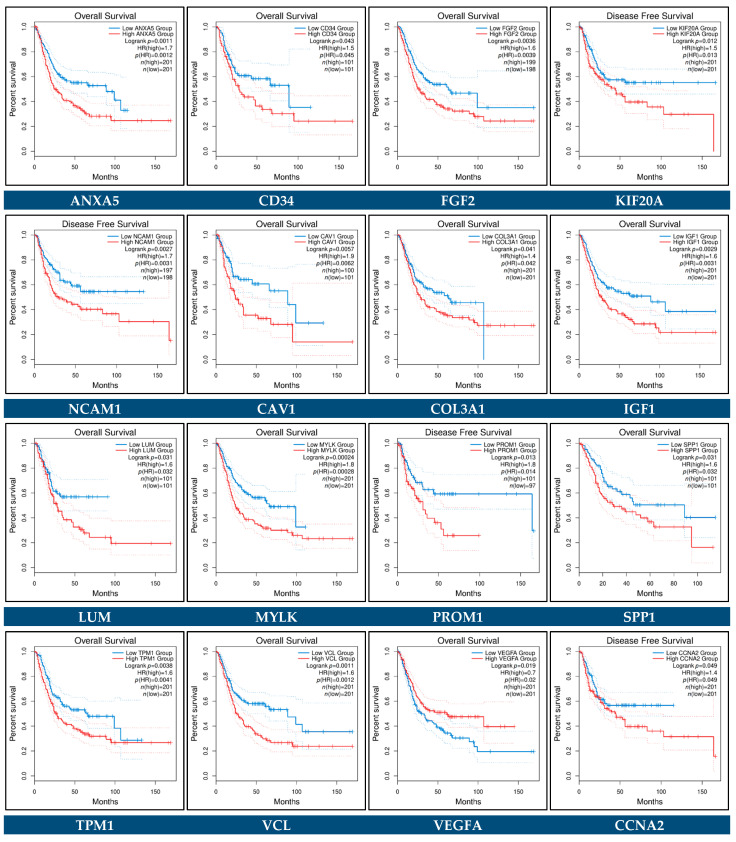

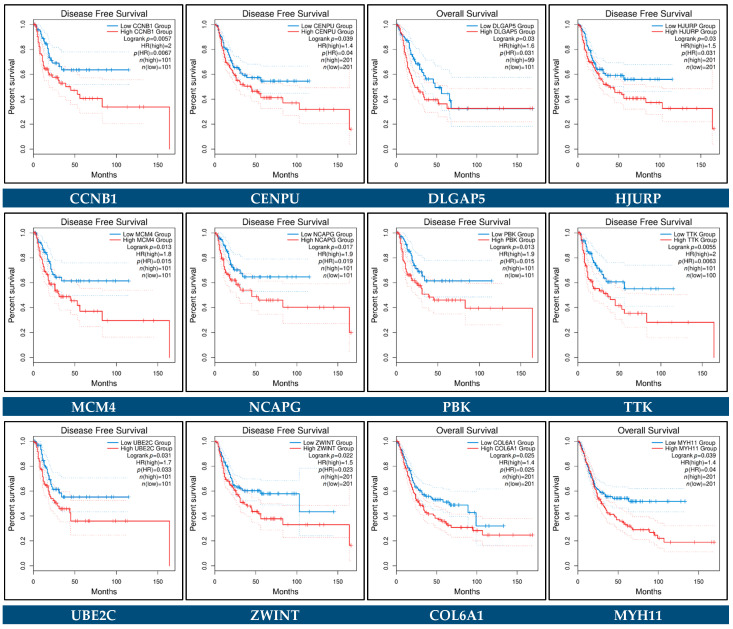
Kaplan–Meier survival plots of key hub genes, generated using the GEPIA2 platform. Red and blue lines indicate the high- and low-risk patient groups, respectively. Patients were grouped according to median or quartile cut-off values.

Survival plots for the individual key hub genes were generated by utilizing the GEPIA2 platform and were used to observe the OS and DFS status for each key hub gene in BCa (Figure 18). The OS and DFS plots compared high- and low-expression groups in Bca tissues and a *p*-value <  0.05 was regarded as statistically significant. Elevated expression levels of *ANXA5*, *CD34*, *FGF2*, *CAV1*, *COL3A1*, *IGF1*, *LUM*, *MYLK*, *SPP1*, *TPM1*, *VCL*, *DLGAP5*, *COL6A1*, and *MYH11* were found to be correlated with poorer patient OS, whereas expression levels of *VEGFA* were found to be inversely correlated with OS (Figure 18). Moreover, the high expression levels of *KIF20A*, *NCAM1*, *PROM1*, *CCNA2*, *CCNB1*, *CENPU*, *HJURP*, *MCM4*, *NCAPG*, *PBK*, *TTK*, *UBE2C*, and *ZWINT* were found to be correlated with worse DFS. No significant relationship was observed for other hub genes (data not shown).

### 3.11. Predictive Genes for BCa

The raw gene expression data of MIBC samples, from series GSE169455, characterized by Affymetrix Human Gene 2.1 ST Array (GPL17692), were downloaded and appropriately pre-processed. The dataset consists of 149 MIBC patients receiving preoperative cisplatin-based chemotherapy and was adjusted for batch effects. The Wilcoxon rank sum test was applied to assess each key hug gene’s statistical significance between the “No response”, the “Partial response”, and the “Complete response” groups. In addition, statistical significance between the “No response” and “Partial/Complete response” groups was also investigated for all the key hub genes.

**Figure 19:**
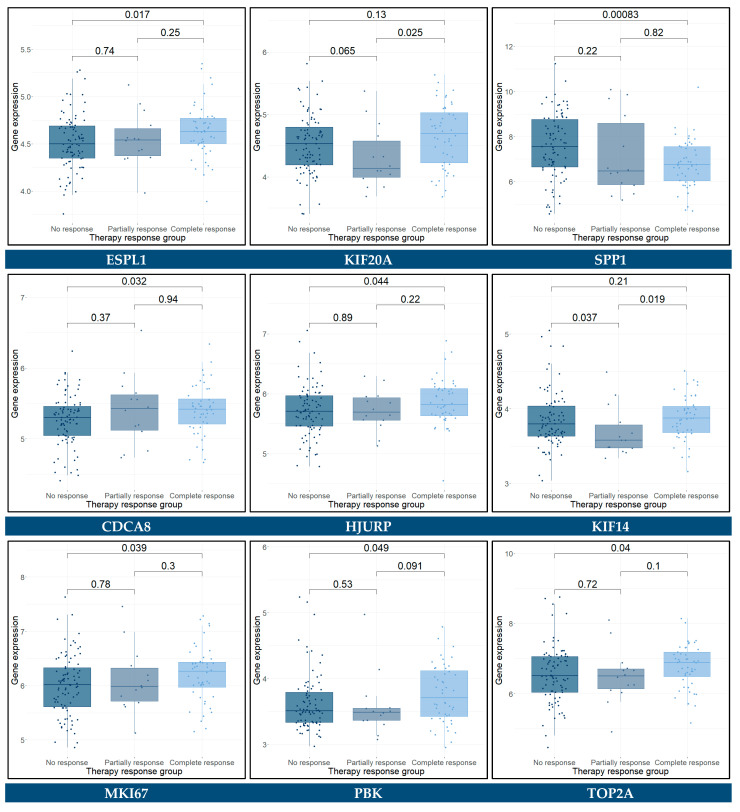

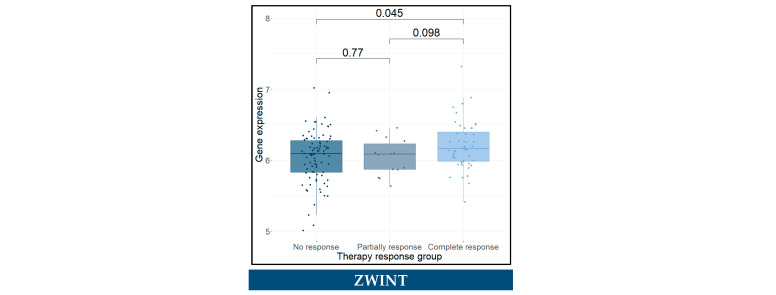
Significantly differentially expressed key hub genes between “No response”, “Partially response”, and “Complete response” to preoperative cisplatin-based chemotherapy groups of MIBC patients.

**Figure 20:**
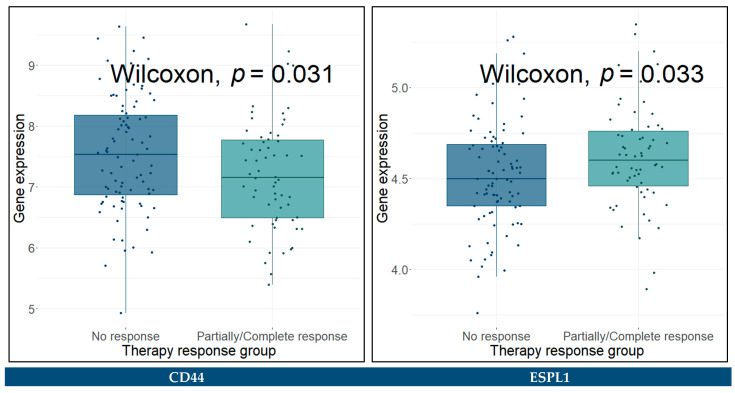

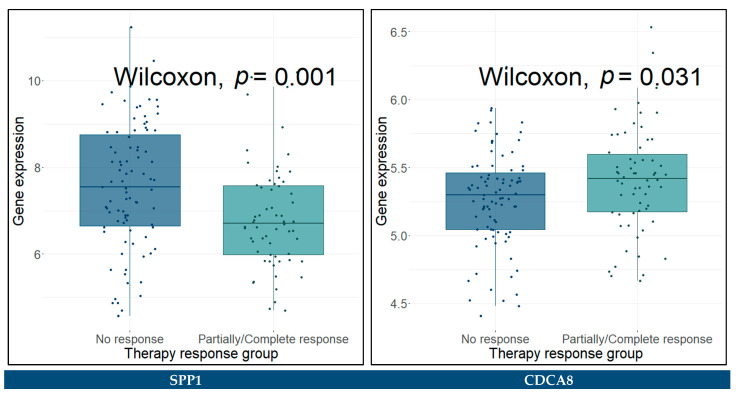
Significantly differentially expressed key hub genes between “No response” and “Partially/Complete response” for preoperative cisplatin-based chemotherapy groups of MIBC patients.

The hub genes with a *p*-value < 0.05 were identified as significantly differentially expressed between the groups. These genes included *ESPL1*, *SPP1*, *CDCA8*, *HJURP*, *MKI67*, *PBK*, *TOP2A*, and *ZWINT* between patients that did not respond to therapy and those who responded completely, *KIF14* between patients that did not respond to therapy and those who responded partially, and *KIF20A* and *KIF14* between the partially and completely responded patients. The Wilcoxon rank sum test results for these genes are presented in Figure 19. As regards the two-class comparison between the “No response” and “Partial/Complete response” groups, the genes *CD44*, *ESPL1*, *SPP1*, and *CDCA8* were significantly differentially expressed. The Wilcoxon rank sum test results for these genes are presented in Figure 20.

For the purpose of investigating the role of the key hub genes regarding patients’ response to cisplatin-based chemotherapy, we additionally assessed these genes using univariate Cox regression analysis in a total of 149 patients with MIBC (GSE169455). The clinical characteristics of patients for this dataset contain information about recurrence-free survival (RFS), cancer-specific survival (CSS), and overall survival (OS) events. Therefore, we conducted the univariate Cox regression analysis for each one of these events. Only two genes, *SPP1* and *CDCA8*, were found to be statistically significant with RFS, CSS, and OS time simultaneously, with *SPP1* showing a strong statistical significance (Table 6). Additionally, we performed LASSO Cox regression analysis with 10-fold cross validation to select which of the key hub genes were highly associated with BCa RFS. A total of 19 genes, namely *ACTG2*, *ANXA5*, *AURKB*, *CCNA2*, *CCNB2*, *CD44*, *CDC45*, *CDCA8*, *CDT1*, *CENPA*, *COL6A1*, *DLGAP5*, *IGF1*, *KIF14*, *NCAM1*, *NEK2*, *SPP1*, *VCL*, and *ZWINT*, were identified. Multivariate Cox regression analysis indicated that the expression of *ANXA5*, *CD44*, *NCAM1*, *SPP1*, *CDCA8*, and *KIF14* were highly and independently associated with the RFS of BCa patients (Table 6) and were used to calculate the coefficient of each gene. Finally, a six-gene signature predictive model was constructed, and the predictive index (PDI) formula for this model was:

(5) $$\begin{array}{ll} PDI= & |-0.87492\cdot exp_{ANXA5}+0.50317\cdot exp_{CD44}+0.46781\cdot exp_{NCAM1}\\ & +0.54406\cdot exp_{SPP1}-1.70391\cdot exp_{CDCA8}+1.54315\cdot exp_{KIF14}| \end{array}$$

**Figure 21:**
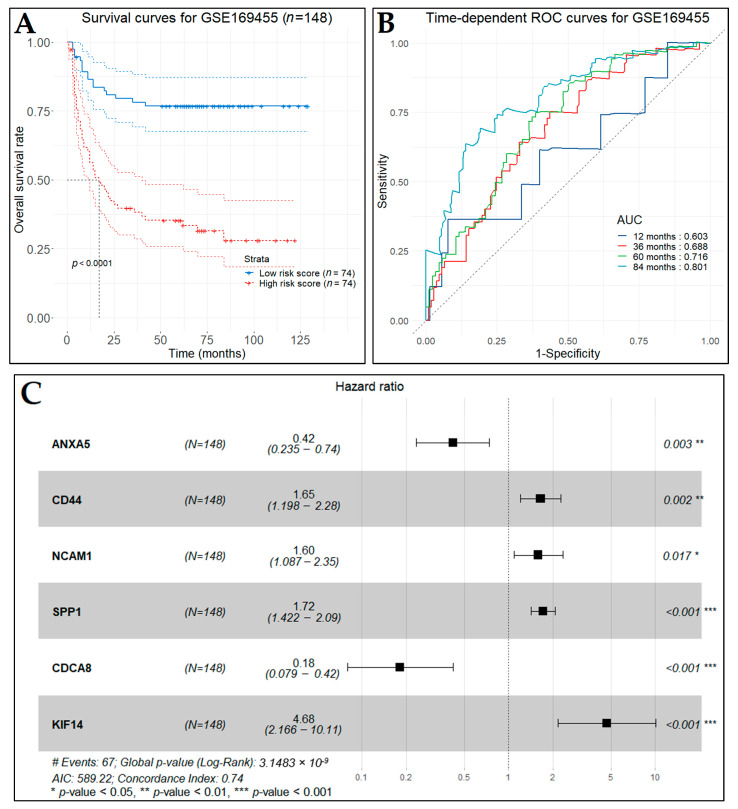
Survival analysis for the GSE169455 (*n* = 148) dataset (training set). (**A**) Kaplan–Meier curves for disease-free survival of MIBC patients who received cisplatin-based chemotherapy as stratified by the six-gene predictive index. Patients were divided into low- and high-risk groups according to the median predictive index. (**B**) Time-dependent ROC curves (one-, three-, five-, and 10-year predictions) to assess the predictive accuracy of the six-gene predictive model. (**C**) Forest plot for multivariate Cox regression analysis on the GSE169455. The figure incorporates the hazard ratio value (e^coef^) along with the 95% CI and *p*-value for each gene.

The forest plot of our predictive model is presented in Figure 21C. The co-expression correlation analysis for the six genes was performed through the GEPIA2 platform and indicated that no couple of these genes held a Pearson correlation coefficient greater than 0.44 in BCa (Figure S9).

Applying the predictive index for each of the patients in the training set, the recurrence-free survival time for the high-risk patients (*n*  =  74) was significantly worse (*p*-value < 0.0001) than that of the low-risk patients (*n*  =  74), as indicated by the Kaplan–Meier curves (Figure 21A). In addition, we assessed the predictive performance of the six-gene signature using time-dependent ROC analysis at specific follow-up times, namely one, three, five, and ten years after diagnosis. The AUC at the different cut-off times were 0.603, 0.688, 0.716, and 0.801, respectively (Figure 21B).

**Figure 22:**
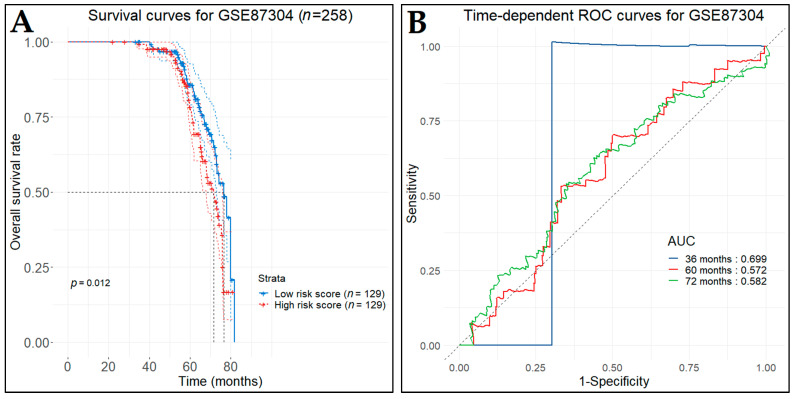
Survival analysis using the GSE87304 (*n* = 258) dataset (first test set). (**A**) Kaplan–Meier curves for disease-free survival of MIBC patients who received cisplatin-based chemotherapy as stratified by the six-gene predictive index. Patients were divided into low- and high-risk groups according to the median predictive index. (**B**) Time-dependent ROC curves (three-, five-, and six-year predictions) to assess the predictive accuracy of the six-gene predictive model.

**Figure 23:**
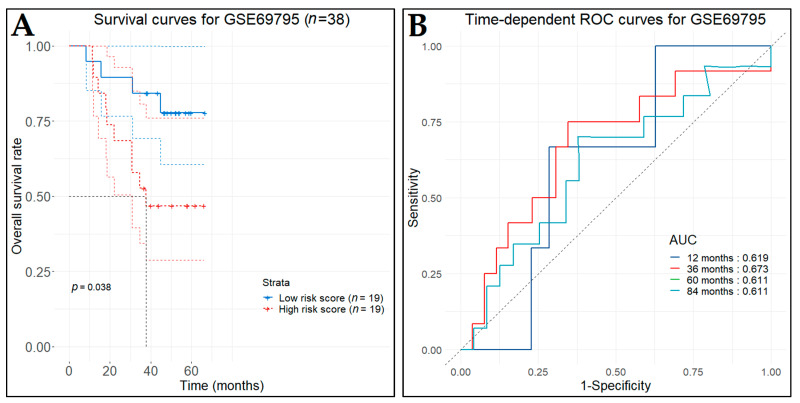
Survival analysis using the GSE69795 (*n* = 38) dataset (second test set). (**A**) Kaplan–Meier curves for disease-free survival of MIBC patients who received cisplatin-based chemotherapy as stratified by the six-gene predictive index. Patients were divided into low- and high-risk groups according to the median predictive index. (**B**) Time-dependent ROC curves (one-, three-, five-, and seven-year predictions) to assess the predictive accuracy of the six-gene predictive model.

In the first test set (GSE87304), the survival data were available for 258 out of 305 patients. Thus, the low-risk patient group (*n* = 129), as indicated by the predictive model, demonstrated statistically significant longer DFS (*p*-value < 0.012) in comparison with the high-risk patient group (*n*  =  129) (Figure 22A). The time-dependent ROC curves were drawn and the three-, five-, and six-year AUC values were 0.699, 0.572, and 0.582, respectively (Figure 22B). In the second test set (GSE69795), the low-risk patient group (*n* = 19), as indicated by the predictive model, demonstrated statistically significant longer DFS (*p*-value < 0.038) in comparison with the high-risk patient group (*n*  =  19) (Figure 23A). The time-dependent ROC curves were drawn and the one-, three-, five-, and seven-year AUC values were 0.619, 0.673, 0.611, and 0.611, respectively (Figure 23B).

**Figure 24:**
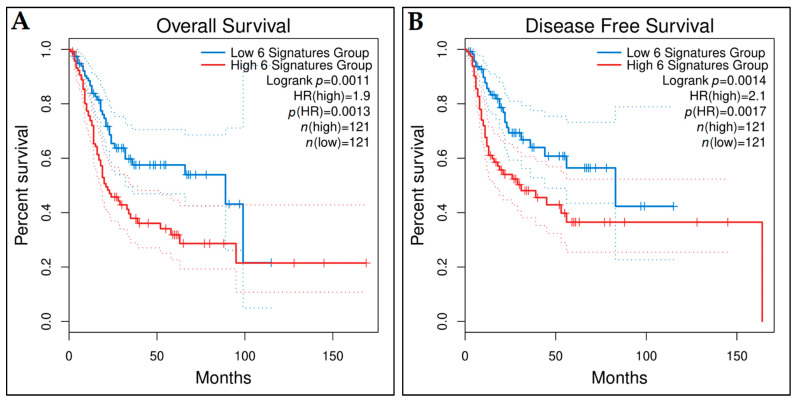
Kaplan–Meier survival plots of the six-gene predictive signature, generated using the GEPIA2 platform. Red and blue lines indicate the high- and low-expression patient groups, respectively. Patients were grouped (*n* = 121 in each group) according to custom low and high cut-off values of 30% and 70%, respectively, for comparing their (**A**) OS and (**B**) DFS.

Then, we investigated whether the six-gene signature constituted of *ANXA5*, *CD44*, *NCAM1*, *SPP1*, *CDCA8*, and *KIF14* could be prognostic for the OS or DFS of TCGA-BCa patients and tested this signature with Kaplan–Meier survival analysis using the GEPIA2 platform. The patients were separated into the low- and high-risk groups (*n* = 121 in each group) based on custom low- and high-cut-off values of 30% and 70%, respectively. The two groups showed statistically significant different OS time (*p*-value = 0.0011) and DFS time (*p*-value = 0.0014) (Figure 24). This indicated that the six-gene signature held also a prognostic value in OS and DFS of BCa patients.

In brief, the Kaplan–Meier survival analysis and the AUC values at the different cut-off times indicated that the six-gene signature model holds a quite good prognostic accuracy regarding the DFS time of MIBC patients who received preoperative cisplatin-based chemotherapy and, thus, it could be further assessed for whether it may predict the MIBC patients’ response to the preoperative chemotherapy treatment.

### 3.12. Expression Validation of Key Biomarkers and Immunohistochemistry

The key hub genes which were revealed to be differentially expressed between the BCa and control samples in either the urine or the blood plasma of Bca patients (*p*-value < 0.05), and concurrently hold a prognostic or predictive value, were considered as the key biomarker genes of our integrative meta-analysis. These key biomarker genes include *ANXA5*, *CDT1*, *COL3A1*, *SPP1*, *VEGFA*, *CDCA8*, *HJURP, TOP2A*, and *COL6A1*.

**Figure 25:**
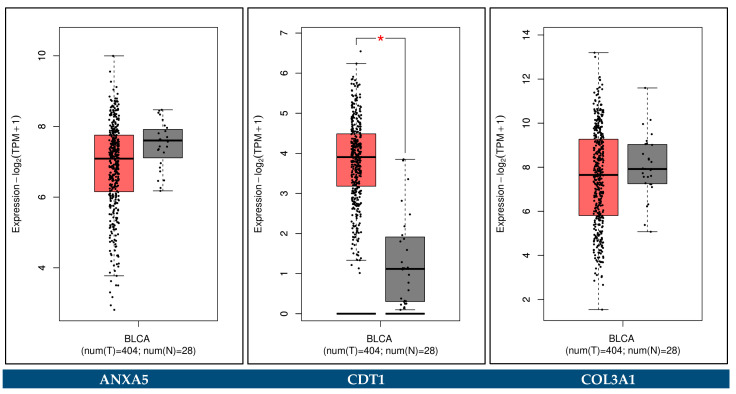

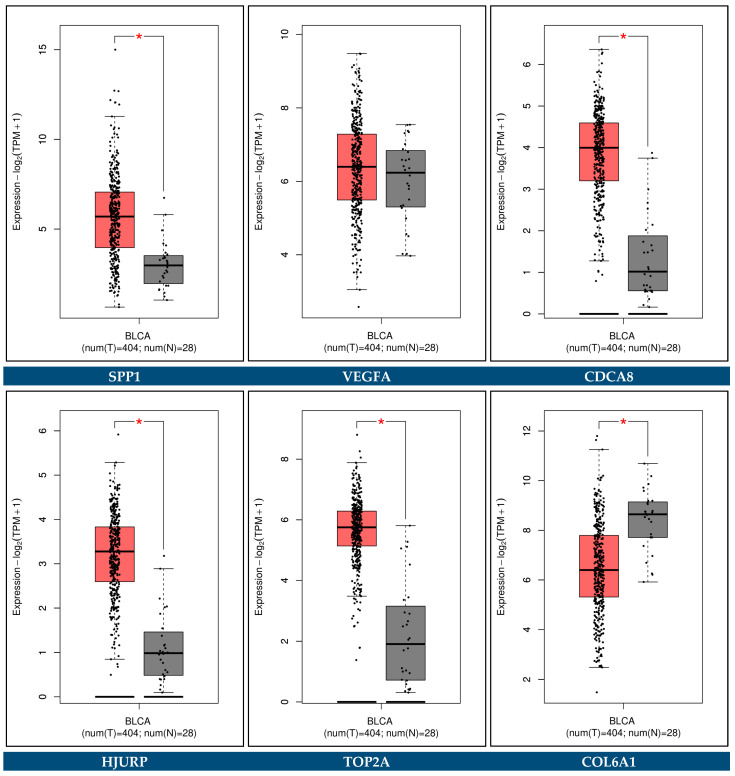
The gene expression level analysis of the nine key biomarkers in BCa patients, generated using the GEPIA2 platform. The red boxes represent the mRNA expression levels in BCa tissues and the gray boxes represent the expression levels in control bladder tissues from patients of the TCGA-BCa and GTEx cohorts. * indicates statistical significance applying *p*-value < 0.05 and |log_2_FC| < 1 as cut-off criteria (BLCA: bladder cancer, TPM: transcript count per million).

**Figure 26:**
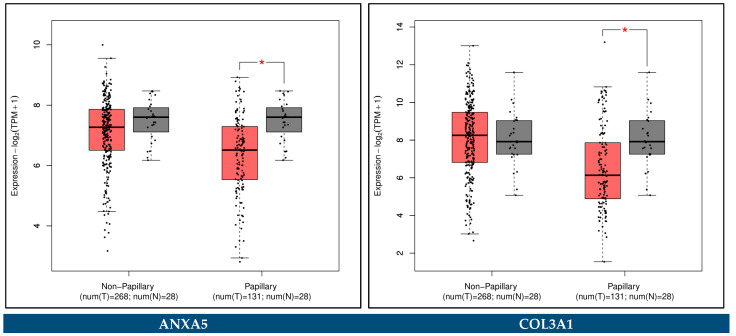
The gene expression level analysis of the *ANXA5* and *COL3A1* in BCa patients for non-papillary and papillary subtypes, generated using the GEPIA2 platform. The red boxes represent the mRNA expression levels in BCa subtype tissues and the gray boxes represent the expression levels in control bladder tissues from patients of the TCGA-BCa and GTEx cohorts. * indicates statistical significance applying *p*-value < 0.05 and |log_2_FC| < 1 as cut-off criteria (TPM: transcript count per million).

So that we would be able to confirm the altered mRNA expression levels of the proposed biomarker genes between Bca and normal groups, TCGA and GTEx datasets were analyzed using the GEPIA2 platform. The selected cut-off values were set as |log_2_FC| = 1 and *p*-value = 0.05. The corresponding boxplots were generated and downloaded from GEPIA2 (Figure 25). The plots demonstrated that the results of our differential expression analysis for all the genes were validated, in terms of the occurrence of down- or upregulation. However, for three genes, namely *ANXA5*, *COL3A1*, and *VEGFA*, the differences between the Bca and the control group means had a lower |log_2_FC| value than the selected one and, thus, they were not characterized as statistically significant. To allow us to explore the expression of these genes for the main Bca subtypes (i.e., non-papillary and papillary) in more detail, we further analyzed their expression and found that *ANXA5* and *COL3A1* were significantly differentially expressed in papillary subtype, using the aforementioned cut-off values (Figure 26).

**Figure 27:**
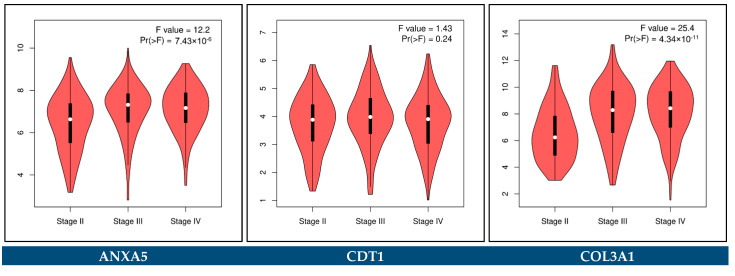

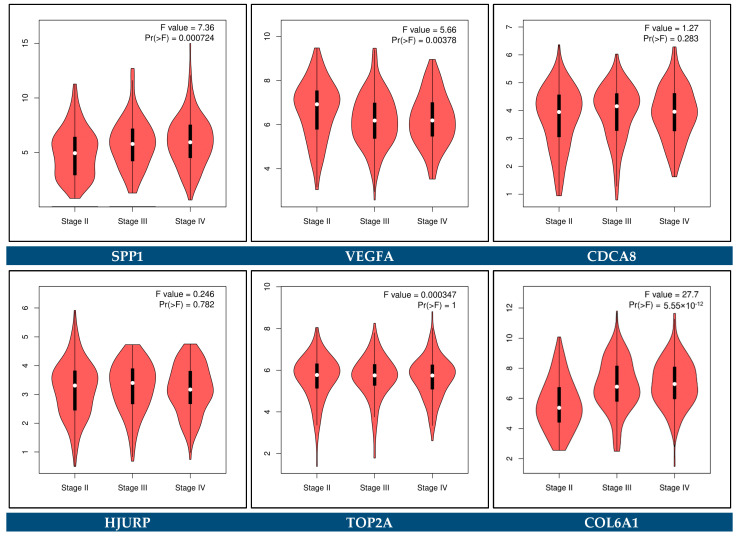
Violin plots representing the association between the expression levels of the nine key biomarker genes and the three pathological tumor stages among BCa patients, generated using the GEPIA2 platform and based on the TCGA-BLCA cohort. F value and Pr(>F) indicate the statistical value and the *p*-value of the F-test, respectively.

In an attempt to investigate the correlation of the key biomarker genes with the different pathological stages of BCa, we used the TCGA-BCa data and the corresponding feature from the GEPIA2 platform. The analysis showed that five out of the nine genes were strongly associated with the pathological BCa stages, highlighting their prognostic value for BCa. In particular, *ANXA5*, *COL3A1*, *SPP1*, *VEGFA*, and *COL6A1* were identified to be highly correlated with BCa stages, while no significant correlation was found for the others (Figure 27).

**Figure 28:**
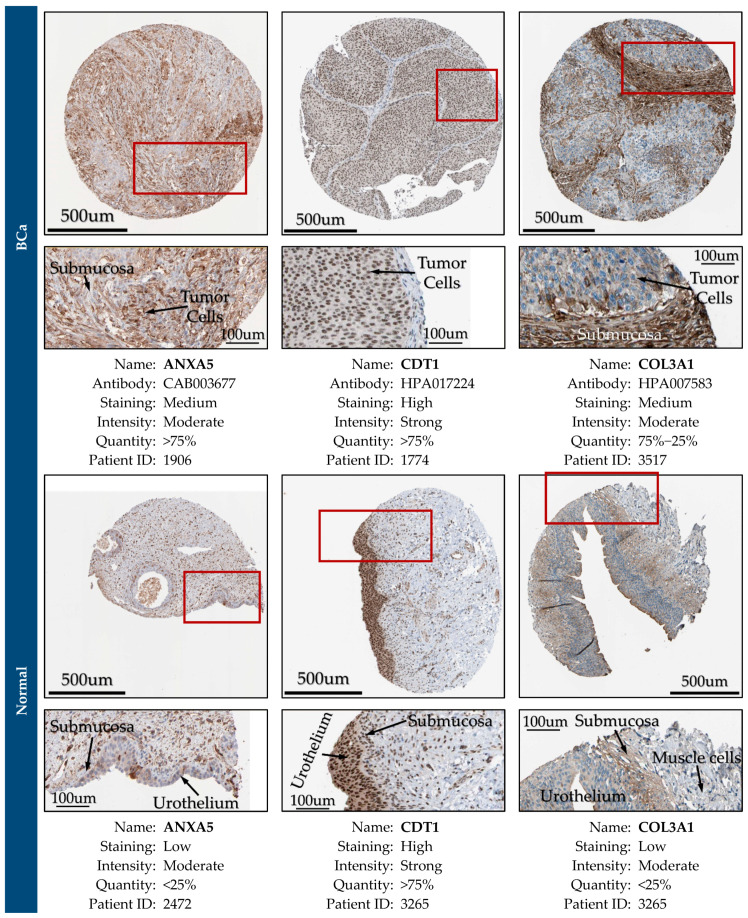

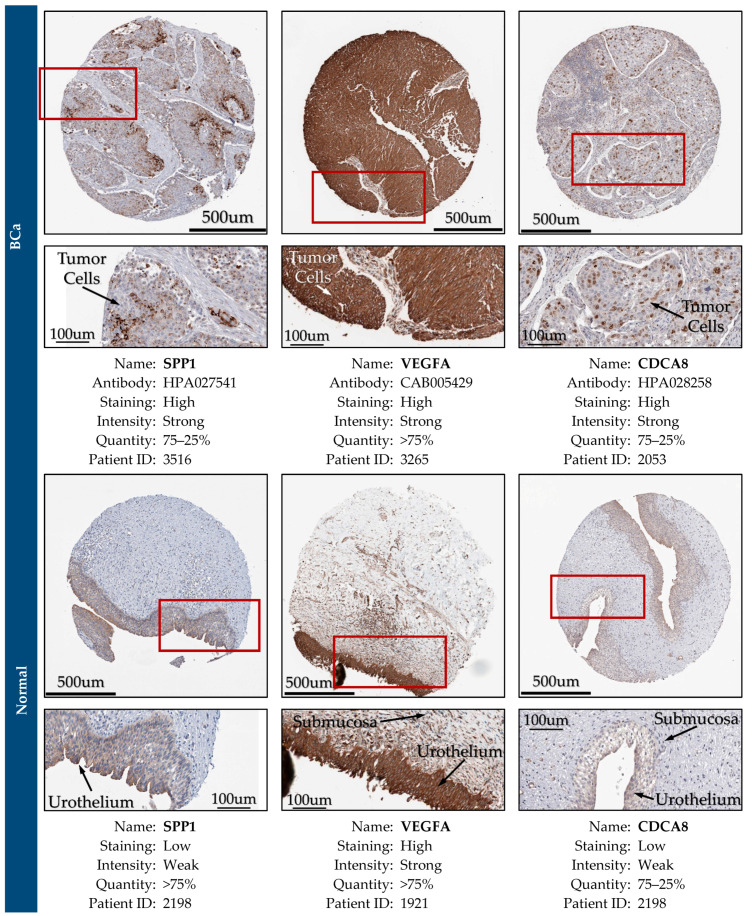

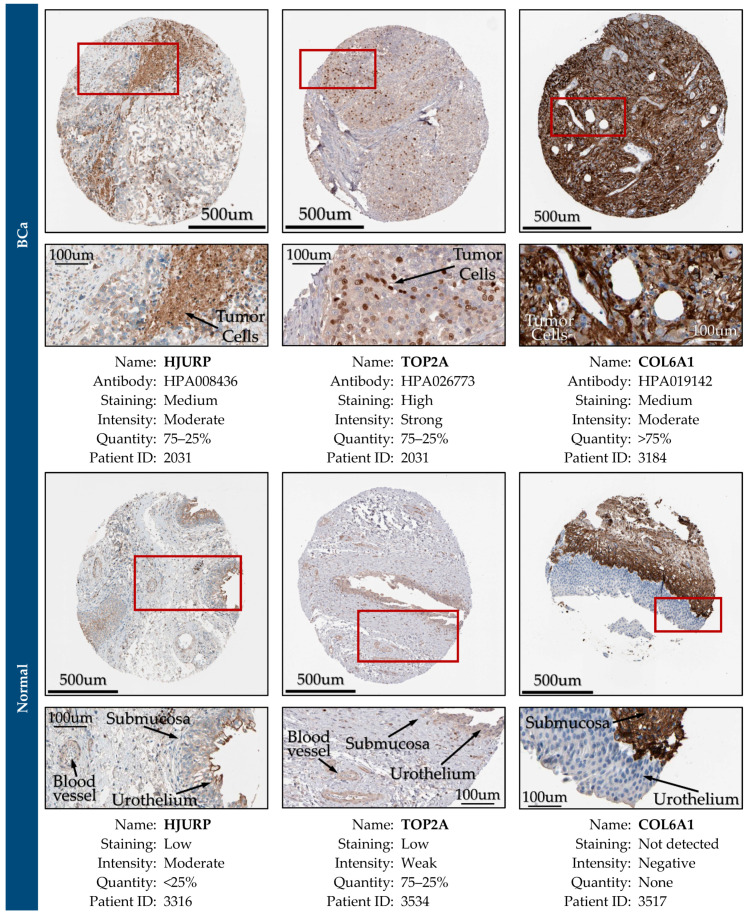
Immunohistochemical (IHC) validation of the nine key biomarker genes in cancer and normal human bladder tissue specimens, obtained from the Human Protein Atlas (available online: www.proteinatlas.org (accessed on 4 July 2022)). Staining demonstrated that protein expression of the nine key biomarker genes was higher in BCa tissues compared to normal bladder tissue samples.

The Human Protein Atlas (HPA) was utilized to obtain the protein expression levels which are encoded by the key biomarker genes in the urinary bladder tissue for both pathologic and normal states. The immunohistochemistry (IHC) analysis based on the HPA images revealed that *SPP1*, *CDCA8*, and *TOP2A* showed high antibody staining intensity in BCa tissues and low staining intensity in normal tissues. Further, *ANXA5*, *COL3A1*, and *HJURP* had medium staining intensity in cancerous tissues, whereas low intensity was inspected in normal bladder. *CDT1* and *VEGFA* showed high staining intensity in both BCa and normal bladder tissues. Lastly, the antibody intensity for *COL6A1* was higher in BCa tissue compared to the corresponding normal one, in which no staining was detected. The IHC analysis showcased that the expression levels of these proteins were generally upregulated in the protein expression level in BCa (Figure 28).

### 3.13. Diagnostic Performance of Key Biomarkers

To determine whether the identified key biomarker gene signature holds a diagnostic value, we used the genes as features and built various classification models utilizing all the datasets (with more than 10 samples) used in this integrative meta-analysis (Table 1), as well as the merged meta-dataset and an external set (ArrayExpress E-MTAB-1560). 

**Figure 29:**
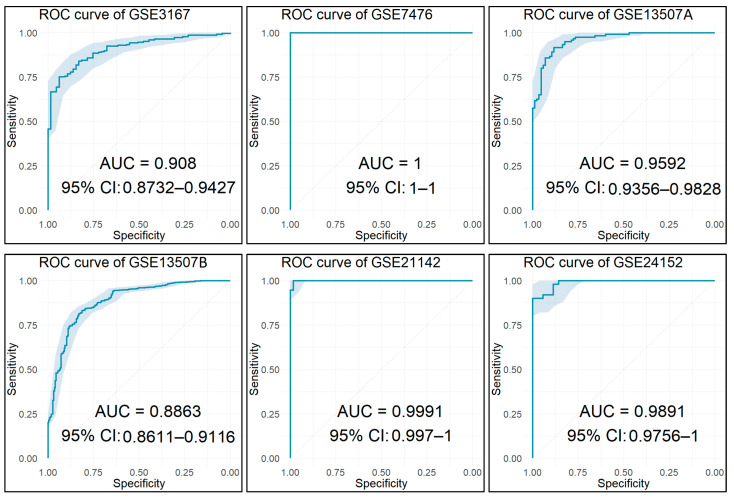

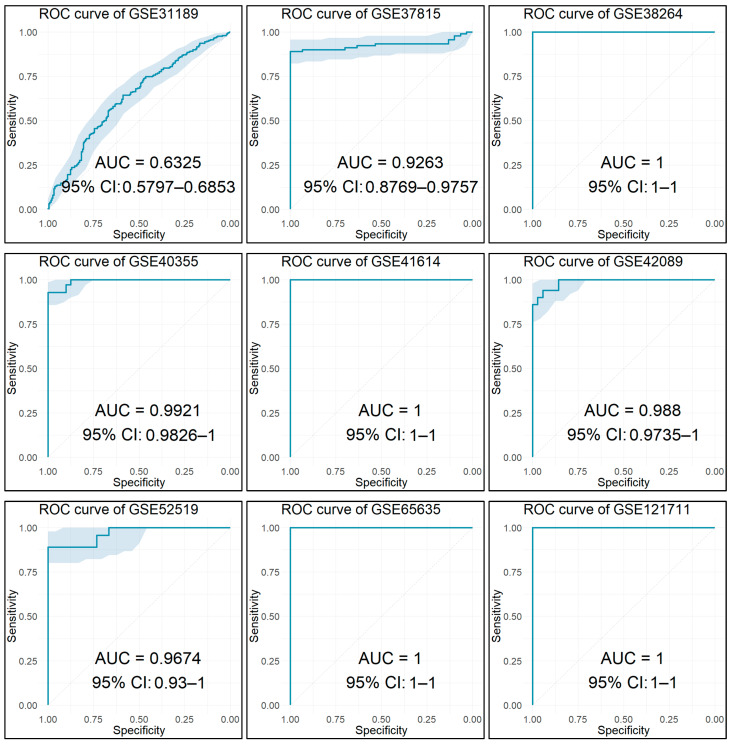
Validation of the diagnostic models, using the nine key biomarker genes as features, by ROC curve analysis on each of the datasets included in our integrative meta-analysis and contained at least 10 samples (see Table 1).

**Figure 30:**
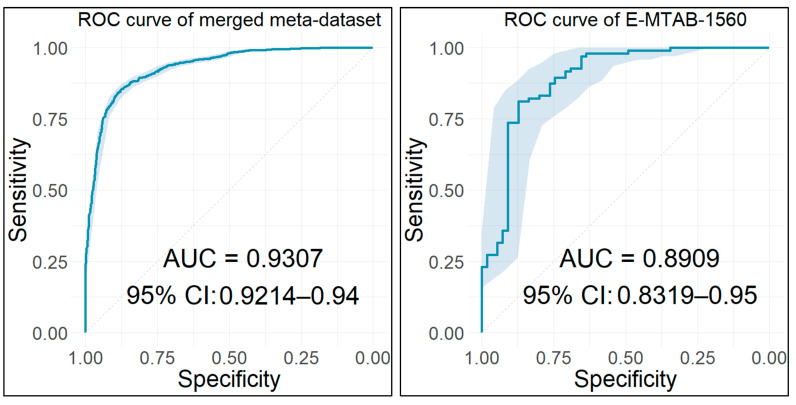
Validation of the diagnostic models, using the nine key biomarker genes as features, by ROC curve analysis on the merged meta-dataset (*n* = 606) and on external validation E-MAT-1650 dataset (*n* = 30).

For the individual datasets, a fivefold cross validation method was implemented, whereas for the final merged meta-dataset, a 10-fold cross validation was conducted and they were all repeated 10 times. The resulting ROC curves for all the built models in addition to their corresponding AUC values and the 95% CIs were plotted (Figure 29 and Figure 30). The results indicated a very high diagnostic performance of the various models, with the AUC values ranging from 0.8863 to 1.00 for the individual datasets, and reaching 0.9307 and 0.8909 for the merged meta-dataset and the external dataset, respectively. The classification model built from GSE31189 resulted in an AUC value of 0.6325, as this dataset suffered due to batch effects (as mentioned in Section 3.8), and was not considered in our overall evaluation.

## 4. Discussion

BCa is among the most common cancer types worldwide, accounting for high incidence, prevalence, mortality, as well as recurrence rate, and still remains an open clinical and social problem. Improved comprehension of its pathophysiology has evolved, but underlying molecular mechanisms and genetics need to be further elucidated. A major obstacle is the fact that its detection remains demanding due to the lack of specific and sensitive tumor markers, and the absence of new symptoms. This issue has become even more imperative during the COVID-19 era [97]. There is thus an urgent need to develop more efficient diagnostic, prognostic, and predictive markers in order to better manage and treat the onset and course of BCa.

In this study, we employed microarray data to investigate gene expression profiles in BCa. By combining and reanalyzing a high number of samples, we aimed to conclude more reliable results, statistical inferences, and gene expression signatures. In order to find a robust list of DEGs for BCa, we conducted a systematic review across multiple GEO studies using the PRISMA guidelines (Figure 2), selected the eligible datasets, which were downloaded from the GEO and pre-processed according to their microarray platform, controlled the quality of all samples and removed outliers, and created a common gene symbol set for all datasets. Finally, we developed a merged microarray meta-dataset, comprising 410 BCa and 196 healthy urinary bladder tissue samples, from 18 independent datasets, adopting an “early stage” integration approach [23]. Our comprehensive analysis, which is among the largest of its kind to the best of our knowledge, identified 815 DEGs between BCa and normal tissues.

The pathways significantly overrepresented in the DEGs list were investigated. The results from the GO analysis revealed biological processes related to the extracellular matrix, angiogenesis, muscle development, cell division, chromosome organization, and DNA replication, which are all fundamental processes for cancer development and progression (Figure 4) [98]. The KEGG pathway enrichment analysis exposed pathways enriched in PI3K-PKB/Akt signaling, microRNAs in cancer, cell cycle, focal adhesion, regulation of actin cytoskeleton, calcium signaling, proteoglycans in cancer, cellular senescence, vascular smooth muscle contraction, and bladder cancer, among others (Figure 5). The Reactome pathway analysis showed pathways enriched in the extracellular matrix organization, cell cycle checkpoints, Rho GTPase effectors, control of insulin-like growth factor (IGF) transport, DNA replication, platelet degranulation, and collagen degradation, to name a few (Figure 6). Finally, the enrichment analysis based on disease ontology exhibited urinary system cancer as the most significantly enriched term, followed by non-small cell lung carcinoma, as well as kidney, breast, musculoskeletal system, and renal and prostate cancers (Figure 7), indicating the high association of the identified DEGs with BCa and disclosing the shared mechanisms and commonalities of different types of cancer [99].

In our study, we combined the results from the PPI network analysis and WGCNA methods in order to identify the key hub genes for the occurrence and development of BCa (Table 4). The consensus WGCNA created a network established on the association among genes, as it is an unsupervised analysis, whereas the PPI network was created grounded on the know interactions among human proteins. The PPI analysis resulted in a densely connected protein network, which indicated high biological relevance (Figure 8). In the WGCNA, despite the meta-analysis of various and heterogeneous datasets, we resulted in highly correlated consensus key modules and phenotypic characteristics (Figure 10). It is noteworthy that the hub genes contained in the brown module were found to have a strong association with OS and DFS of BCa patients, whereas genes of the black module had relevance with the DFS of patients. The combination of the identified hub genes by these two methods resulted in 61 common genes characterized as key hub genes for our analysis.

A crucial current issue is the capability to detect BCa easily and early using less invasive methods and, ideally, with markers showing high sensitivity and specificity [100]. Molecular markers, such as circulating mRNAs, in urine and blood could offer promising sources to gain comprehension of BCa and its associated micro- and macro-environment. Therefore, we tested whether each of the key hub genes was differentially expressed in the urine or blood plasma of BCa patients. In urine specimens, 17 genes, namely *AURKB*, *CCNB2*, *CDC45*, *CDCA8*, *CDT1*, *CENPU*, *COL3A1*, *GINS2*, *KIF20A*, *MCM4*, *PBK*, *PLK4*, *SDC1*, *SPP1*, *TOP2A*, *TTK*, and *UBE2C*, showed statistically significant differential expression between BCa and healthy individuals. In previous studies, osteopontin (*SPP1*) was investigated in the urine of nephrolithiasis [101], Alzheimer’s disease patients [102], and in cancer patients presenting cisplatin-induced nephrotoxicity [103], and was found to provide diagnostic value. Syndecan one (*SDC1*) was measured by a multiplex immunoassay along with nine other protein biomarkers for the diagnosis of BCa [104] and for the detection of recurrent BCa [105]. Ubiquitin-conjugating enzyme E2 C (*UBE2C*) was analyzed in urine samples as a potential diagnostic marker for BCa [106]. Additionally, urinary peptidome profiling, using a 22-marker panel including collagen type III alpha one chain (*COL3A1*), was investigated for clinical diagnostics of preeclampsia [107]. Finally, minichromosome maintenance five (*MCM5*), a protein in the same family as *MCM4*, was measured in urine specimens using an immunofluorometric assay in order to diagnose genitourinary tract cancer [108]. Apart from the above proteins, literature on the rest of the urinary biomarkers is extremely limited, if any. Hence, these urine targets should be investigated for their potential diagnostic value in BCa patients.

As regards blood plasma specimens, 11 genes, namely *ANXA5*, *ASPM*, *CD34*, *CDC20*, *CDT1*, *COL4A5*, *COL6A1*, *ECT2*, *HJURP*, *MCM2*, and *VEGFA*, presented with statistically significant differential expression between BCa and healthy individuals. In previous findings, Annexin A5 (*ANXA5*) plasma levels were investigated as a potential biomarker for asthma diagnosis [109], pregnant and non-pregnant subjects [110], as well as for liver cirrhosis and hepatocellular carcinoma [111]. Abnormal spindle protein homolog (*ASPM*) was detected in circulating tumor cells through single-cell genomic characterization in cancer patients [112]. *CD34* serves as an essential marker in disease research, as it is routinely used for identifying and isolating human hematopoietic stem/progenitor cells applied in bone marrow transplantation. Due to its high sensitivity regarding endothelial cell differentiation, it has also been studied as a marker for cancer [113]. What is more, cell-free mRNAs of Holliday junction recognition protein (*HJURP*) were found to be expressed at significant levels in plasma from patients with lung cancer [114]. Vascular endothelial growth factor A (*VEGFA*) protein plays a significant role in the growth of blood vessels and, as such, in diseases that involve them. These diseases include heart disease [115], COVID-19 [116], and various types of cancer [117], like ovarian [118] and breast cancer [119]. Conclusively, it appears that the value of most of these potential blood molecular biomarkers remains unclear for BCa and their further examination may offer opportunities to improve understanding of BCa and assist its early identification, patient stratification, and enhanced outcome predictions.

In order to assess the prognostic value of the key hub genes, we conducted univariate Cox, LASSO, and multivariate Cox regression analyses. Based on the genes and coefficients resulting from the multivariate Cox regression analysis, we built a three-gene prognostic model for BCa patients, constituting *COL3A1*, *FOXM1*, and *PLK4*. Expression changes of *COL3A1* were found to be prognostic markers in BCa [120] and to be involved in the development of MIBC [121]. This gene was determined as a potential key biomarker gene for BCa in our study and is further analyzed below. Forkhead box protein M1 (*FOXM1*) was reported to participate in an axis that regulates the cell cycle process and promotes progression of BCa [122] and to be a strong prognostic marker for disease progression in NMIBC [123,124]. It was also found to play a role in BCa recurrence and drug resistance to cancer therapies [125]. Polo-like kinase four (*PLK4*) was characterized as an important regulator of BCa cell proliferation, and, therefore, as a potential novel molecular target for BCa treatment [126]. 

Our prognostic model achieved AUC values of time-dependent ROC curves for the 1/3/5 years of 0.796/0.779/0.846, 0.819/0.86/0.871, and 0.82/0.806/0.774 for the training set (GSE13507), the first test set (GSE32894) and the second test set (GSE32548), respectively. In the same context, L. Yang et al. proposed a nine-gene prognostic model to enhance the prognosis prediction of BCa, achieving an AUC value of 0.76 at five years on the training set and a value of 0.63 for the same time on the test set [127]. In another study, Z. Xie et al. suggested a 10-inflammatory response-associated gene prognostic model which reached AUC values of 0.71 and 0.67 at year one in the TCGA-BCa and GSE13507 cohorts, respectively [128]. Furthermore, J. Lin et al. constructed an 11-gene prognostic model for predicting overall survival in BCa patients, reaching 1/3/5-year AUC values of 0.686/0.665/0.666, 0.800/0.742/0.697, 0.826/0.792/0.763, and 0.781/0.831/0.839 for the TCGA-BCa, GSE13507, GSE32548, and GSE32894 cohorts, respectively [129]. F. Tang et al. developed a seven-gene signature in order to predict the BCa patient prognosis, succeeding AUC values of 0.711/0.714/0.711 and 0.608/0.680/0.638 for the years one, three, and five in the training and test sets, respectively [130]. Additionally, C. Zhou suggested an 11-autophagy-related gene signature to predict the prognosis of BCa patients, showing a predictive efficiency for 1/3/5-year of 0.702/0.744/0.794 and 0.695/0.640/0.658 in the training and validation cohorts, respectively [131]. Moreover, F. Xu developed a six-gene prognostic signature for BCa, showing AUC values for cancer-specific survival of 3/5 years of 0.96/0.967, 0.744/0.748, and 0.576/0.606 for the training set (GSE32894) and the test sets (GSE13507 and TCGA-BCa), respectively [132]. Finally, F. Chen et al. constructed an eight-gene prognostic prediction model for BCa, which achieved maximum AUC values of 0.795 and 0.669 for the TCGA-BCa training and test sets, respectively [133]. Remarkably, this mini review of recent studies underlines the superior performance of our simple three-gene prognostic model and emphasizes its validity.

For the purpose of evaluating the predictive value of the key hub genes in terms of therapy response, we performed univariate Cox, LASSO, and multivariate Cox regression analyses on disease-free survival data of MIBC patients who received cisplatin-based chemotherapy treatment. Based on the genes and coefficients resulting from multivariate Cox regression analysis, we built a six-gene predictive model for MIBC BCa patients, constituting *ANXA5*, *CD44*, *NCAM1*, *SPP1*, *CDCA8*, and *KIF14*. *ANXA5*, *SSP1*, and *CDCA8* were characterized as potential key biomarker genes by our analysis, and their function, as well as connection with Bca, are described below. The cluster of differentiation 44 (*CD44*) antigen expression levels were reported to be associated with progression, metastasis, and disease failure of BCa [134]. Additionally, *CD44* expression was associated with BCa tumor aggressiveness [135], and it was related to the prediction of the radiation response of BCa cells [136]. Its expression was also suggested to be useful for prognostication and treatment options in primary BCa [137]. Neural cell adhesion molecule one (*NCAM1*) has not been extensively investigated in BCa, but there are studies that link its expression with drug resistance in acute myeloid leukemia [138], pleuropulmonary blastoma [139], and cervical intraepithelial neoplasia [140]. Kinesin family member 14 (*KIF14*) expression levels were linked to chemosensitivity of hepatocellular carcinoma [141] and cervical cancer [142], as well as to prognosis of various cancers, such as breast [143] and pancreatic cancer [144].

Our predictive model achieved AUC values of time-dependent ROC curves for the first and third years of 0.603/0.688, 0.699/0.572, and 0.619/0.673 for the training set (GSE169455), the first test set (GSE87304), and the second test set (GSE69795), respectively. The literature on gene signature models for predicting MIBC patients’ response to preoperative therapy is limited. In a similar study, W. Jiang et al. developed an immune-relevant nine-gene signature that could predict the immunotherapeutic response of immune checkpoint inhibitors, achieving a maximum AUC value of 0.69 and 0.64, in TCGA-BCa and IMvigor210 cohorts, respectively [145]. C. Shen et al. constructed an immune-associated two-gene signature to predict MIBC patients’ response to immunotherapy, succeeding with an AUC value of 0.695 in terms of its predictive ability [146]. S. J. Choi et al. developed a radiomic-based model for predicting the response of MIBC patients to neoadjuvant chemotherapy (NAC), achieving an AUC value of 0.75 for the validation set [147]. In a similar line, A. Parmar et al. used a predictive radiomic signature for MIBC patients’ response to NAC, reaching an AUC value of 0.63 in terms of discriminating the patients into responders and non-responders [148]. These findings indicate that our model’s predictive performance is satisfactory. To date, efforts to predict tumor response to NAC are still ongoing and mRNA-based gene expression profiling markers that can accurately predict response have yet to be introduced [149].

Taking into account the results of our integrative meta-analysis regarding the key hub genes that were identified to be differentially expressed in urine or blood plasma of BCa patients and concurrently hold a prognostic or predictive value, we concluded with some potential key biomarker genes regarding BCa. These genes include *ANXA5*, *CDT1*, *COL3A1*, *SPP1*, *VEGFA*, *CDCA8*, *HJURP*, *TOP2A*, and *COL6A1*.

Annexin A5 (*ANXA5*) is a protein kinase C inhibitor and one of the twelve annexins that have been identified in humans (*ANXA1-11*, *13*). It constitutes an anticoagulant protein that indirectly inhibits the thromboplastin-specific complex that participates in the coagulation cascade. In general, annexins are involved in the homeostatic regulation of intracellular calcium ion concentration and play a significant role in the cell life cycle, cell signaling, inflammation, growth, differentiation, exocytosis, and apoptosis. The annexins are normally found inside human cells. However, some annexins (*ANXA1*, *ANXA2*, and *ANXA5*) can be secreted from the cytoplasm to outside cellular environments, such as blood. In our study, the expression of *ANXA5* was significantly overexpressed in the blood plasma of BCa patients, whereas it was significantly under-expressed in the urinary bladder tissue of BCa patients. This is owed to the fact the merged meta-dataset with bladder tissues included more NMIBC than MIBC samples, and *ANXA5* was shown to be downregulated at the early stages, but to be upregulated at the higher stages [150]. Therefore, this protein has been suggested to be a marker of the low- to high-grade stage transition of tumors in BCa. *ANXA5*, along with the Annexin family members, was found to be aberrantly expressed and highly connected with BCa prognosis [151]. More specifically, high expression of *ANXA5* was found to be correlated with poor disease-free and progression-free survival times, indicating that it may be involved in the recurrence and progression of BCa. In another study, the unfavorable prognostic value of *ANXA5* was verified and its high expression was linked with the basal-subtype MIBC [152]. *ANXA5* was also found to be differentially expressed in a variety of other cancers, such as breast cancer [153], hepatocellular carcinoma [154], and lung squamous cell carcinoma [155]. *ANXA5* is considered a predictive biomarker for tumor development, progression, invasion, and metastasis, and is suggested to be of diagnostic, prognostic, and therapeutic importance in cancer [156].

Chromatin licensing and DNA replication factor one (*CDT1*) constitutes a licensing factor that operates so as to restrict DNA from replicating over once per cell cycle. Particularly, CDT1 protein is implicated in the formation of the pre-replication complex which is required for DNA replication. *CDT1* is inhibited by geminin, preventing the assembly of the pre-replication complex and the replication initiation at inappropriate origins. CDT1 protein is phosphorylated by cyclin A-dependent kinases resulting in its degradation. Hence, *CDT1* is highly associated with the cell cycle, cell division, DNA replication, and mitosis. In our study, *CDT1* was overexpressed in bladder tissue and urine of BCa patients, which was also confirmed by X. C. Mo et al. [157], whereas it was significantly under-expressed in the blood plasma of BCa patients. *CDT1* has been considered to contribute to cell proliferation and genome instability [158] and to be often misregulated in cancer [159]. Furthermore, when expressed at a high level, it was linked with poor survival and prognosis in breast cancer [160], hepatocellular carcinoma [161], colon [162], and prostate cancer [163]. Overexpression of *CDT1* is connected with irregular cell replication, activation of DNA damage checkpoints, and predisposition to malignant transformation in various human cancers. The aberrant expression of *CDT1* in BCa and its concomitant diagnostic and prognostic relevance remains to be furtherly elucidated.

Collagen type III alpha one chain (*COL3A1*) encodes the pro-alpha one chains of type III collagen, a fibrillary collagen protein that occurs in most soft connective tissues, such as arteries, skin, and soft organs, frequently along with type I collagen. It is an essential extracellular matrix-related gene, as its monomers cross-assemble into thicker fibrils, which aggregate to form fibers, providing a strong support structure for tissues requiring tensile strength and playing an essential role in their extensibility [164]. *COL3A1* levels were reported to be remarkably upregulated in high-grade and MIBC compared to low-grade and NMIBC cases, and this high expression was linked with shorter disease-free survival [120] as well as with worse overall survival [165]. *COL3A1* was also found to be among the hub genes associated with the progression of NMIBC to MIBC [121]. Notably, the expression levels of *COL3A1* were reported to be lower among patients with NMIBC [166] and higher among patients with invasive disease, contributing to tumor progression and metastasis [167,168]. This is consistent with our meta-analysis results in which *COL3A1* was found to be downregulated in bladder tissue, as the number of NMIBC samples was higher compared to MIBC. In contrast to this, *COL3A1* was found to be under-expressed in urine samples as well, despite the fact that the majority of these patients had high-grade urothelial carcinomas. Future studies are needed in order to shed light on *COL3A1*′s role in the development of BCa and in the progression from NMIBC to MIBC. 

Collagen type VI alpha one chain (*COL6A1*) encodes the pro-alpha one chain of type VI collagen and belongs to the superfamily of collagen proteins, as *COL3A1*. Collagens are extracellular matrix proteins and play an important role in sustaining the integrity of various tissues. Namely, collagen VI acts as a cell-binding protein and is involved in the cell adhesion process. *COL6A1*′s elevated expressions were significantly correlated with worse overall survival in BCa patients [165]. In a recent bioinformatics analysis [169], *COL6A1* was found to be a risk indicator for high progression of BCa and negatively associated with the patient’s prognosis. In the same study, it was also stated that it may be used as an individual effective diagnostic and prognostic biomarker for BCa, along with five other collagen family members. In our study, *COL6A1* levels were found to be significantly downregulated in the blood plasma and urinary bladder tissue among BCa patients, substantiating previous findings in the literature [170]. In the latter study, it was suggested that *COL6A1* and *COL6A2* may act as standard collagens by constructing a physical barrier to inhibit BCa tumor growth and invasion. According to a study that applied comparative urine proteomics profiling from prostate cancer patients, COL6A1 protein had a highly confirmed involvement in prostate cancer as well [171]. Urine and blood levels of collagens may hold a potential diagnostic and prognostic value for BCa and should be properly investigated, especially in the context of the extracellular matrix–tumor interaction. Collagen is an essential constituent of the tumor microenvironment, as it participates in cancer fibrosis. Thus, the comprehension of its structural properties and pathophysiological functions in human cancers may lead to the development of novel anticancer therapies [172].

Secreted phosphoprotein one (*SPP1*), commonly known as osteopontin (*OPN*), is a major non-collagenous extracellular matrix structural protein and an organic component of bone. The SPP1 protein participates in the osteoclast attachment to the mineralized extracellular bone matrix. Apart from *SPP1*′s beneficial roles in wound healing and bone homeostasis, *SPP1* is considered to be involved in several pathophysiological processes including cancer progression and metastasis, acting as a cardinal mediator of tumor-associated inflammation [173], as well as immunomodulation [174]. It was found to be significantly upregulated among bladder tumor samples in various previous studies [175,176] and to indicate poor prognosis in relation to advanced disease stage [177]. *SPP1* also proved to be markedly overexpressed in the bladder tissue and serum of transitional cell carcinoma patients [178] and in MIBC patients compared to healthy individuals [81], which is consistent with our results. It has also been suggested that *SPP1* may be an effective therapeutic or diagnostic target in certain cancers, such as melanoma, breast, colorectal, head and neck, and lung cancer, as it appeared to correlate with poor clinical outcomes and promote tumor progression by interacting with carcinogenic genes and facilitating immune cell infiltration [179,180]. Significantly, expression of *SPP1* showed a subtype-dependent effect on chemotherapy response [75], which was also confirmed in our analysis. More specifically, we found that patients who had higher *SPP1* expression levels showed a lower response to cisplatin-based chemotherapy, which is also supported by previous evidence from the literature and research on other types of cancers, such as lung [181] and ovarian cancer [182]. Results from another study found that *SPP1* was upregulated in upper tract urothelial carcinoma cells and tissues, and high plasma *SPP1* expression levels were strongly connected with higher stage and grade [183]. Considering all the above, it is suggested that circulating *SPP1* levels may be a potential biomarker for identifying BCa patients and predicting invasive disease and therapy response. Further research is required to explore its exact molecular mechanisms in BCa and to assess its value as a biomarker.

Vascular endothelial growth factor A (*VEGFA*) is a member of the vascular endothelial growth factor (VEGF) and placental growth factor (PGF) family, which both play essential and complementary roles in angiogenesis. This gene encodes a heparin-binding protein, which constitutes a disulfide-linked glycosylated homodimer. It provokes proliferation and migration of vascular endothelial cells, comprising a key regulator of both physiological and pathological angiogenesis. *VEGFA* has long been recognized as a potential vascular and proliferative therapeutic target in cancer patients and it has revealed innovative therapeutic approaches in oncology [184]. Particularly, *VEGFA* is overexpressed in many known tumors, including BCa, and its expression has been associated with tumor stage and progression as well as the patient’s prognosis [185]. *VEGFA*’s levels were reported to be highly expressed in BCa [186], and it was also recognized as a key candidate gene in BCa and as a gene related to the prognosis of patients with BCa [187], which was also confirmed by our results. Although the prognostic role of *VEGFA* in BCa remains controversial, most studies converge on the fact that patients with higher tissue or urine *VEGFA* levels showed worse outcomes in both overall and disease-free survival [177,188,189,190], which does not appear to be corroborated by the results from TCGA-BCa cohort. In a study conducted by Z. Zhong and M. Huang et al., it was suggested that *MYLK*, which was described as a key hub gene for BCa by our study, might function as a competing endogenous RNA promoting BCa progression through modulating *VEGFA*/*VEGFR2* signaling pathway [191]. *VEGFA* was previously proposed, along with other ELISA-detected markers such as *IL8* and *MMP9*, as a urinary biomarker that can accurately detect primary or recurrent BCa [105,192,193]. There is evidence to suggest that plasma levels of *VEGFA* hold value as a potential diagnostic and prognostic biomarker for BCa patients.

Cell division cycle associated 8 (*CDCA8*) encodes Borealin/Dasra B, which is a crucial protein component of the chromosomal passenger complex (CPC), an important dynamic structure that functions as a key regulator during mitosis. In particular, *CDCA8* is necessary for the kinetochore attachment-error correction and for the stability of the bipolar spindle in human mitosis and, in disease states, it can contribute to distant metastasis of cancer cells. *CDCA8* was reported as a potential prognostic biomarker for a variety of cancers, including breast [194], liver [195], and prostate cancer [196]. In a recent study by X. Gao et al., results found that *CDCA8* was upregulated in BCa in contrast to normal tissues, and its high expression was highly associated with the unfavorable prognosis of patients [197], findings which were also highlighted by other authors [198]. In the same study, it was shown that through the *CDCA8* expression inhibition, the proliferation, migration, and invasion of BCa cell lines were also inhibited and the apoptosis of cells was induced. What is more, S. Pan et al. found that *CDCA8*, along with *KIF11*, *NCAPG*, and *NEK2*, played an essential role in the maintenance of BCa stem cells [199]. K. Chen et al. reported that *CDCA8*, together with *CENPF*, *AURKB*, *CCNB2*, *CDC20*, *TTK*, and *ASPM*, were considered hub genes for BCa and verified their prognostic value [200]. In addition, it was indicated that *CDCA8* in conjunction with *MKI67*, *CENPA*, *AURKB*, *FOXM1*, and *DLGAP5*, were among the top hub genes with regard to BCa [201]. In a current bioinformatics study [202], *CDCA8* and *CDC20* were identified as candidate diagnostic biomarkers for BCa. Its essential role in BCa was also supported by S. Li et al., who suggested that lower expression of *CDCA8*, *TOP2A*, *CENPF,* and *FOXM1*, were associated with favorable overall survival of BCa patients [203]. *CDCA8* was also proposed by J. Shi et al. to be a candidate gene in NMIBC [204]. The aforementioned genes were all indicated as key hub genes for BCa by our study and the previous findings in the literature confirm our results. In our analysis, high *CDCA8* expression levels were found to significantly deteriorate the overall survival of BCa patients (Table 5), but to be associated with better overall survival of the MIBC patients receiving cisplatin-based chemotherapy. Despite the fact that there was some inconsistency, chemotherapy response is connected with a multitude of parameters. The reason for this inconsistency could be owed to the presence of more basal/squamous-like subtypes in the GSE169455 dataset, which have been suggested to suppress chemotherapy efficacy [205], or to the *CDCA8*′s association with the immune cell infiltration, which could be a predictive biomarker for chemotherapy responsiveness [206,207]. Hence, there is growing evidence that *CDCA8* may constitute an effective therapeutic target for prognosis and treatment of BCa, but its exact biological function remains still obscure and needs to be clarified.

Holliday junction recognition protein (*HJURP*) is a centromeric histone chaperone required for the histone H3-like variant centromere protein A (*CENPA*) recruitment and its deposition at centromeres during the early G1 phase. More explicitly, *HJURP* is a cell cycle regulated factor responsible for the maintenance and deposition of *CENPA* at centromeres [208]. Apart from *CENPA*-containing chromatin assembly, it is involved in the regulation of DNA binding activity, chromosomal segregation, cell mitosis, and regulation of protein-containing complex assembly. Our analysis confirmed previous bioinformatics studies in which *HJURP* was suggested to be a key hub gene for BCa [157,209]. R. Cao et al. found that *HJURP* is highly overexpressed in BCa tissues at both mRNA and protein levels and suggested that *HJURP* might regulate cell proliferation and apoptosis in BCa by acting on the *PPARγ-SIRT1* negative feedback loop [210]. Other studies reported that *HJURP* levels were significantly higher in cancerous than those in normal tissues in pancreatic [211], lung [212], breast [213], prostate [214], and renal cell cancer [215], and its high expression was liked with poor survival. Its oncogenic role was also investigated in a recent pan-cancer analysis by R. Su et al., validating the aforementioned findings [216]. In our study, *HJURP* was found overexpressed in BCa tissues and its expression levels were reported lower in the blood plasma of BCa patients. The role of *HJURP* in tumor development, and especially in BCa, is still unclear and it remains to be elucidated, as it appears that *HJURP* could serve as a novel diagnostic and prognostic biomarker for the management of BCa.

DNA topoisomerase IIα (*TOP2A*) encodes a key nuclear enzyme that regulates the state of DNA during transcription, generates DNA single-strand breaks, and induces gene transcription during cell division. Accordingly, *TOP2A* is involved in chromosome formation, enrichment, and separation, in DNA replication and transcription, and it is suggested to be involved in the development of several cancer types. *TOP2A* was reported to be one of the top 10 hub genes identified in BCa, along with *VEGFA*, *CCNB1*, *CDC20*, *AURKB*, *UBE2C*, and *CCNB2* [187]. Furthermore, S. Zeng et al. found that *TOP2A* was highly overexpressed in BCa, especially in high-grade and advanced-stage tumors, and its overexpression was highly connected with worse cancer-specific, progression-free, and recurrence-free survival [217]. In the latter study, it was proved that the proliferation of BCa cells was especially inhibited by the knockdown of *TOP2A*, and their migration and invasion capacity was strongly suppressed. In the same line, F. Zhang and H. Wu found that by inhibiting *TOP2A* the BCa tumorigenesis is repressed [218]. In our study, *TOP2A* was found to be significantly overexpressed in the urine of the BCa patients compared to controls. These results correlate fairly well with findings by W. T. Kim et al. [219] and further support the concept of urinary cell-free nucleic acids that may be complementary diagnostic biomarkers for BCa. *TOP2A* was previously confirmed to be more abundant in the urine of patients with BCa than in the urine of controls, using the Western blotting technique [220]. G. Botti et al. reviewed the effective utility of ProEx C, an immunohistochemical reagent incorporating *TOP2A* and *MCM2* antibodies, as an assistant tool in evaluating the urothelial lesions in urine cytology and stated that it could accurately differentiate high-grade lesions from benign and reactive conditions [221]. It is remarkable that *MCM2* was found to be significantly downregulated in the blood plasma of BCa patients in our study (Figure 13). Notably, the *TOP2A*/*MCM2* combination was reported to be the best biomarker for discriminating between low- and high-grade squamous intraepithelial lesions for cervical cancer [222]. Additionally, *TOP2A* expression levels were found to be significantly different between patients who completely responded to therapy and those who do not in our study. Previous studies have reported that *TOP2A* constitutes a marker for predicting prognosis and response to various cancer therapies, for instance, breast cancer [223], soft tissue sarcomas [224], as well as clear cell renal cell carcinoma [225]. There are strong indications that *TOP2A* plays a functional role in the BCa proliferation and invasion, as well as in patients’ response to the disease, which remain to be proved.

The nine identified potential key biomarker genes were employed as features in classification models, aiming to distinguish the cancerous and normal samples. These models showed a very high prediction accuracy for the vast majority of the utilized datasets, indicating that these genes may be used as potential diagnostic biomarkers in BCa. The protein expression of these genes in cancerous and normal urinary bladder tissues was confirmed by immunohistochemistry from the HPA. Overall, we highlighted the findings and main points of our study in Table 7. The results underscore the need for validation of these promising BCa biomarkers in independent pre-clinical settings.

It is plausible that there were a number of limitations that could have influenced the results of the present study and should be declared. The first is the fact that the 606 bladder tissue samples that were meta-analyzed did not originate from the corresponding number of patients. In many studies, case and control samples were collected from the same patient, from cancerous and adjacent healthy tissues (matched pairs), which could introduce some intra-subject correlations. Additionally, there were studies that performed sample pooling prior to hybridization. Secondly, the inevitable technical sources of variation confounded our analysis either when they were corrected or not. We tried to handle them properly and justify the followed methodology in each case. Another considerable limitation was the fact that there were no in vivo experiments conducted in order to validate the potential functions and mechanisms of the identified genes in the development and progression of BCa. Moreover, it should be underlined that BCa constitutes a highly heterogeneous malignancy, which is composed of various molecular subtypes. The identification of DEGs, as well as genes with prognostic and predictive value, may considerably vary depending on the specific subtype under investigation. However, in this study, we aimed to identify the main hub genes that aggregately differentiate BCa cells from the normal ones. Last but not least, this study integrated mRNA gene expression data derived solely from microarray experiments and validated some results using bioinformatics tools that incorporate RNA-Seq or IHC data. Further studies including a wider range of data types, such as non-coding RNA, DNA methylation, and RNA-Seq data, are planned to be performed.

## 5. Conclusions

In conclusion, this study aspired to contribute to the elucidation of the genetic changes occurring in BCa, using systematic bioinformatic tools and methods. In particular, we successfully integrated gene expression data from multiple datasets and we identified a list of hub genes that appear to play an essential role in the development and progression of BCa. A subset of these genes, namely *ANXA5*, *CDT1*, *COL3A1*, *SPP1*, *VEGFA*, *CDCA8*, *HJURP*, *TOP2A*, and *COL6A1*, was associated with altered gene expression in urine or blood plasma of patients and were highlighted as potential diagnostic markers for BCa. Moreover, the study revealed a three-gene signatures (*COL3A1*, *FOXM1*, and *PLK4*) that achieved high prognostic performance in relation to the overall survival of BCa patients, and a six-gene signature (*ANXA5*, *CD44*, *NCAM1*, *SPP1*, *CDCA8*, and *KIF14*) that showed satisfactory predictive performance in terms of disease-free survival of MIBC patients receiving cisplatin-based NAC. Further research is needed to validate the clinical value of these biomarkers and their potential in BCa treatment.

## Figures and Tables

**Table 1:** Characteristics of the 18 individual series included in the integrative meta-analysis.

GEO Accession	Samples (*n*)			Year	Platform	Sample Characteristics	Reference
	Total	BCa	Controls				
**GSE3167**	60	46	14	2005	GPL96 (HG-U133A) Affymetrix Human Genome U133A Array	13 superficial transitional cell carcinomas with surrounding CIS 15 without surrounding CIS lesions13 muscle-invasive carcinomas 5 CISs 5 CISs 14 normal bladder tissues	[82]
**GSE7476**	12	9	3	2007	GPL570 (HG-U133_Plus_2) Affymetrix Human Genome U133 Plus 2.0 Array	3 groups of 5 pooled Ta tumors 1 group of 5 pooled T1 tumors 2 groups of 4 pooled T1 tumors 3 groups of 5 pooled T2+ tumors 3 groups of 4 pooled normal bladder tissues	[83]
**GSE13507**	232	170	62	2010	GPL6102 Illumina human-6 v2.0 expression beadchip	24 primary Ta cancer tissues 62 primary T1 cancer tissues 31 primary T2 cancer tissues 19 primary T3 cancer tissues 11 primary T4 cancer tissues 23 recurrent NMIBC tissues 62 normal bladder tissues	[64]
**GSE21142**	24	12	12	2013	GPL10274 Affymetrix GeneChip Human Genome U133 Plus 2.0 Array (Brainarray CustomCDF, GU133Plus2_Hs_UG_Version 12.cdf)	6 superficial urothelial carcinomas 6 invasive urothelial carcinomas 12 normal bladder tissues	[84]
**GSE23732**	8	7	1	2012	GPL6244 (HuGene-1_0-st) Affymetrix Human Gene 1.0 ST Array (transcript (gene) version)	7 muscle-invasive bladder cancer 1 normal bladder tissue	-
**GSE24152**	17	10	7	2010	GPL6791 Affymetrix GeneChip Human Genome U133 Plus 2.0 Array (CDF: Hs_ENTREZG_10)	10 muscle-invasive urothelial bladder carcinomas 7 benign bladder tissues	[85]
**GSE31189**	92	52	40	2013	GPL570 (HG-U133_Plus_2) Affymetrix Human Genome U133 Plus 2.0 Array	52 urothelial cancer cells 40 normal urothelial cells	[86]
**GSE37815**	24	18	6	2013	GPL6102 Illumina human-6 v2.0 expression beadchip	18 NMIBC tissues 6 normal bladder tissues	[87]
**GSE38264**	38	28	10	2014	GPL6244 (HuGene-1_0-st) Affymetrix Human Gene 1.0 ST Array (transcript (gene) version)	28 Ta and T1 tumors 10 normal bladder tissues	[88]
**GSE40355**	24	16	8	2013	GPL13497 Agilent-026652 Whole Human Genome Microarray 4x44K v2 (Probe Name version)	8 Ta urothelial carcinoma tissues 5 T1 urothelial carcinoma tissues 3 T2 urothelial carcinoma tissues 8 normal bladder tissue samples	[89]
**GSE41614**	10	5	5	2013	GPL5175 (HuEx-1_0-st) Affymetrix Human Exon 1.0 ST Array (transcript (gene) version)	2 samples with blood vessels from T1 bladder cancer tissue 2 samples with blood vessels from T2 bladder cancer tissue 1 sample with blood vessels from T3 bladder cancer tissue 5 samples with blood vessels from normal bladder	[90]
**GSE42089**	18	10	8	2013	GPL9828 (HG-U133_Plus_2) Affymetrix Human Genome U133 Plus 2.0 Array (CDF: Brainarray Hs133P_Hs_ENTREZG version 10)	10 urothelial cell carcinomas 8 normal bladder tissues	[91]
**GSE45184**	6	3	3	2013	GPL14550 Agilent-028004 SurePrint G3 Human GE 8x60K Microarray (Probe Name Version)	3 bladder cancer tissues 3 normal adjacent tissues	[92]
**GSE52519**	12	9	3	2013	GPL6884 Illumina HumanWG-6 v3.0 expression beadchip	1 T1 cancerous tissue sample 2 T2 cancerous tissue samples 2 T3 cancerous tissue samples 4 T4 cancerous tissue samples 3 normal bladder tissues	[93]
**GSE65635**	12	8	4	2015	GPL14951 Illumina HumanHT-12 WG-DASL V4.0 R2 expression beadchip	5 T1 bladder cancer tissues 2 T3 bladder cancer tissues 1 T4 bladder cancer tissues 4 normal bladder tissues	[93]
**GSE76211**	6	3	3	2017	GPL17586 (HTA-2_0) Affymetrix Human Transcriptome Array 2.0 (transcript (gene) version)	3 T3 bladder cancer tissues 3 normal bladder tissues	[94]
**GSE100926**	6	3	3	2017	GPL14550 Agilent-028004 SurePrint G3 Human GE 8x60K Microarray (Probe Name Version)	2 T2 MIBC tissues 1 T3 MIBC tissue 3 normal bladder tissues	[95]
**GSE121711**	18	8	10	2019	GPL17586 (HTA-2_0) Affymetrix Human Transcriptome Array 2.0 (transcript (gene) version)	3 Ta primary tumors 2 T1 primary tumors 3 T2 primary tumors 10 normal bladder tissues	[96]
**Total**	**619**	**417**	**202**	--	--	--	--

**Table 2:** Performance parameters of the various classification models for the different set of DEGs. Bold: the one with the highest AUC.

|log_2_FC|	No of Features (DEGs)	AUC	Sensitivity	Specificity
1	1295	0.9525	0.7964	0.9366
1.1	1099	0.9517	0.7934	0.9327
1.2	929	0.9527	0.7842	0.9346
**1.3**	**815**	**0.9531**	**0.7985**	**0.9334**
1.4	725	0.9510	0.7996	0.9322
1.5	625	0.9516	0.8022	0.9342
1.6	549	0.9487	0.7929	0.9278
1.7	495	0.9482	0.7844	0.9312
1.8	442	0.9510	0.7966	0.9298
1.9	407	0.9519	0.8001	0.9288
2.0	364	0.9507	0.7903	0.9356

**Table 3:** Hub genes as obtained from cytoHubba and MCODE plugins of Cytoscape.

A. Genes Included in the Final Ranked List Aggregated from the 10 Topological cytoHubba Methods			
*IL6*, *VEGFA*, *CCNB1*, *BRCA1*, *CCNA2*, *CD44*, *TYMS*, *CDH1*, *LMNB1*, *AURKB*, *EZH2*, *MKI67*, *KIF23*, *ECT2*, *MCM4*, *CDC6*, *PLK1*, *CDC25C*, *CDKN3*, *CENPA*, *MMP2*, *TOP2A*, *CENPE*, *PBK*, *NDC80*, *FOXM1*, *SPP1*, *IGF1*, *UBE2C*, *RRM2*, *KIF11*, *CHEK1*, *CD8A*, *CCNB2*, *ASPM*, *NCAM1*, *FLNA*, *LGALS4*, *ITPR1*, *DLGAP5*, *CDCA8*, *COL5A1*, *TIMELESS*, *CDC20*, *DMD*, *PPARGC1A*, *WNT5A*, *BUB1*, *KIF20A*, *EXO1*, *CDC25A*, *VCL*, *LUM*, *CCND2*, *CD34*, *MCM2*, *MAD2L1*, *HPGDS*, *ISL1*, *ESRP1*, *SKP2*, *NCAPG*, *CENPU*, *HJURP*, *CCL2*, *TPM1*, *CDH11*, *PLK4*, *FABP4*, *H2AFX*, *GJA1*, *DHCR7*, *PTGS2*, *MSN*, *ANXA5*, *COL6A1*, *TRIP13*, *OIP5*, *MYH11*, *KRT20*, *TTK*, *MYL9*, *CAV1*, *FBXO5*, *PROM1*, *BMP4*, *CDT1*, *KIAA0101*, *CCNE1*, *ANXA1*, *FGFR3*, *SNCA*, *ATAD2*, *ESPL1*, *FASN*, *NT5E*, *ZWINT*, *SDC1*, *FGF2*, *NEK2*, *ACTG2*, *KIF14*, *COL3A1*, *EPCAM*, *ASF1B*, *IGFBP5*, *RAD54L*, *CYP1B1*, *STMN1*, *COL4A5*, *ATF3*, *CASC5*, *CENPM*, *ERBB3*, *DNMT3B*, *ITGB2*, *ISG15*, *ANK2*, *CDC45*, *PLAT*, *TACC3*, *EGR1*, *MYLK*, *CTSG*, *GINS2*, *ITGA8*, *CENPF*, *TGFBR2*, *OGN*			
**B. Genes included in the first three clusters of MCODE**			
**Cluster**	**Score**	**Nodes**	**Gene clusters**
1	74.268	83	*PLK4*, *TRIP13*, *CDC45*, *PBK*, *RRM2*, *ERCC6L*, *CHAF1A*, *DEPDC1*, *DLGAP5*, *ASPM*, *E2F8*, *MAD2L1*, *CDCA8*, *CCNB1*, *BRCA1*, *FANCI*, *FBXO5*, *CENPA*, *KIAA0101*, *TK1*, *TACC3*, *DTL*, *CDCA3*, *HJURP*, *CENPE*, *ZWINT*, *ESPL1*, *POLQ*, *OIP5*, *CDC25C*, *ASF1B*, *CDKN3*, *POLE2*, *CCNB2*, *CHAF1B*, *EZH2*, *UBE2C*, *RAD54L*, *CDT1*, *MCM5*, *CDC20*, *TROAP*, *CKS2*, *NEK2*, *SPC25*, *MKI67*, *CHEK1*, *TTK*, *CDC6*, *GINS2*, *BUB1*, *CENPU*, *CCNE2*, *STIL*, *KIF14*, *TYMS*, *CDC7*, *MCM2*, *KIF23*, *KNTC1*, *SKA1*, *CASC5*, *CENPF*, *HELLS*, *NUSAP1*, *ATAD2*, *CEP55*, *NCAPG*, *MCM4*, *NDC80*, *ECT2*, *TOP2A*, *CENPM*, *CDC25A*, *MCM10*, *ORC1*, *KIF20A*, *AURKB*, *CCNA2*, *PLK1*, *EXO1*, *FOXM1*, *KIF11*
2	18	23	*CXCL12*, *PTGS2*, *BMP4*, *IL6*, *GJA1*, *CD34*, *FGF2*, *NES*, *PROM1*, *CD8A*, *VEGFA*, *CD44*, *SDC1*, *SPP1*, *ANXA5*, *NCAM1*, *SELP*, *CCL2*, *CCL5*, *IGF1*, *CSF1R*, *NT5E*, *SELE*
3	7.923	27	*TGFBI*, *COL6A2*, *THBS2*, *TPM1*, *MYH11*, *ACTG2*, *COL6A1*, *COL13A1*, *COL3A1*, *TGFBR2*, *VCL*, *FBLN2*, *COL4A5*, *CTSK*, *LYVE1*, *CLDN5*, *ANGPT2*, *LUM*, *MYL9*, *LEPREL1*, *TPM2*, *SPARC*, *MYLK*, *CAV1*, *ADAMTS5*, *TAGLN*, *FMOD*
**C. Common genes between cytoHubba and MCODE**			
*IL6*, *VEGFA*, *CCNB1*, *BRCA1*, *CCNA2*, *CD44*, *TYMS*, *AURKB*, *EZH2*, *MKI67*, *KIF23*, *ECT2*, *MCM4*, *CDC6*, *PLK1*, *CDC25C*, *CDKN3*, *CENPA*, *TOP2A*, *CENPE*, *PBK*, *NDC80*, *FOXM1*, *SPP1*, *IGF1*, *UBE2C*, *RRM2*, *KIF11*, *CHEK1*, *CD8A*, *CCNB2*, *ASPM*, *NCAM1*, *DLGAP5*, *CDCA8*, *CDC20*, *BUB1*, *KIF20A*, *EXO1*, *CDC25A*, *VCL*, *LUM*, *CD34*, *MCM2*, *MAD2L1*, *NCAPG*, *CENPU*, *HJURP*, *CCL2*, *TPM1*, *PLK4*, *GJA1*, *PTGS2*, *ANXA5*, *COL6A1*, *TRIP13*, *OIP5*, *MYH11*, *TTK*, *MYL9*, *CAV1*, *FBXO5*, *PROM1*, *BMP4*, *CDT1*, *KIAA0101*, *ATAD2*, *ESPL1*, *NT5E*, *ZWINT*, *SDC1*, *FGF2*, *NEK2*, *ACTG2*, *KIF14*, *COL3A1*, *ASF1B*, *RAD54L*, *COL4A5*, *CASC5*, *CENPM*, *CDC45*, *TACC3*, *MYLK*, *GINS2*, *CENPF*, *TGFBR2*			

**Table 4:** The key hub genes of our study are defined as the intersection of hub genes between PPI network analysis and WGCNA.

Module	Common Hub Genes
turquoise	*ACTG2*, *ANXA5*, *AURKB*, *BUB1*, *CD34*, *CD44*, *CDC25A*, *CDT1*, *CENPM*, *ESPL1*, *EXO1*, *FGF2*, *GINS2*, *KIF20A*, *NCAM1*
brown	*CAV1*, *COL3A1*, *COL4A5*, *IGF1*, *LUM*, *MYLK*, *PROM1*, *SDC1*, *SPP1*, *TPM1*, *VCL*, *VEGFA*
black	*ASPM*, *CCNA2*, *CCNB1*, *CCNB2*, *CDC20*, *CDC45*, *CDCA8*, *CDKN3*, *CENPA*, *CENPF*, *CENPU*, *DLGAP5*, *ECT2*, *EZH2*, *FOXM1*, *HJURP*, *KIF11*, *KIF14*, *KIF23*, *MCM2*, *MCM4*, *MKI67*, *NCAPG*, *NDC80*, *NEK2*, *PBK*, *PLK4*, *RAD54L*, *TOP2A*, *TTK*, *UBE2C*, *ZWINT*
blue	*--*
green	*COL6A1*, *MYH11*
yellow	*--*

**Table 5:** Univariate Cox regression analysis of the survival-associated hub genes in BCa patients (HR: hazard ratio, CI: confidence interval, * *p*-value < 0.05, ** *p*-value < 0.01, *** *p*-value < 0.001, **** *p*-value < 0.0001).

	Univariate Analysis		Multivariate Analysis	
RFS Related Gene	HR (95% CI)	*p*-Value	HR (95% CI)	*p*-Value
ACTG2	1.2 (1–1.4)	1.40 × 10^−2^ *	--	--
AURKB	2.1 (1.5–3)	3.30 × 10^−5^ ****	--	--
BUB1	1.8 (1.2–2.6)	1.90 × 10^−3^ **	--	--
CDC25A	3.1 (1.6–5.9)	9.80 × 10^−4^ ***	--	--
CDT1	1.9 (1.4–2.6)	1.00 × 10^−4^ ***	--	--
CENPM	2.1 (1.4–3.1)	1.90 × 10^−4^ ***	--	--
ESPL1	2.8 (1.7–4.6)	5.30 × 10^−5^ ****	--	--
EXO1	3.8 (1.9–7.7)	2.50 × 10^−4^ ***	--	--
GINS2	1.8 (1.2–2.5)	1.40 × 10^−3^ **	--	--
KIF20A	1.8 (1.3–2.5)	6.00 × 10^−4^ ***	--	--
COL3A1	1.5 (1.1–1.9)	3.50 × 10^−3^ **	1.72 (1.29–2.29)	0.000223 ***
COL4A5	0.66 (0.51–0.85)	1.70 × 10^−3^ **	--	--
LUM	1.3 (1–1.6)	1.80 × 10^−2^ *	--	--
SPP1	1.4 (1.1–1.7)	4.80 × 10^−3^ **	--	--
ASPM	1.9 (1.4–2.6)	5.40 × 10^−5^ ****	--	--
CCNA2	1.6 (1.2–2.3)	2.50 × 10^−3^ **	--	--
CCNB1	1.7 (1.1–2.6)	1.00 × 10^−2^ *	--	--
CCNB2	1.8 (1.3–2.4)	9.90 × 10^−5^ ****	--	--
CDC20	1.7 (1.3–2.3)	1.90 × 10^−4^ ***	--	--
CDC45	2.7 (1.7–4.2)	1.30 × 10^−5^ ****	--	--
CDCA8	2.2 (1.5–3.2)	2.40 × 10^−5^ ****	--	--
CDKN3	2.1 (1.5–3)	3.40 × 10^−5^ ****	--	--
CENPA	1.8 (1.3–2.5)	2.70 × 10^−4^ ***	--	--
CENPF	2 (1.5–2.7)	7.50 × 10^−6^ ****	--	--
CENPU	1.7 (1.1–2.6)	2.20 × 10^−2^ *	--	--
DLGAP5	1.7 (1.2–2.3)	1.20 × 10^−3^ **	--	--
ECT2	3 (1.5–6.2)	3.00 × 10^−3^ **	--	--
EZH2	2.2 (1.4–3.4)	5.30 × 10^−4^ ***	--	--
FOXM1	2.7 (1.8–4)	3.20 × 10^−6^ ****	5.34 (2.95–9.64)	2.87 × 10^−8^ ****
HJURP	2.1 (1.5–2.9)	4.30 × 10^−5^ ****	--	--
KIF11	1.9 (1.3–2.8)	6.00 × 10^−4^ ***	--	--
KIF14	2.4 (1.5–3.7)	1.40 × 10^−4^ ***	--	--
KIF23	3 (1.5–5.7)	1.20 × 10^−3^ **	--	--
MCM2	1.7 (1.2–2.3)	1.70 × 10^−3^ **	--	--
MCM4	1.4 (1–1.9)	3.80 × 10^−2^ *	--	--
MKI67	9.8 (4–24)	8.60 × 10^−7^ ****	--	--
NCAPG	2.1 (1.5–3)	6.40 × 10^−5^ ****	--	--
NDC80	1.5 (1.1–2.1)	1.30 × 10^−2^ *	--	--
NEK2	2.5 (1.4–4.5)	2.40 × 10^−3^ **	--	--
PBK	1.7 (1.2–2.4)	2.50 × 10^−3^ **	--	--
PLK4	1.6 (1–2.5)	3.90 × 10^−2^ *	0.38 (0.19–0.80)	0.010188 *
RAD54L	2.3 (1.5–3.4)	4.40 × 10^−5^ ****	--	--
TOP2A	1.5 (1.2–1.9)	1.50 × 10^−3^ **	--	--
TTK	1.8 (1.3–2.4)	5.50 × 10^−4^ ***	--	--
UBE2C	2.3 (1.5–3.6)	2.50 × 10^−4^ ***	--	--
ZWINT	3 (1.4–6)	3.00 × 10^−3^ **	--	--

**Table 6:** Univariate Cox regression analysis of the survival-associated hub genes in MIBC patients receiving neoadjuvant cisplatin-based chemotherapy (HR: hazard ratio, CI: confidence interval, * *p*-value < 0.05, ** *p*-value < 0.01, *** *p*-value < 0.001, **** *p*-value < 0.0001).

	Univariate Analysis		Multivariate Analysis	
RFS-Related Gene	HR (95% CI)	*p*-Value	HR (95% CI)	*p*-Value
ANXA5	1.1 (0.70–1.70)	0.7	0.42 (0.24–0.74)	0.00268 **
CD44	1.3 (0.99–1.70)	0.055	1.65 (1.20–2.28)	0.00220 **
NCAM1	1.1 (0.81–1.60)	0.48	1.60 (1.09–2.35)	0.01718 *
IGF1	0.48 (0.24–0.98)	0.043 *	--	--
SPP1	1.5 (1.30–1.80)	3.3 × 10^−6^ ****	1.72 (1.42–2.09)	2.93 × 10^−8^ ****
CDCA8	0.5 (0.26–0.96)	0.037 *	0.18 (0.08–0.42)	5.72 × 10^−5^ ****
KIF14	1.8 (0.87–3.70)	0.11	4.68 (2.17–10.11)	8.59 × 10^−5^ ****
**CSS-Related Gene**	**HR (95% CI)**	***p*** **-Value**		
ANXA5	1.1 (0.68–1.70)	0.74	0.44 (0.24–0.82)	0.009708 **
CD44	1.3 (0.95–1.60)	0.11	1.60 (1.12–2.29)	0.009785 **
NCAM1	1.1 (0.82–1.60)	0.47	1.43 (0.99–2.05)	0.049989 *
SPP1	1.4 (1.20–1.70)	5.8 × 10^−5^ ****	1.64 (1.35–2.01)	1.15 × 10^−6^ ****
CDCA8	0.48 (0.24–0.94)	0.034 *	0.17 (0.07–0.41)	6.49 × 10^−5^ ****
KIF14	1.7 (0.84–3.60)	0.14	4.82 (2.12–10.96)	0.000171 ***
**OS-Related Gene**	**HR (95% CI)**	***p*** **-Value**		
ACTG2	1.3 (1.00–1.60)	0.038 *	--	--
ANXA5	0.97 (0.63–1.50)	0.9	0.41 (0.23–0.72)	0.002139 **
CD44	1.2 (0.96–1.60)	0.095	1.63 (1.17–2.28)	0.003812 **
NCAM1	1.1 (0.82–1.50)	0.48	1.42 (1.00–2.01)	0.047272 *
SPP1	1.4 (1.20–1.60)	0.00012 ***	1.60 (1.32–1.92)	1.1 × 10^−6^ ****
CDCA8	0.48 (0.25–0.91)	0.024 *	0.19 (0.09–0.42)	5.0 × 10^−5^ ****
KIF14	1.6 (0.78–3.20)	0.21	4.45 (2.01–9.85)	0.000225 ***

**Table 7:** The highlights of this study at a glance.

Highlights of This Study
The analysis of the merged microarray meta-dataset, comprising of 410 BCa and 196 healthy urinary bladder tissue samples from 18 independent datasets, revealed 815 robust differentially expressed genes (DEGs). A total of 61 key hub genes resulted from DEG-based protein–protein interaction (PPI) and weighted gene co-expression (WGCNA) network analyses. A subset of key hub genes, namely *AURKB*, *CCNB2*, *CDC45*, *CDCA8*, *CDT1*, *CENPU*, *COL3A1*, *GINS2*, *KIF20A*, *MCM4*, *PBK*, *PLK4*, *SDC1*, *SPP1*, *TOP2A*, *TTK*, and *UBE2C*, were found to be differentially expressed in the urine of BCa patients. A subset of key hub genes, namely *ANXA5*, *ASPM*, *CD34*, *CDC20*, *CDT1*, *COL4A5*, *COL6A1*, *ECT2*, *HJURP*, *MCM2*, and *VEGFA*, were found to be differentially expressed in the blood plasma of BCa patients. Bioinformatics tools and machine learning techniques were utilized to reveal and assess the diagnostic, prognostic, and predictive value of the identified key hub genes. A three-gene signature prognostic model for BCa patients, including *COL3A1*, *FOXM1*, and *PLK4*, was built and demonstrated high performance. A six-gene signature predictive model regarding MIBC patients’ response to neoadjuvant chemotherapy, including *ANXA5*, *CD44*, *NCAM1*, *SPP1*, *CDCA8*, and *KIF14*, was developed and showed satisfactory performance. Overall, nine genes, namely *ANXA5*, *CDT1*, *COL3A1*, *SPP1*, *VEGFA*, *CDCA8*, *HJURP*, *TOP2A*, and *COL6A1*, were identified as potential prognostic and therapeutic target biomarkers for BCa, they were immunohistochemically validated using Human Protein Atlas (HPA), and were bibliographically analyzed.

## Data Availability

All data are available from the GEO repository. The present analysis is available after a reasonable request to the first author.

## Supplementary Materials

The following supporting information can be downloaded at: https://www.mdpi.com/article/10.3390/cancers14143358/s1, Figure S1: PCA on each of the 18 datasets, using Euclidean distance to measure dissimilarities over gene expression values between samples. Red points denote BCa samples and blue points denote control samples; Figure S2: PCA on datasets GSE13507A and GSE13507B. Red points denote BCa samples and blue points denote control samples; Figure S3: Volcano plot of DEGs between BCa and control samples in the merged meta-dataset. The DEGs were identified based on the criteria |log_2_(FC)| ≥ 1.3 and adj. *p*-value < 0.01, as represented with gray lines. The blue and red points denote downregulated genes and upregulated genes, respectively. The black points denote genes showing no statistically significant difference in expression between the two phenotypes; Figure S4: Summary of structural network indices in relation to the soft thresholding power for each of the eight selected datasets; Figure S5: Hierarchical clustering dendrogram of genes for determining consensus modules based on consensus topological overlap. Genes in a common module are assigned the same color, as presented in the color band below the dendrogram. Genes not assigned to any of the modules are colored gray; Figure S6: Heatmaps relationships of consensus module eigengenes and phenotypic traits across the eight datasets. Each row corresponds to a consensus module eigengene and, each column corresponds to a phenotypic characteristic. Each cell contains the corresponding correlation (ranging from blue to red) and *p*-value; Figure S7: Scatter plots of gene significance (GS) for “tumor” and module membership (MM) for the key modules. The lines indicate the upper and the lower quartile; Figure S8: Expression correlation analysis of the three genes in the prognostic model, obtained from the GEPIA2 platform. The Pearson correlation coefficient was used, and indicated that there is no statistically significant correlation coefficient among these genes (maximum value 0.6); Figure S9: Expression correlation analysis of the six genes in the predictive model, obtained from the GEPIA2 platform. The Pearson correlation coefficient was used and indicated that there is no statistically significant correlation coefficient among these genes (maximum value 0.44).
