# Supplementary material for: An Integrated Bioinformatics Analysis towards the Identification of Diagnostic, Prognostic, and Predictive Key Biomarkers for Urinary Bladder Cancer
Source: Cancers (Basel). 2022 Jul 10;14(14):3358. doi: 10.3390/cancers14143358 (PMC9319344; doi:10.3390/cancers14143358)
Supplement: Supplementary file 1 [file cancers-14-03358-s001.zip › Figure S1.pdf]

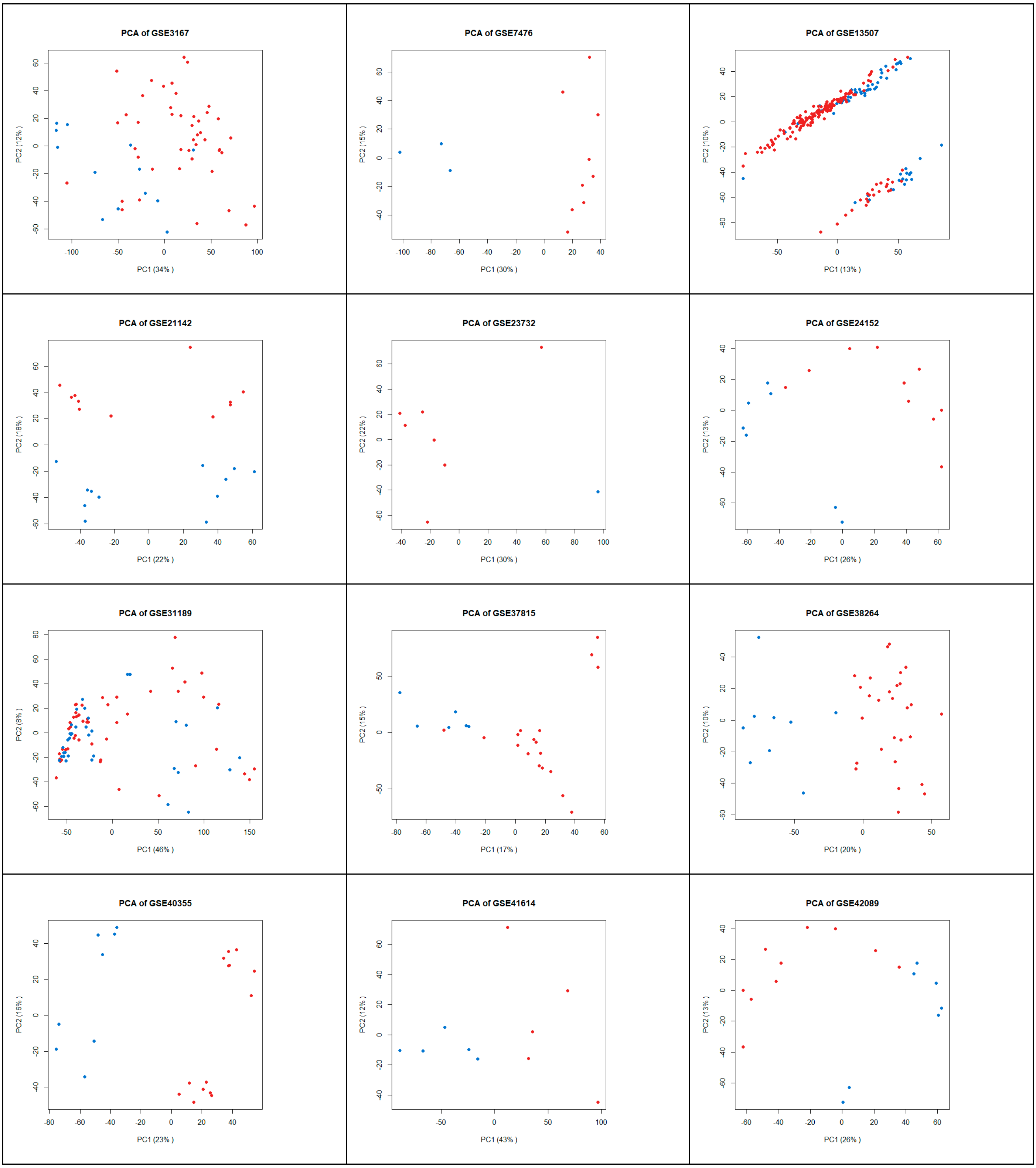
PCA of GSE3167
PC1 (34%)
PC2 (12%)
PCA of GSE7476
PC1 (30%)
PC2 (15%)
PCA of GSE13507
PC1 (13%)
PC2 (10%)
PCA of GSE21142
PC1 (22%)
PC2 (18%)
PCA of GSE23732
PC1 (30%)
PC2 (22%)
PCA of GSE24152
PC1 (26%)
PC2 (13%)
PCA of GSE31189
PC1 (46%)
PC2 (8%)
PCA of GSE37815
PC1 (17%)
PC2 (15%)
PCA of GSE38264
PC1 (20%)
PC2 (10%)
PCA of GSE40355
PC1 (23%)
PC2 (16%)
PCA of GSE41614
PC1 (43%)
PC2 (12%)
PCA of GSE42089
PC1 (26%)
PC2 (13%)

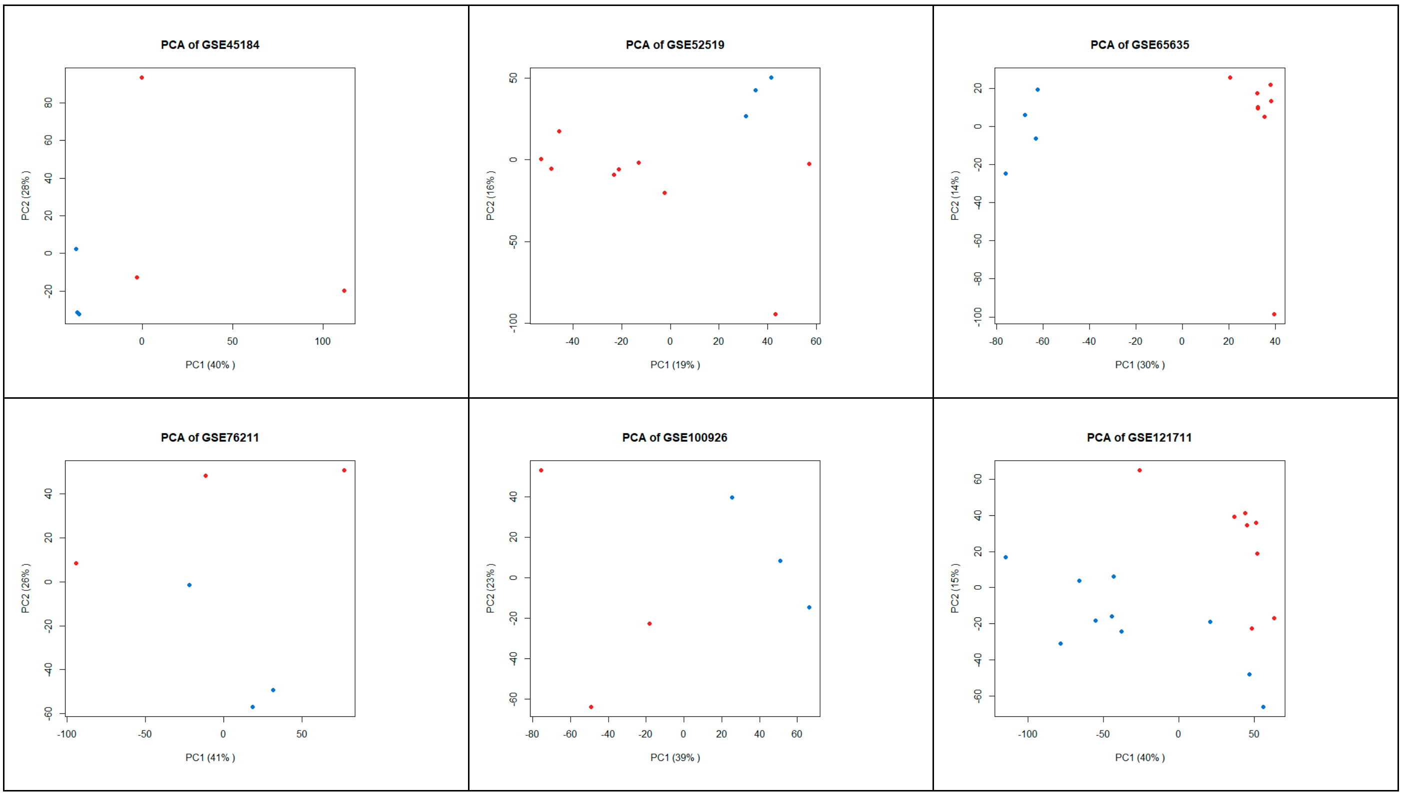

Figure S1. PCA on each of the 18 datasets, using Euclidean distance to measure dissimilarities over gene expression values between samples. Red points denote BCa samples and blue points denote control samples.
