# Supplementary material for: An Integrated Bioinformatics Analysis towards the Identification of Diagnostic, Prognostic, and Predictive Key Biomarkers for Urinary Bladder Cancer
Source: Cancers (Basel). 2022 Jul 10;14(14):3358. doi: 10.3390/cancers14143358 (PMC9319344; doi:10.3390/cancers14143358)
Supplement: Supplementary file 1 [file cancers-14-03358-s001.zip › Figure S3.pdf]

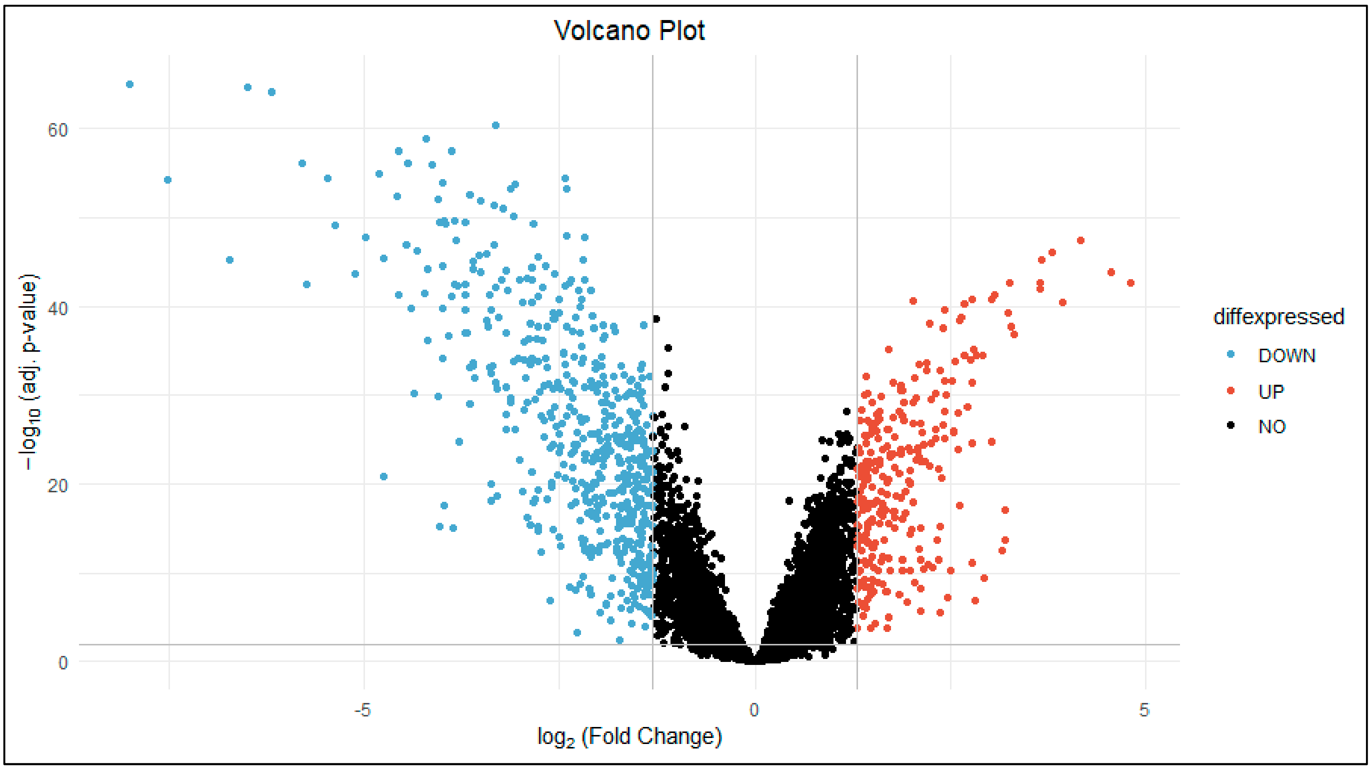

Figure S3. Volcano plot of DEGs between BCa and control samples in the merged meta-dataset. The DEGs were identified based on the criteria $|log_2(FC)| \geq 1.3$ and adj. p-value < 0.01, as represented with grey lines. The blue and red points denote downregulated genes and upregulated genes, respectively. The black points denote genes showing no statistically significant difference in expression between the two phenotypes.
