# Supplementary material for: An Integrated Bioinformatics Analysis towards the Identification of Diagnostic, Prognostic, and Predictive Key Biomarkers for Urinary Bladder Cancer
Source: Cancers (Basel). 2022 Jul 10;14(14):3358. doi: 10.3390/cancers14143358 (PMC9319344; doi:10.3390/cancers14143358)
Supplement: Supplementary file 1 [file cancers-14-03358-s001.zip › Figure S5.pdf]

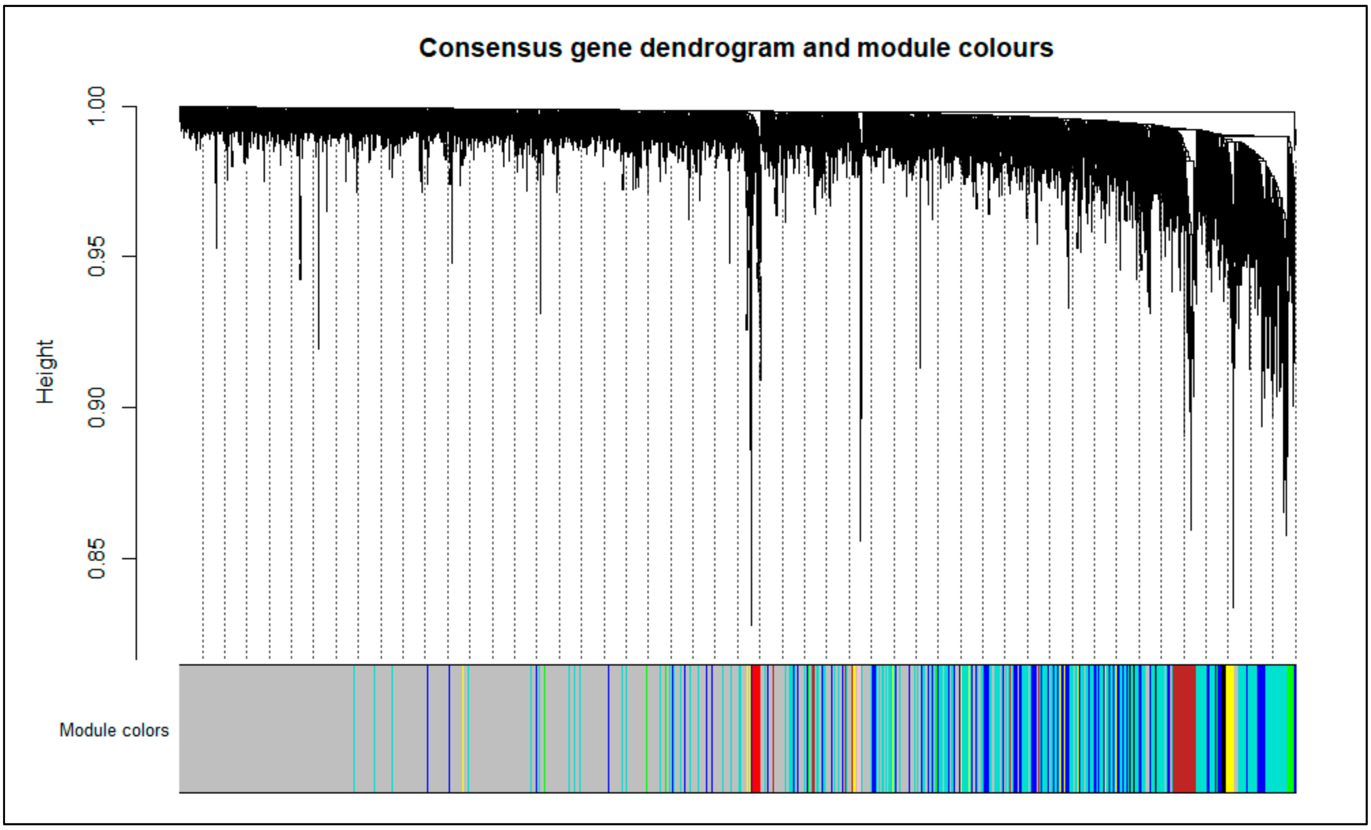

Figure S5. Hierarchical clustering dendrogram of genes for determining consensus modules based on consensus Topological Overlap. Genes in a common module are assigned the same colour, as presented in the colour band below the dendrogram. Genes not assigned to any of the modules are coloured grey.
