# Supplementary material for: An Integrated Bioinformatics Analysis towards the Identification of Diagnostic, Prognostic, and Predictive Key Biomarkers for Urinary Bladder Cancer
Source: Cancers (Basel). 2022 Jul 10;14(14):3358. doi: 10.3390/cancers14143358 (PMC9319344; doi:10.3390/cancers14143358)
Supplement: Supplementary file 1 [file cancers-14-03358-s001.zip › Figure S6.pdf]

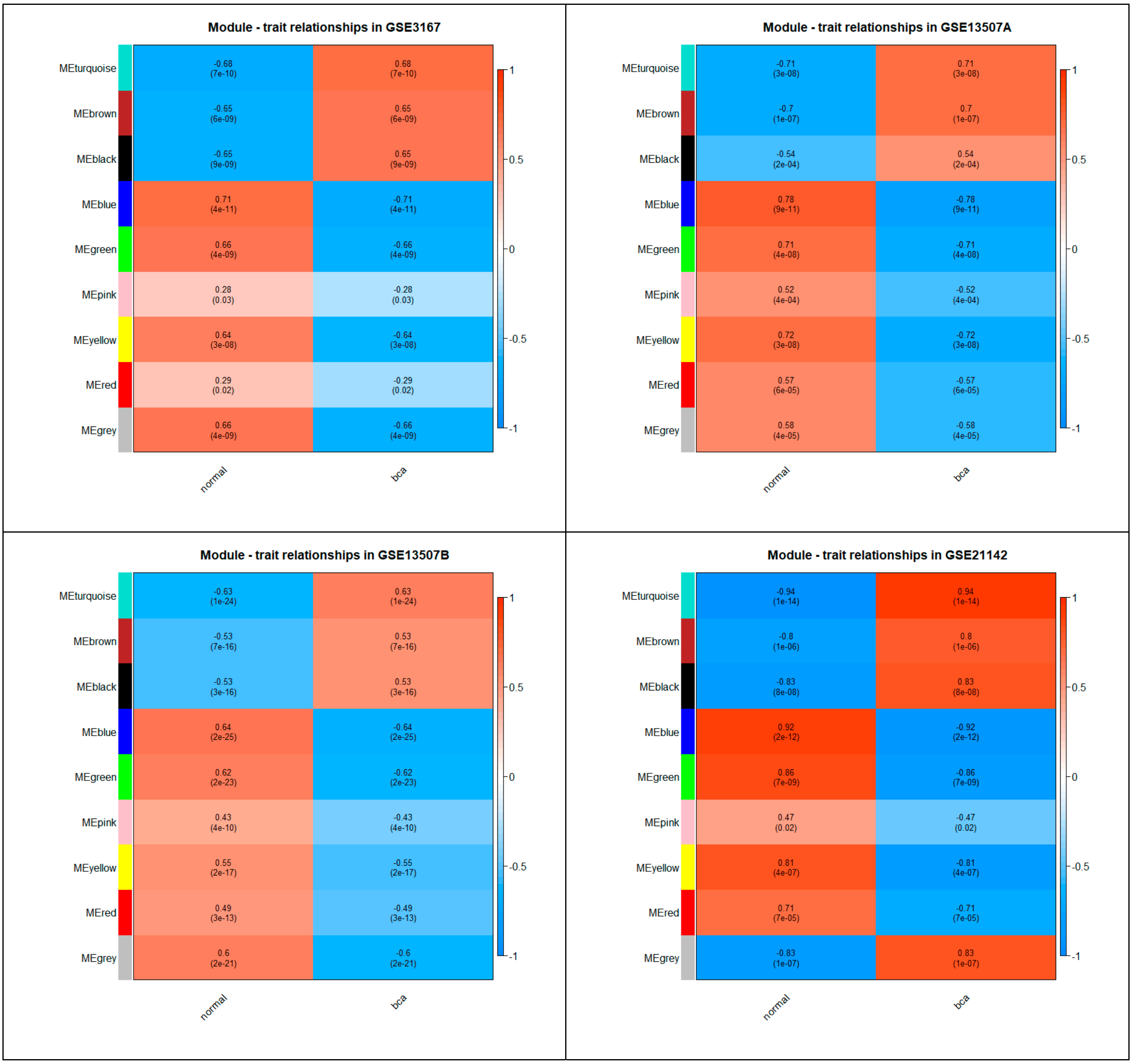
Module - trait relationships in GSE3167
MEturquoise
MEbrown
MEblack
MEblue
MEgreen
MEpink
MEyellow
MEred
MEgrey
-0.68 (7e-10)
0.68 (7e-10)
-0.65 (6e-09)
0.65 (6e-09)
-0.65 (9e-09)
0.65 (9e-09)
0.71 (4e-11)
-0.71 (4e-11)
0.66 (4e-09)
-0.66 (4e-09)
0.28 (0.03)
-0.28 (0.03)
0.64 (3e-08)
-0.64 (3e-08)
0.29 (0.02)
-0.29 (0.02)
0.66 (4e-09)
-0.66 (4e-09)
normal
bca
1
0.5
0
-0.5
-1
Module - trait relationships in GSE13507A
MEturquoise
MEbrown
MEblack
MEblue
MEgreen
MEpink
MEyellow
MEred
MEgrey
-0.71 (3e-08)
0.71 (3e-08)
-0.7 (1e-07)
0.7 (1e-07)
-0.54 (2e-04)
0.54 (2e-04)
0.78 (9e-11)
-0.78 (9e-11)
0.71 (4e-08)
-0.71 (4e-08)
0.52 (4e-04)
-0.52 (4e-04)
0.72 (3e-08)
-0.72 (3e-08)
0.57 (6e-05)
-0.57 (6e-05)
0.58 (4e-05)
-0.58 (4e-05)
normal
bca
1
0.5
0
-0.5
-1
Module - trait relationships in GSE13507B
MEturquoise
MEbrown
MEblack
MEblue
MEgreen
MEpink
MEyellow
MEred
MEgrey
-0.63 (1e-24)
0.63 (1e-24)
-0.53 (7e-16)
0.53 (7e-16)
-0.53 (3e-16)
0.53 (3e-16)
0.64 (2e-25)
-0.64 (2e-25)
0.62 (2e-23)
-0.62 (2e-23)
0.43 (4e-10)
-0.43 (4e-10)
0.55 (2e-17)
-0.55 (2e-17)
0.49 (3e-13)
-0.49 (3e-13)
0.6 (2e-21)
-0.6 (2e-21)
normal
bca
1
0.5
0
-0.5
-1
Module - trait relationships in GSE21142
MEturquoise
MEbrown
MEblack
MEblue
MEgreen
MEpink
MEyellow
MEred
MEgrey
-0.94 (1e-14)
0.94 (1e-14)
-0.8 (1e-06)
0.8 (1e-06)
-0.83 (8e-08)
0.83 (8e-08)
0.92 (2e-12)
-0.92 (2e-12)
0.86 (7e-09)
-0.86 (7e-09)
0.47 (0.02)
-0.47 (0.02)
0.81 (4e-07)
-0.81 (4e-07)
0.71 (7e-05)
-0.71 (7e-05)
-0.83 (1e-07)
0.83 (1e-07)
normal
bca
1
0.5
0
-0.5
-1

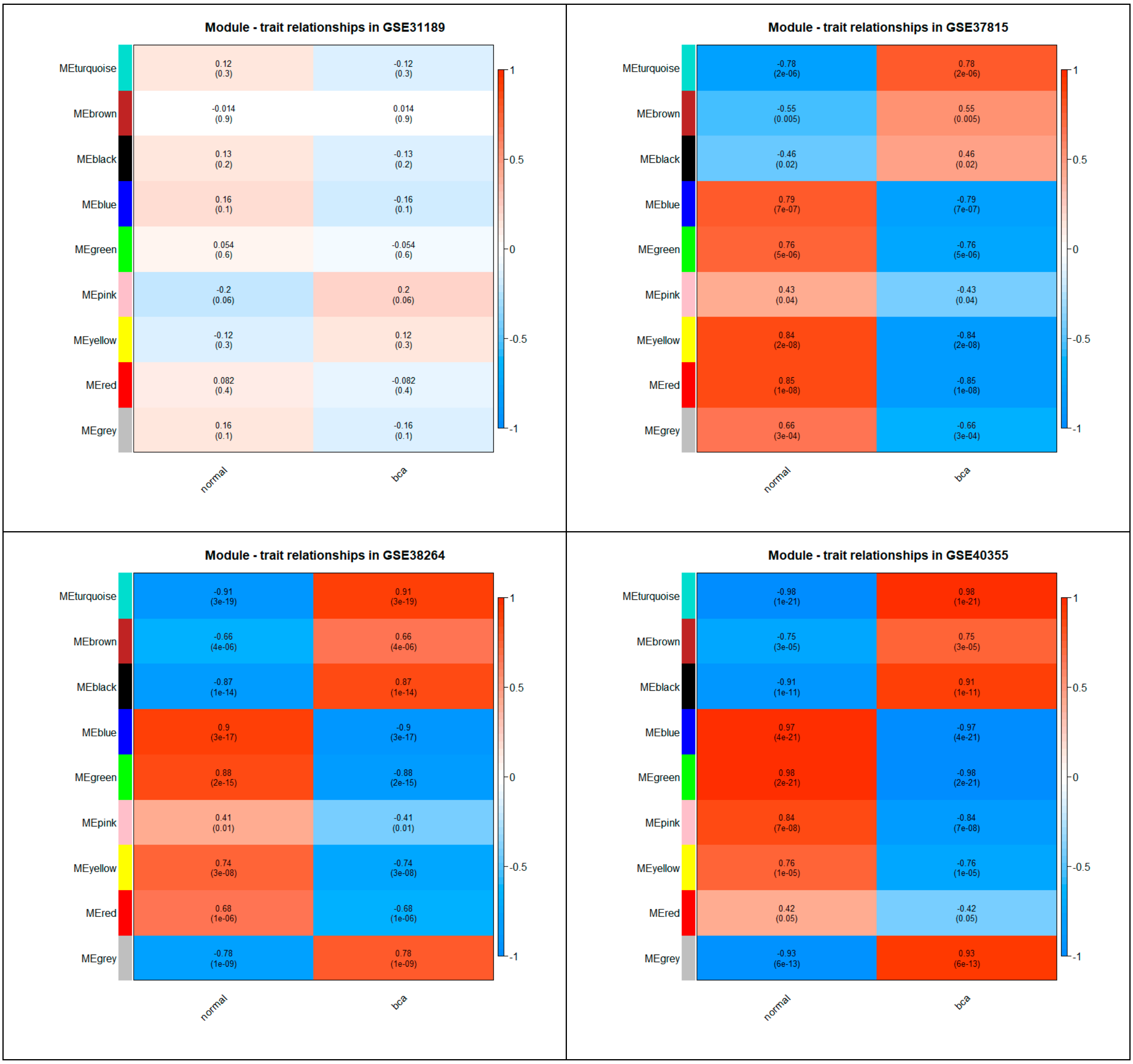

Figure S6. Heatmaps relationships of consensus module eigengenes and phenotypic traits across the eight datasets. Each row corresponds to a consensus module eigengene and, each column corresponds to a phenotypic characteristic. Each cell contains the corresponding correlation (ranging from blue to red) and p-value.
