# Supplementary material for: An Integrated Bioinformatics Analysis towards the Identification of Diagnostic, Prognostic, and Predictive Key Biomarkers for Urinary Bladder Cancer
Source: Cancers (Basel). 2022 Jul 10;14(14):3358. doi: 10.3390/cancers14143358 (PMC9319344; doi:10.3390/cancers14143358)
Supplement: Supplementary file 1 [file cancers-14-03358-s001.zip › Figure S7.pdf]

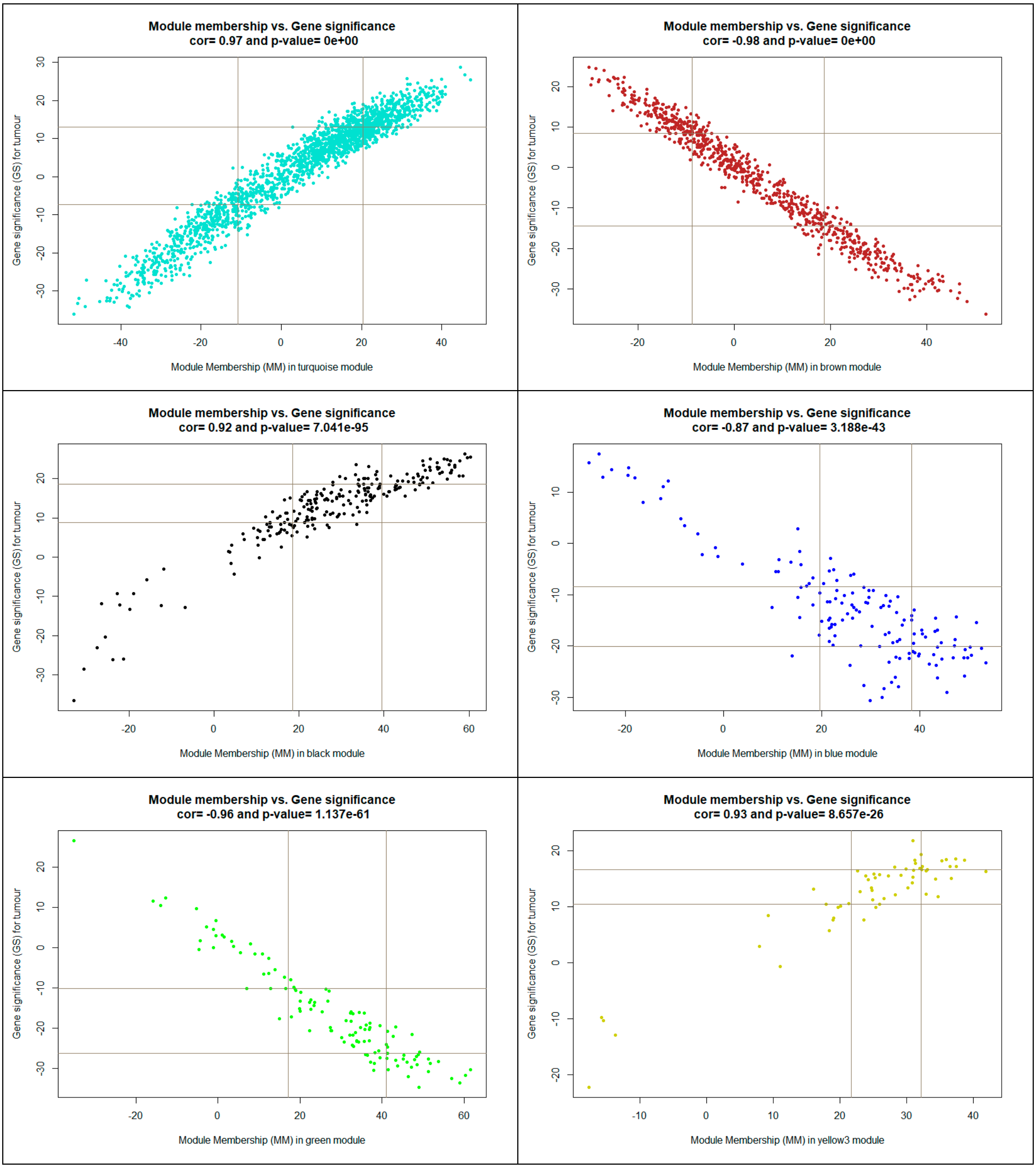

Figure S7. Scatter plots of gene significance (GS) for "tumour" and module membership (MM) for the key modules. The lines indicate the upper and the lower quartile.
