# Supplementary material for: An Integrated Bioinformatics Analysis towards the Identification of Diagnostic, Prognostic, and Predictive Key Biomarkers for Urinary Bladder Cancer
Source: Cancers (Basel). 2022 Jul 10;14(14):3358. doi: 10.3390/cancers14143358 (PMC9319344; doi:10.3390/cancers14143358)
Supplement: Supplementary file 1 [file cancers-14-03358-s001.zip › Figure S9.pdf]

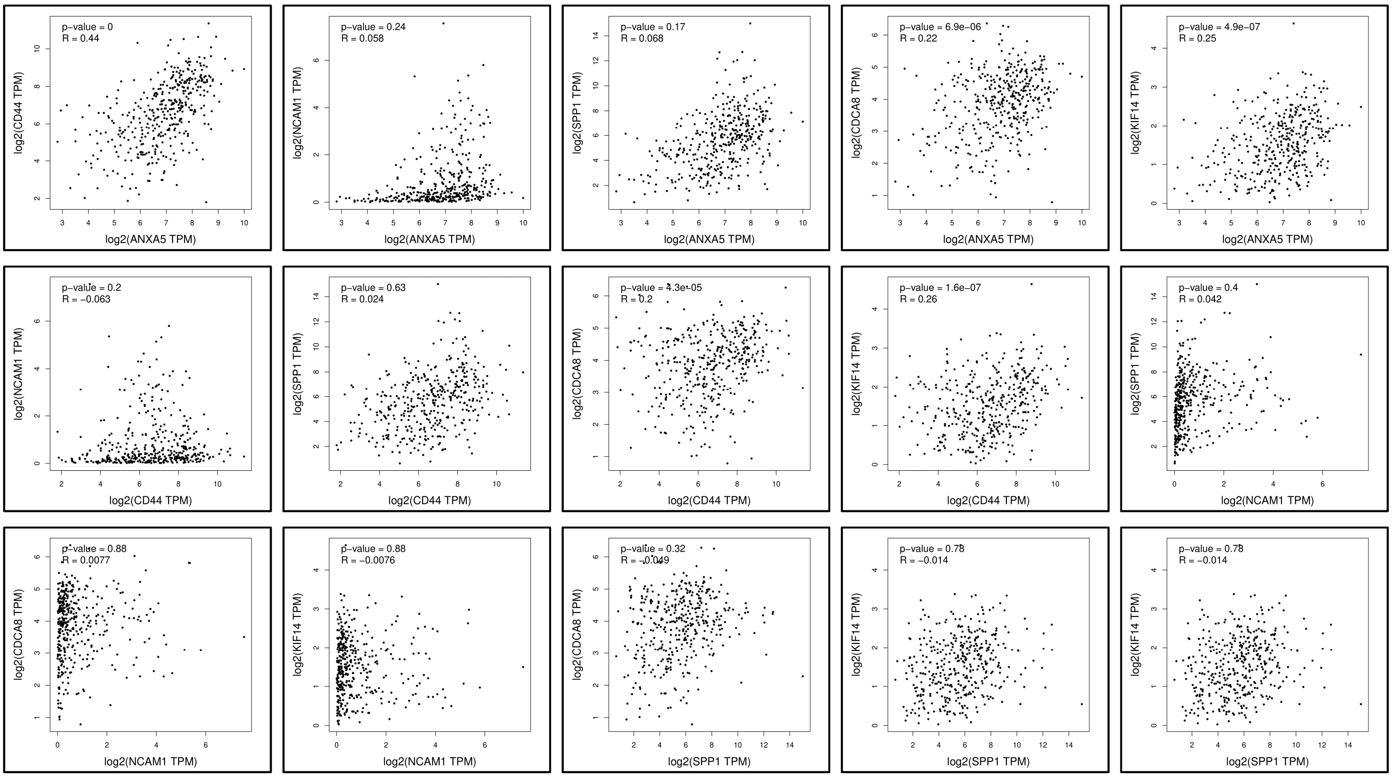

Figure S9. Expression correlation analysis of the six genes in the predictive model, obtained from the GEPIA2 platform. The Pearson correlation coefficient was used, and indicated that there is no statistically significant correlation coefficient among these genes (maximum value 0.44).
